# Latent class analysis to predict intensive care outcomes in Acute Respiratory Distress Syndrome: a proposal of two pulmonary phenotypes

**DOI:** 10.1186/s13054-021-03578-6

**Published:** 2021-04-22

**Authors:** Pedro D. Wendel Garcia, Alessio Caccioppola, Silvia Coppola, Tommaso Pozzi, Arianna Ciabattoni, Stefano Cenci, Davide Chiumello

**Affiliations:** 1 Institute of Intensive Care Medicine, University Hospital of Zurich, Zurich, Switzerland; 2 Department of Anesthesia and Intensive Care, ASST Santi Paolo E Carlo, San Paolo University Hospital, Via Di Rudinì, Milan, Italy; 3 Department of Health Sciences, University of Milan, Milan, Italy; 4 Coordinated Research Center on Respiratory Failure, University of Milan, Milan, Italy

**Keywords:** ARDS, Latent class analysis, Phenotypes, Mechanical ventilation, Respiratory mechanics, Radiological data, Recruitment, Enrichment

## Abstract

**Background:**

Acute respiratory distress syndrome remains a heterogeneous syndrome for clinicians and researchers difficulting successful tailoring of interventions and trials. To this moment, phenotyping of this syndrome has been approached by means of inflammatory laboratory panels. Nevertheless, the systemic and inflammatory expression of acute respiratory distress syndrome might not reflect its respiratory mechanics and gas exchange.

**Methods:**

Retrospective analysis of a prospective cohort of two hundred thirty-eight patients consecutively admitted patients under mechanical ventilation presenting with acute respiratory distress syndrome. All patients received standardized monitoring of clinical variables, respiratory mechanics and computed tomography scans at predefined PEEP levels. Employing latent class analysis, an unsupervised structural equation modelling method, on respiratory mechanics, gas-exchange and computed tomography-derived gas- and tissue-volumes at a PEEP level of 5cmH_2_O, distinct pulmonary phenotypes of acute respiratory distress syndrome were identified.

**Results:**

Latent class analysis was applied to 54 respiratory mechanics, gas-exchange and CT-derived gas- and tissue-volume variables, and a two-class model identified as best fitting. Phenotype 1 (*non-recruitable*) presented lower respiratory system elastance, alveolar dead space and amount of potentially recruitable lung volume than phenotype 2 (*recruitable*). Phenotype 2 (*recruitable*) responded with an increase in ventilated lung tissue, compliance and PaO_2_/FiO_2_ ratio (*p* < 0.001), in addition to a decrease in alveolar dead space (*p* < 0.001), to a standardized recruitment manoeuvre. Patients belonging to phenotype 2 (*recruitable*) presented a higher intensive care mortality (hazard ratio 2.9, 95% confidence interval 1.7–2.7, *p* = 0.001).

**Conclusions:**

The present study identifies two ARDS phenotypes based on respiratory mechanics, gas-exchange and computed tomography-derived gas- and tissue-volumes. These phenotypes are characterized by distinctly diverse responses to a standardized recruitment manoeuvre and by a diverging mortality. Given multicentre validation, the simple and rapid identification of these pulmonary phenotypes could facilitate enrichment of future prospective clinical trials addressing mechanical ventilation strategies in ARDS.

**Supplementary Information:**

The online version contains supplementary material available at 10.1186/s13054-021-03578-6.

## Background

The first description of the acute respiratory distress syndrome (ARDS) dates back more than 50 years [1]. Since then, multiple attempts have followed to provide the ideal definition for this syndrome [2, 3]. Nonetheless and albeit the definition of ARDS as acute hypoxemia concomitant to diffuse bilateral lung infiltrates of non-cardiac aetiology should have unified diagnosis and treatment of ARDS, an ever-growing body of research has proven its poor ability to effectively detect the syndrome in first place [4–6].

The pathophysiology of ARDS is characterized by an intense lung inflammation caused by the highly heterogeneous interplay between host and insult, depending on the aetiology of the latter [7]. This heterogeneity in ARDS presentation mainly reflects on the greatly varying severity of hypoxemia, amount of lung oedema, timing of onset and underlying cause of disease, as well as on the presence of the histopathological hallmark of ARDS: diffuse alveolar damage [2, 8–11]. It is thus not surprising that most randomized controlled trials targeting ARDS in its entirety have failed [2, 12].

In order to identify more homogeneous subgroups of ARDS, several subclassifications have been proposed based on simple variables such as the severity of hypoxemia or the level of positive end-expiratory pressure (PEEP) applied [13, 14]. Consideration of these subsets of ARDS has enabled the design of successful interventional randomized control trials [15, 16]. Recently, latent class analysis (LCA), a well-validated statistical method that is able to identify clusters of similar patients [17], has been used to describe two distinct phenotypes of ARDS characterized by a different degree of inflammatory response [18, 19]. These *hypo-* and *hyperinflammatory* phenotypes have been associated with contrasting natural histories, biological characteristics and outcomes to clinical and pharmacological interventions [19, 20].

Predictive and prognostic enrichment of immunomodulatory trials in ARDS by considering inflammatory phenotypes may potentially prove ground-breaking. Nevertheless, the cornerstone of ARDS therapy—mechanical ventilation—is mainly governed by respiratory mechanics and gas exchange, for which paucity of information regarding ARDS phenotyping exists.

The aim of this study was to identify different phenotypes of ARDS considering respiratory mechanics, gas-exchange and computed tomography (CT)-derived gas- and tissue-volumes by implementing the LCA methodology, further assessing the natural history and response to a standardized recruitment manoeuvre of these phenotypes.

## Methods

### Population

Retrospective analysis of a cohort of prospectively and consecutively admitted patients diagnosed with ARDS at intensive care unit (ICU) admission between 2003 and 2017 to the Ospedale Policlinico Maggiore (Milan) and from 2017 to 2019 to the Azienda Socio Sanitaria Territoriale Santi Paolo e Carlo (Milan); sub-cohorts of these patients were already included into seven published trials [10, 21–26]. The institutional review boards of both hospitals approved the data collection plan and informed consent was obtained according to the respective hospital regulations.

Patients were enrolled if they met the “American-European Consensus Conference on ARDS” criteria between 2003 and 2012 [27], and if they met the “Berlin” criteria from 2012 onwards [3]. Exclusion criteria were an age of less than 16 years, pregnancy and chronic obstructive pulmonary disease.

At intensive care unit (ICU) admission, all patients were sedated and paralyzed, mechanically ventilated with a tidal volume between 6 and 8 mL of ideal body weight and PEEP and the fraction of inspired oxygen (FiO_2_) were titrated in order to obtain an arterial oxygen saturation between 93 and 98%. Patients underwent a standardized recruitment manoeuvre coupled to two prespecified CT studies (inspiratory hold at 5 and 45 cmH_2_O) and a PEEP trial (5 and 15 cmH_2_O) a median of 2 [1–4] days after intubation. Further specifications on the collection of respiratory mechanics and gas-exchange variables, performance of CT studies and recruitment manoeuvres are described in Additional file 1: Appendix 1.

### Missing data

To account for missing data (Additional file 1: Table S1), multiple imputation by fully conditional specification with predictive mean matching was performed under the missing at random assumption [28]. For each variable, a unique linear regression model was specified; all 118 independent variables recorded in the data set were included. Ten parallel imputation models with 1000 iterations each were run. Quality of imputation models was assessed by analysis of mean and standard deviation convergence plots and comparison of distribution plots for every imputation model and imputed variable (Additional file 1: Figures S1, S2). Finally, for every model and variable, *t* tests and standard mean differences (SMD) between imputed and original distribution were calculated, with SMDs below 0.1 being regarded as optimal and above 0.2 as suboptimal imputation [29, 30]. No outcome variables were imputed.

### Statistical analysis

LCA was performed using a combination of respiratory mechanics, gas-exchange characteristics and CT-derived gas- and tissue-volumes at PEEP 5 cmH_2_O as defining variables; clinical outcomes were not considered during model design and latent class analysis. In light of the scale variance between variables, and reflecting the categorical design of LCA models, all variables were refitted to an interval scale based on a decile subdivision.

Full LCA including all 54 variables violated model independence constraints; therefore, a swap-stepwise algorithm based on a Bayes factor comparison was used to select the variables best defining the latent classes [31, 32]. To avoid overfitting of the LCA model to this specific cohort, a minimal improvement of 10% in the maximum likelihood of the model was required for each swap-stepwise iteration to be accepted. The best fitting latent model regarding the number of latent classes was determined by using a combination of Bayesian information criterion (BIC), entropy, bootstrap likelihood ratio test (BLRT) inferred *p* values and class size [33, 34]. In order to assess the internal validity of the LCA and to ensure that the overall classification was not overly dependent on a sub-cohort, the LCA was refitted while repeatedly eliminating one of the sub-cohorts (~ 70 patients) based on the years of recruitment (2003–2006, 2007–2010, 2011–2014, 2015–2019) (Additional file 1: Table S2, Figure S3). Latent class analysis optimization was performed solely on the first imputation model. As imputation sensitivity analysis, latent class analysis with the final variable selection was performed on all imputation models, and imputation-dependent transition of patients between latent classes as well as diverging class inference on outcome was analysed (Additional file 1: Table S3, Figure S4). To identify a subgroup of variables with the ability to dichotomize between the latent classes obtained by means of the LCA, in a first step a least absolute shrinkage and selection operator (LASSO) method was applied. Two different variable subsets were defined for the LASSO, one including all variables considered in the LCA, and a second considering all variables but the CT inferred parameters. The meta-parameter λ in the LASSO was defined so that the final model would only contain up to four variables. The resulting two subsets of four variables each were then employed in a nested general linear regression model (GLM) analysis. To assess nested model fit and prediction performance, the Akaike information criterion (AIC) as well as receiver operating characteristics (ROC) analysis and computed area under the ROC curve (AUROC) were applied; bootstrapping was used for AUROC confidence interval (CI) calculation. First-order interaction terms between the predictor variables were tested for all models, and excluded if not improving the final model fit. A Fine and Gray competing risk analysis considering ICU mortality as primary event and alive ICU discharge as single competing event, with adjustment for SAPS II and the paO_2_/FiO_2_ ratio at PEEP 5 cmH_2_O was generated to evaluate the latent class effect on ICU-mortality [35]. Proportional hazard assumptions were assessed through inspection of Schoenfeld residuals. ICU survival functions were generated by implementing the Kaplan–Meier estimator. Only patients with complete outcome data were regarded in these analyses. Comparisons of population characteristics between classes were performed using Student’s *t* test or Wilcoxon signed-rank test, as appropriate, and the Chi-squared test for categorical variables. The specific phenotype response to recruitment manoeuvres was tested using linear mixed effects model analysis. As independent variable fixed effects, recruitment manoeuvre pressure and phenotype were entered into the model, respectively, with and without interaction terms, which were retained only if they were found to contribute to the model. As random effects, intercepts for subjects were employed. *P* values were calculated using a likelihood ratio test of the full model with the effect in question against a “null model” without the effect in question. *P* values for individual fixed effects were obtained by Satterthwaite approximation in a multi-dimensional model comprising recruitment manoeuvre pressure and phenotype. A two-sided *p* < 0.05 was considered statistically significant. For all statistical analysis, a fully scripted data management pathway was created within the R environment for statistical computing, version 3.6.1 [36]. Values are reported as median with interquartile ranges or proportions and percentages as appropriate.

## Results

### Population

A total of 238 ARDS patients were included in the analysis. The baseline characteristics are presented in Table 1. The median age and the Simplified Acute Physiology Score (SAPS) II at admission were 61 [48–73] years and 42 [33–53], respectively. The paO_2_/FiO_2_ ratio was 168 mmHg [127–213]; 29 (12%) patients presented a severe ARDS, while 74 (31%) and 135 (57%) presented a moderate and mild ARDS, respectively. The driving pressure was 13 [10–16] cmH_2_O, and respiratory system elastance was 26 [20–32] cmH_2_O/mL. Intensive care mortality amounted to 40%. Further respiratory mechanics and computed tomography data are presented in Additional file 1: Tables S4, S5 and S6.

**Table 1:** Baseline characteristics of study population

	Total population *N* = 238	Phenotype 1	Phenotype 2
		“Non-recruitable” *N* = 106	“Recruitable” *N* = 132
Age (years)	62 [48–73]	61 [49–73]	62 [49–73]
Sex (male)	162 (68)	75 (71)	87 (66)
Body mass index (kg m^−2^)	25 [22–29]	25 [23–29]	25 [22–29]
SAPS II	42 [33–53]	41 [30–52]	43 [35–52]
Vasopressors, *n* (%)	131 (55)	55 (52)	76 (58)
*Cause of ARDS, n (%)*^†^			
Aspiration	18 (8)	9 (8)	9 (7)
Pneumonia	116 (49)	37 (35)	79 (60)
Sepsis	60 (25)	35 (33)	25 (19)
Trauma	12 (5)	9 (8)	3 (2)
Other	32 (13)	9 (8)	9 (7)
*ARDS category, n (%)*^†^			
Mild	74 (31)	49 (46)	25 (19)
Moderate	135 (57)	54 (51)	81 (61)
Severe	29 (12)	3 (3)	26 (20)
PaO_2_/FiO_2_ (mmHg) ^†^	169 [127–213]	193 [156–231]	144 [112–189]
Arterial pCO_2_ (mmHg)^†^	43 [37–50]	39 [36–47]	45 [40–52]
Respiratory rate (min^−1^)^†^	16 [14–20]	15 [13–19]	17 [15–20]
Tidal volume (mL)^†^	500 [420–560]	515 [441–600]	480 [420–535]
Tidal volume/ideal body weight (mL kg^−1^)^†^	7.7 [6.7–8.7]	7.9 [7.0–9.3]	7.4 [6.6–8.3]
Clinical PEEP (cmH_2_O)	10 [10–12]	10 [10–12]	10 [10–13]
Driving pressure (cmH_2_O)^†^	13 [10–16]	12 [10–15]	17 [15–20]
Respiratory system elastance (cmH_2_O mL^−1^)^†^	26 [20–32]	22 [18–28]	28 [23–33]
Time on mechanical ventilator (days)	3 [2–6]	3 [2–6]	2 [2–5]
Intensive care unit stay (days)	18 [10–28]	18 [11–28]	18 [9–27]
ICU mortality, n (%) ^†^	96 (40)	27 (23)	69 (52)

Quantitative data are expressed as median [interquartile range] or counts (and percentages) as appropriate

*ARDS* acute respiratory distress syndrome, *BMI* body mass index, *ICU* intensive care unit, *SAPS* simplified acute physiology score

^†^*p* value < 0.05

### Identification of latent class analysis

Table 2 presents model-fit statistics for LCA models considering one to five classes. All LCA models presented an entropy above 0.8, indicating overall robust class separation. BIC was lowest for a two-class model and afterwards increased proportionally to the number of added classes, suggesting that additional classes do not add substantial information to the model. The BLRT inferred *p* value favoured the two-class model over the one-class model; additional classes did not improve model fit. In light of these findings, a two-class latent model was judged as most suitable.

**Table 2:** Fit statistics for latent class models

No. of classes	Bayesian information criteria°	Entropy*	Number of individual per class					*p* value^†^
			1	2	3	4	5	
1	10,227		238					
2	10,202	0.84	132	106				0.001
3	10,317	0.90	65	107	66			0.205
4	10,472	0.92	71	75	47	45		0.266
5	10,639	0.95	37	59	53	44	45	0.676

**°**Bayesian information criterion (BIC) is a likelihood function derived criterion for model selection among a set of models; lower BICs indicate better model fit

*Entropy is a measure to assess the degree of association between an individual and a class based on the posterior class membership probabilities; values above 0.8 define good class distinction

^†^The *p* value is calculated by means of the bootstrap likelihood ratio test; it addresses if a model with *k* classes provides increased fit compared to a model with *k *− 1 classes

The chosen two-class latent model assigned 106 (45%) patients to phenotype 1 and 132 (55%) patients to phenotype 2. The median latent class assignment probability for phenotype 1 was 100 [99.8–100] % and 99.9 [94.4–100] % for phenotype 2. In phenotype 1, 87 (82.1%) of the patients and in phenotype 2, 117 (88.6%) of the patients presented class assignment probabilities above 90%, suggesting an excellent class differentiation.

Mean imputation model effect on phenotype identification was 11 ± 2% (Additional file 1: Table S3). Further, complete case analysis of the cohort evidenced a phenotype misclassification of 7% compared to the imputed cohort; thus, imputation effect on latent class modelling was deemed small (Additional file 1: Appendix 2). Additionally, no mayor influence on outcome was patent between latent class analyses using different imputation models or complete case analysis; therefore, further results are only presented for the first imputation model (Additional file 1: Table S3, Figure S4, Appendix 2). Finally, LCA refitting while repeatedly eliminating one of the sub-cohorts based on the years of recruitment did not influence LCA fit, suggesting internal robustness of the inferred classes (Additional file 1: Table S2, Figure S3).

### Characteristics and of ARDS phenotypes

**Fig. 1:**
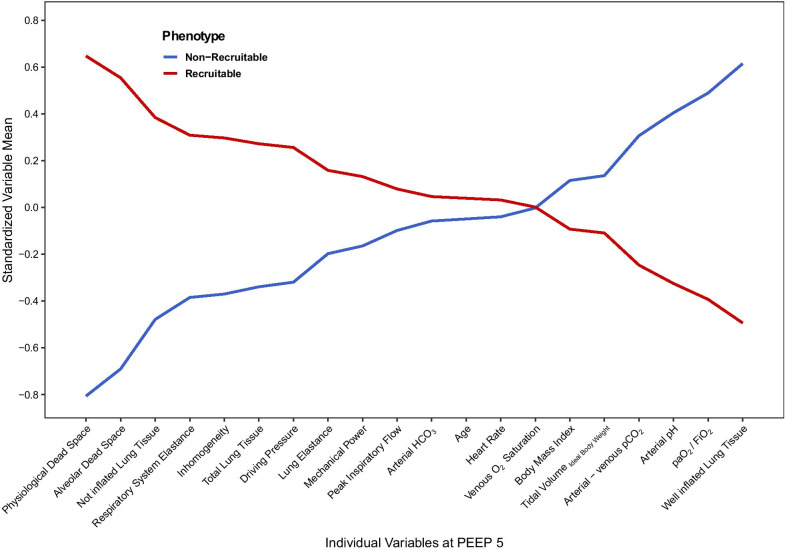
Profile plot for LCA identified, phenotype defining, continuous variables. Continuous variables are standardized to a mean of 0 and a standard deviation of 1. The order of variables is defined such that the standardized variable mean, plotted on the *y* axis, is highest on the right side of the plot for phenotype 1. PEEP, positive end-expiratory pressure; paO_2_, partial pressure of arterial oxygen; FiO_2_, fraction of inspired oxygen; pCO_2_, partial pressure of carbon dioxide

In Fig. 1, the LCA phenotype defining variables at 5 cmH_2_O are shown (Additional file 1: Table S7, Figure S5, Table S8). Phenotype 1 presented a lower respiratory system elastance, dead space and total lung tissue, as well as a higher paO_2_/FiO_2_ ratio, a more physiological pH and a less inhomogeneous lung than phenotype 2 (Additional file 1: Table 7). On the other hand, vasopressor requirements, SAPS II, age as well as BMI were comparable between both phenotypes (Table 1). Most prominently, phenotype 1 presented a lower proportion of, CT inferred, potentially recruitable lung than phenotype 2, leading to the terming of phenotype 1 as *non-recruitable* and phenotype 2 as *recruitable* phenotype (Additional file 1: Table 6).

### Response to recruitment and outcome of ARDS phenotypes

**Fig. 2:**
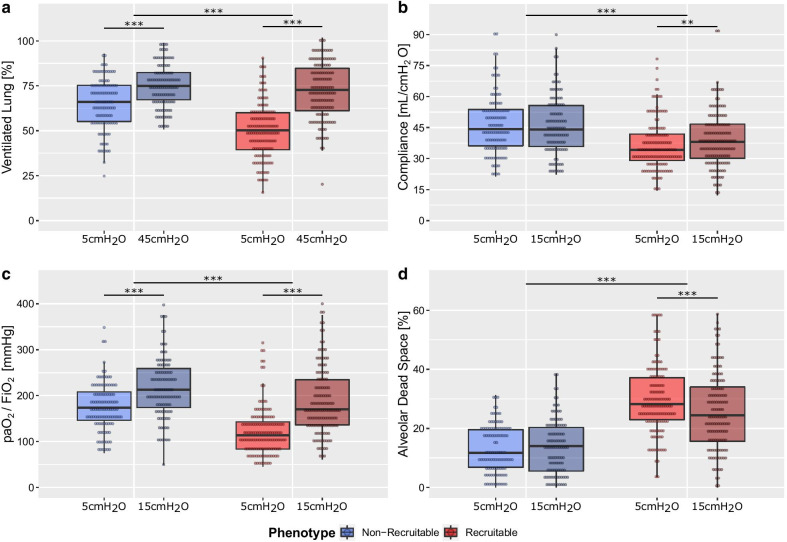
Response to recruitment manoeuvre for LCA-derived phenotypes. Box plots present **a** the amount of ventilated lung, defined as the cumulative CT-graphicly inferred poorly, well- and over-inflated lung, **b** the respiratory system compliance, **b** the paO_2_/FiO_2_ ratio and **d** the alveolar dead space at two stages of a recruitment manoeuvre for the *non-recruitable* and *recruitable* LCA phenotypes. (**a**) was measured during a inspiratory hold manoeuvre at an end-inspiratory airway pressures of 5 and 45 cmH_2_O; (**b**–**d**) were measured under positive end-expiratory pressures of 5 and 15 cmH_2_O. *p* values *< 0.05; **< 0.01; ***< 0.001 for differences in recruitment manoeuvre between phenotypes and pressures

Reflecting its designation, the *recruitable* phenotype presented an increased recruitment of ventilated lung tissue (Δ: 21 vs. 9%, *p* < 0.001) as well as an increase in compliance (Δ: 3.1 vs. 0 ml/cmH_2_O, *p* < 0.001) and paO_2_/ FiO_2_ ratio (Δ: 68 vs. 46 mmHg, *p* < 0.001), in addition to a decrease in alveolar dead space (Δ: − 3.1 vs. 1%, *p* < 0.001) in response to a standardized recruitment manoeuvre (inspiratory hold manoeuvre at 5 and 45 cmH_2_O and PEEP increase from 5 to 15 cmH_2_O) when compared to the *non-recruitable* phenotype (Fig. 2, Additional file 1: Figure S6, Table S9). The amount of potentially recruitable lung was, independently of the applied PEEP level and of the severity of ARDS at said PEEP level, systematically higher in the *recruitable* than in the *non-recruitable* phenotype (*p* < 0.001) (Additional file 1: Figure 7). Additionally, increasing ARDS severity and the amount of potentially recruitable lung were only associated in the *recruitable* phenotype (*p* < 0.001 vs. *p* = 0.59). The distributions of paO_2_/FiO_2_ ratios between both pulmonary phenotypes were highly dependent on the level of PEEP applied (Additional file 1: Figure S8).

**Fig. 3:**
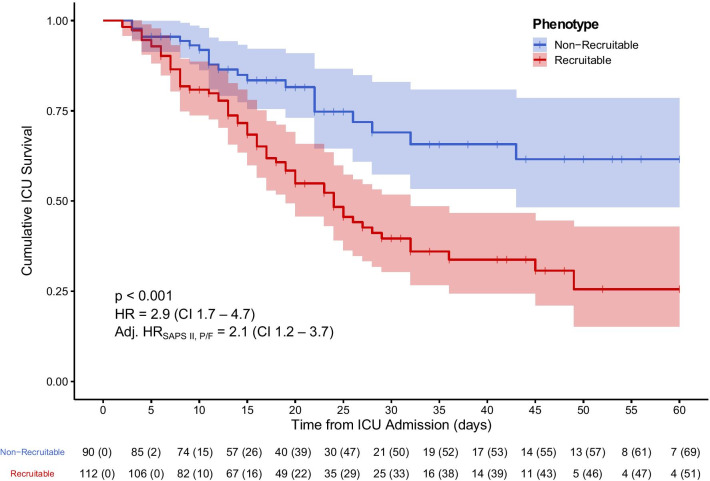
Kaplan–Meier survival curves for LCA-derived phenotypes. Kaplan–Meier curve for 60-day intensive care unit survival stratified by latent class analysis (LCA)-derived phenotype. *Non-recruitable* and *recruitable* phenotypes are plotted in blue and red colours, respectively, shaded areas represent the 95% Confidence Interval (CI). The computed hazard ratio (HR) assesses the *recruitable* using the non-*recruitable* phenotype as reference, 95% CI is given in parentheses. HRs are modelled by means of a Fine and Gray competing risk analysis. Crude and adjusted HR for SAPS II and the paO_2_/FiO_2_ ratio at a PEEP level of 5 cmH_2_O are presented. Censoring reflects patients having left the ICU alive. The underlying table presents the patients at risk per time point with the number of censored patients given in parentheses

Intensive care mortality rate, but not length of mechanical ventilation or ICU length of stay, was higher in the *recruitable* compared to the *non-recruitable* phenotype as evidenced by a crude HR of 2.9 with a 95% CI of 1.7 to 4.7 (*p* = 0.001) (Fig. 3). Adjustment for SAPS II and *P*/*F* ratio at a PEEP level of 5cmH_2_O did not influence this association. Additionally, to reject the hypothesis that the increased mortality in the *recruitable* phenotype was mainly influenced by a higher severity of ARDS, a sub-group analysis was performed including only those patients with a moderate severity of ARDS (paO_2_/FiO_2_ ratio 100–200 mmHg) at PEEP 5cmH_2_O. In this subgroup analysis, the crude and adjusted association between the *recruitable* phenotype and mortality remained patent (Additional file 1: Figure 9).

### Simplified phenotype identification

The combined LASSO and nested GLM identified two subsets of variables at a PEEP level of 5 cmH_2_O, differing on the inclusion of CT-derived parameters, as highly explanatory for phenotype identification (Additional file 1: Figure S10, Tables S10–S14). The first subset was composed of dead space, respiratory system elastance and the paO_2_/ FiO_2_ ratio (Additional file 1: Tables S11–S12). The second subset included dead space, respiratory system elastance, lung inhomogeneity and the proportion of non-aerated lung tissue (Additional file 1: Tables S13–S14). Both subsets presented outstanding AUROCs (between 0.94 [CI 95% 0.91–0.96] and 0.99 [CI 95% 0.98–0.995]) for the recognition of the *recruitable* phenotype; the use of alveolar instead of physiological dead space did not greatly impair the prognostic capacity of the models (Additional file 1: Figure S10, Table S10). In comparison, the use of SAPS II or the paO_2_/FiO_2_ ratio alone only presented poor to moderate AUROCs for the identification of the *recruitable* phenotype.

## Discussion

The present study identified two distinct ARDS phenotypes with diverging responses to a standardized recruitment manoeuvre and intensive care outcomes by means of LCA. In contrast to other published LCA analyses, only respiratory mechanics, gas-exchange and CT-derived gas- and tissue-volumes at a PEEP of 5 cmH_2_O were employed for this analysis. In order to simplify pulmonary phenotype identification, a small subset of variables with high explanatory potential was described.

The heterogeneity of the Berlin definition and the disappointing number of randomized controlled trials having attempted to propose pharmacological interventions and ventilator strategies to improve outcome in ARDS have led to a plethora of attempts to identify homogeneous subgroups of this syndrome. In order to minimize heterogeneity, identification of different ARDS subgroups by means of severity of hypoxemia [13], pulmonary or extrapulmonary origin [8], focal and non-focal pulmonary consolidations [37, 38], the fraction of dead space [49] and response to PEEP [10, 39] among others have been proposed. Strikingly, the two pulmonary phenotypes described by the LCA model in the present study contain many of these previous ARDS subclassifying characteristics. As such, phenotype 1 was characterized by a lower severity of hypoxemia at clinical PEEP, a lower proportion of ARDS of pulmonary origin, a lower fraction of dead space and a less inhomogeneous lung than phenotype 2. Indeed, the main describing feature of these two phenotypes was their response to a standardized recruitment manoeuvre, therefore leading to the designation of phenotype 1 as *non-recruitable* and phenotype 2 as *recruitable* phenotype.

The notion that patients with a potentially highly recruitable lung are at a higher risk of mortality is not precisely novel [10]. Nonetheless, to this moment, it has generally been regarded as a direct correlate to the severity of ARDS as described by the paO_2_/FiO_2_ ratio [10, 22, 40]. In the present study nevertheless, the association between higher proportions of potentially recruitable lung and increasing ARDS severity were only patent in the *recruitable* phenotype, indicating a more complex relationship than assumed up until now. Furthermore, the increased mortality in the *recruitable* phenotype was not primarily precipitated by the increased proportion of lower paO_2_/FiO_2_ ratios, remaining after statistical correction for the paO_2_/FiO_2_ ratio and in a subanalysis considering only those patients with moderate ARDS severity. Overall, the two pulmonary phenotypes here presented, congruently contained many previously described risk factors of ARDS, but could not be solely explained by the presence of one characteristic, thus suggesting the existence of two complex and distinct pulmonary entities.

Recent trials having enriched their patient recruitment by selecting patients with paO_2_/ FiO_2_ ratios below 150–200 mmHg have been able to prove the positive effect of clinical interventions, which were disappointing in previous more heterogeneous trials [15, 16, 39, 41]. It can be argued that prognostic enrichment, by severity of ARDS, was the main factor responsible for the success of these trials. Indeed, a paO_2_/FiO_2_ ratio < 150 mmHg concomitant to a PEEP ≥ 10 cmH_2_O has been independently associated with mortalities in the range of 60% [42]. Nonetheless, most of these trials targeted recruitment interventions, such as prone positioning or an increased PEEP [16, 39], and by recruiting severer patients might have been predictively enriching their studies with a high proportion of patients belonging to the *recruitable* phenotype, which, as opposed to the *non-recruitable* phenotype, presented a more prominent physiological response to recruitment and higher PEEP.

The *recruitable* phenotype was not only characterized by an impaired oxygenation, but also by a concomitantly reduced ventilation capacity. The higher proportion of physiological dead space coincident with a low respiratory system compliance probably characterize the high proportion of inhomogeneously ventilated, mainly collapsed and potentially recruitable lung in the *recruitable* phenotype [10]. A recent trial assessing the use of personalized mechanical ventilation, including recruitment manoeuvres and higher PEEP settings, in non-focal, inhomogeneous, as opposed to focal ARDS, suggested a survival benefit [43]. Most importantly, misclassification of lung morphology had a large effect on mortality. If a personalized ventilation approach would have led to reduced mortalities in the *recruitable* phenotype remains hypothetical; nonetheless, clinical PEEP levels employed in the *recruitable* phenotype were lower than personalized approaches would have targeted [43–45] and mortality was as high as in the misclassified lung morphologies in the LIVE study [43].

The pulmonary phenotypes in the present study differ in their inception from the *hypo-* and *hyperinflammatory* phenotypes proposed in the seminal study by Calfee et al., as no inflammatory or laboratory parameters were available for the LCA analysis. Indeed, the two pulmonary phenotypes identified in this study differ from the inflammatory phenotypes in multiple aspects. The *recruitable* and *non-recruitable* phenotype had similar vasopressor requirements at admission, one of the main clinical features differentiating the two inflammatory phenotypes. Furthermore, the phenotypes described by Calfee et al. present similar paO_2_/FiO_2_ ratios and differ in severity scoring, much opposed to the here presented pulmonary phenotypes. Nonetheless, other features of both phenotype descriptions overlap; as such, the *recruitable* and *hyperinflammatory* phenotype both present lower respiratory system compliances and a more pronounced acidosis. The lack of biological data in this study prevents identification of a direct correlation between the pulmonary and inflammatory phenotypes. Nonetheless, multiple studies have independently shown associations between high recruitability, pulmonary inhomogeneity, predominance of a primary ARDS, all characteristics of the *recruitable* phenotype and the presence of increased pulmonary inflammatory biomarkers such as sRAGE [46–48] which have been linked to the *hyperinflammatory* phenotype [20, 49]. A certain overlap between the *recruitable* and the *hyperinflammatory* phenotype would also explain the positive response to PEEP in the *hyperinflammatory* phenotype [19].

Differences and overlaps between phenotypes are not surprising; indeed, the description of the *hypo-* and *hyperinflammatory* phenotype does not preclude the existence of further phenotypes in ARDS. As in many other diseases and syndromes, a plethora of different phenotypes, overlapping in multiple facets and with clear-cut differentiation in others, might very well exist [50]. Identification of the pulmonary and inflammatory phenotypes may thus be complementary, while enrichment of immunomodulatory trials could profit from phenotypisation by inflammatory phenotypes, trials targeting personalized mechanical ventilation and recruitment strategies might benefit from enrichment by pulmonary phenotypes [51]. This admittedly complex customization of trials might be the key to success in personalized ARDS medicine, in analogy to the great variance of phenotype-enriched trials in oncology [52].

The present study has to account for certain limitations. First and foremost, this study is a retrospective analysis of a prospective cohort with all the limitations a post hoc analysis may encompass to the generalizability of the discussed results. Nonetheless, multiple sensitivity analyses suggest internal robustness of the LCA model and the inferred pulmonary phenotypes. Second, due to the extended inclusion period of 16 years, the moderate inclusion rate of one patient per month and the ARDS criteria changing in 2012, the possibility cannot be ruled out, that clinical diagnosis of ARDS was missed and a reduced number of patients were not included in the present cohort. However, as the characteristics of the described ARDS population are comparable to other cohorts and the pulmonary phenotyping was indifferent to temporality in the sensitivity analyses, selection bias can be regarded as residual. Third, the follow-up of the patients was limited to ICU outcome status and no data regarding hospital mortality were available. To mitigate the resulting presence of right informative censoring, Fine and Gray competing risk modelling was employed. Fourth, the presence of a moderate proportion of missing values, albeit mitigated by use of a multiple imputation methodology, might have influenced the final LCA phenotype description. Fifth, no biomarkers were collected in the framework of this study, precluding comparison of the here proposed pulmonary phenotypes with the inflammatory phenotypes and preventing the investigation of a deeper biological association between the phenotypes. Sixth, time from ICU admission to CT-scans and respiratory mechanics assessment was variable between patients, as such, temporal influence on the LCA results cannot be ruled out. Likewise, the stability of the pulmonary phenotypes over time has not been assessed. Seventh, no information on longitudinally employed ventilation settings was available, preventing stratified analysis of the effect of these settings on mortality in the different phenotypes. Finally, this study and the here described phenotypes lack external validation in an independent cohort.

## Conclusions

In conclusion, the present study identifies two ARDS phenotypes based on respiratory mechanics, gas-exchange and CT-derived gas- and tissue-volumes. These phenotypes are characterized by distinctly diverse responses to a standardized recruitment manoeuvre and by a diverging mortality. Given multicentre validation, the simple and rapid identification of these pulmonary phenotypes could facilitate enrichment of future prospective clinical trials addressing mechanical ventilation strategies in ARDS.

## Supplementary Information

**Additional file ****1****: Appendix 1.** Additional Information. **Table S1.** Variable Missingness. **Figure S1.** Multiple Imputation—Convergence plots. **Figure S2.** Multiple Imputation—Distribution plots. **Table S2.** Internal validity analysis of LCA class assignment. **Figure S3.** Internal validity analysis of LCA class assignment—Kaplan–Meier. **Table S3.** Imputation model-dependent latent class transitions and outcome data. **Figure S4.** Imputation model-dependent Kaplan–Meier curves. **Appendix 2.** Complete case sensitivity analysis. **Table S4.** Respiratory mechanics—gas exchange and computed tomography data at 5 cmH_2_O of PEEP. **Table S5.** Respiratory mechanics and gas exchange at 15 cmH_2_O of PEEP. **Table S6.** Computed tomography data at 45 cmH_2_O of PEEP. **Table S7.** Latent class analysis identified, phenotype defining variables at PEEP 5. **Figure S5.** Profile plot all variables at PEEP 5. **Table S8.** All variables at PEEP 5 cmH_2_O employed for the LCA. **Figure S6.** Response to recruitment manoeuvre for LCA-derived phenotypes (with trendlines). **Table S9.** Full specification of mixed-effect models. **Figure S7.** Dependency of the amount of potentially recruitable lung on the PaO_2_/ FiO_2_ ratio depending on the pulmonary phenotype. **Figure S8.** Distribution of PaO_2_/ FiO_2_ Ratios in pulmonary phenotypes stratified by underlying PEEP. **Figure S9.** Survival analysis for patients with moderate ARDS. **Figure S10.** Receiver operating characteristics curves of phenotype prediction model. **Table S10.** Area under the receiver operating curves (AUROCs) for the LASSO and nested GLM inferred models and classic severity scores. **Table S11.** LASSO + Nested Generalized Logistic Regression for “Elastance_Respiratory System_, Dead Space_Physiological_ and *P*/*F* ratio” at PEEP 5. **Table S12.** LASSO + Nested Generalized Logistic Regression for “Elastance_Respiratory System_, Dead Space_Alveolar_ and *P*/*F* ratio” at PEEP 5. **Table S13.** LASSO + Nested Generalized Logistic Regression for “Elastance_Respiratory System_, Dead Space_Physiological_ and *P*/*F* ratio” at PEEP 5ss. **Table S14.** LASSO + Nested Generalized Logistic Regression for “Elastance_Respiratory System_, Dead Space_Alveolar_ and *P*/*F* ratio” at PEEP 5.

## Data Availability

All data analysed and discussed in the framework of this study are included in this published article and its online supplementary information. The corresponding author may provide specified analyses or fully de-identified parts of the dataset upon reasonable request.

## Abbreviations

ARDS: Acute respiratory distress syndrome
AIC: Akaike information criterion
AUC: Area under the curve
BIC: Bayesian information criterion
BLRT: Bootstrap likelihood ratio test
CT: Computed tomography
GLM: General linear regression model
FiO_2_: Inspiratory fraction of oxygen
ICU: Intensive care unit
LCA: Latent class analysis
LASSO: Least absolute shrinkage and selection operator
PEEP: Positive end expiratory pressure
ROC: Receiver operating characteristics
SAPS II: Simplified Acute Physiology Score II
SMD: Standard mean differences
