# Supplementary material for: Latent class analysis to predict intensive care outcomes in Acute Respiratory Distress Syndrome: a proposal of two pulmonary phenotypes
Source: Crit Care. 2021 Apr 22;25:154. doi: 10.1186/s13054-021-03578-6 (PMC8060783; doi:10.1186/s13054-021-03578-6)
Supplement: Supplementary file 1 — Additional file 1: Appendix 1. Additional Information. Table S1. Variable Missingness. Figure S1. Multiple Imputation—Convergence plots. Figure S2. Multiple Imputation—Distribution plots. Table S2. Internal validity analysis of LCA class assignment. Figure S3. Internal validity analysis of LCA class assignment—Kaplan–Meier. Table S3. Imputation model-dependent latent class transitions and outcome data. Figure S4. Imputation model-dependent Kaplan–Meier curves. Appendix 2. Complete case sensitivity analysis. Table S4. Respiratory mechanics—gas exchange and computed tomography data at 5 cmH2O of PEEP. Table S5. Respiratory mechanics and gas exchange at 15 cmH2O of PEEP. Table S6. Computed tomography data at 45 cmH2O of PEEP. Table S7. Latent class analysis identified, phenotype defining variables at PEEP 5. Figure S5. Profile plot all variables at PEEP 5. Table S8. All variables at PEEP 5 cmH2O employed for the LCA. Figure S6. Response to recruitment manoeuvre for LCA-derived phenotypes (with trendlines). Table S9. Full specification of mixed-effect models. Figure S7. Dependency of the amount of potentially recruitable lung on the PaO2/ FiO2 ratio depending on the pulmonary phenotype. Figure S8. Distribution of PaO2/ FiO2 Ratios in pulmonary phenotypes stratified by underlying PEEP. Figure S9. Survival analysis for patients with moderate ARDS. Figure S10. Receiver operating characteristics curves of phenotype prediction model. Table S10. Area under the receiver operating curves (AUROCs) for the LASSO and nested GLM inferred models and classic severity scores. Table S11. LASSO + Nested Generalized Logistic Regression for “ElastanceRespiratory System, Dead SpacePhysiological and P/F ratio” at PEEP 5. Table S12. LASSO + Nested Generalized Logistic Regression for “ElastanceRespiratory System, Dead SpaceAlveolar and P/F ratio” at PEEP 5. Table S13. LASSO + Nested Generalized Logistic Regression for “ElastanceRespiratory System, Dead SpacePhysiological and P/F ratio” at PEE [file 13054_2021_3578_MOESM1_ESM.docx]

**Online Supplementary Information**

**Latent class analysis to predict intensive care outcomes in ARDS: a proposal of two pulmonary phenotypes**
*Pedro D. Wendel Garcia, Alessio Caccioppola, Silvia Coppola, Tommaso Pozzi, Arianna Ciabattoni, Stefano Cenci, Davide Chiumello*

- **e-Appendix 1:** Additional Information
- **e-Table 1:** Variable Missingness
- **e-Figure 1:** Multiple Imputation - Convergence plots
- **e-Figure 2:** Multiple Imputation – Distribution plots
- **e-Table 2:** Internal validity analysis of LCA class assignment
- **e-Figure 3:** Internal validity analysis of LCA class assignment – Kaplan Meier
- **e-Table 3:** Imputation model dependent latent class transitions and outcome data
- **e-Figure 4:** Imputation model dependent Kaplan Meier Curves
- **e-Appendix 2:** Complete case sensitivity analysis
- **e-Table 4:** Respiratory mechanics - gas exchange and computed tomography data at 5 cmH_2_O of PEEP
- **e-Table 5:** Respiratory mechanics and gas exchange at 15 cmH_2_O of PEEP
- **e-Table 6:** Computed tomography data at 45 cmH_2_O of PEEP
- **e-Table 7:** Latent Class Analysis identified, phenotype defining variables at PEEP 5
- **e-Figure 5:** Profile Plot All Variables at PEEP 5
- **e-Table 8:** All Variables at PEEP 5 cmH_2_O employed for the LCA
- **e-Figure 6:** Response to recruitment manoeuvre for LCA derived phenotypes (with trendlines)
- **e-Table 9:** Full specification of mixed-effect models
- **e-Figure 7:** Dependency of the amount of potentially recruitable lung on the PaO_2_/ FiO_2_ ratio depending on the pulmonary phenotype
- **e-Figure 8:** Distribution of PaO_2_/ FiO_2_ Ratios in pulmonary phenotypes stratified by underlying PEEP
- **e-Figure 9:** Survival Analysis for Patients with Moderate ARDS
- **e-Figure 10:** Receiver Operating Characteristics Curves of Phenotype Prediction Model
- **e-Table 10:** Area Under the Receiver Operating Curves (AUROCs) for the LASSO and nested GLM inferred models and classic severity scores.
- **e-Table 11:** LASSO + Nested Generalized Logistic Regression for “Elastance_Respiratory System_, Dead Space_Physiological_ and P/F ratio” at PEEP 5
- **e-Table 12:** LASSO + Nested Generalized Logistic Regression for “Elastance_Respiratory System_, Dead Space_Alveolar_ and P/F ratio” at PEEP 5
- **e-Table 13:** LASSO + Nested Generalized Logistic Regression for “Elastance_Respiratory System_, Dead Space_Physiological_ and P/F ratio” at PEEP 5ss
- **e-Table 14:** LASSO + Nested Generalized Logistic Regression for “Elastance_Respiratory System_, Dead Space_Alveolar_ and P/F ratio” at PEEP 5

**e-Appendix 1. Additional Information**

*Mechanical ventilation setting and measurement of respiratory mechanics*

To standardize the lung volume history, a recruitment manoeuvre was performed in pressure-controlled ventilation at a PEEP of 5 cmH_2_O, with a plateau pressure of 45 cmH_2_O for 2 min. 20 min after the recruitment manoeuvre, all respiratory mechanics measurements and blood gas analyses were performed with a PEEP of 5 cmH*_2_*O while the remaining settings were maintained unchanged from baseline. The mean-expired partial pressure of carbon dioxide was measured with the CO2SMO monitor (Novametrix, Wallingford, CT). Patients were treated in volume controlled mechanical ventilation. Plateau pressure was measured using a 5 second hold at end-inspiration and end-expiratory pressure using a 5 second hold at end-expiration. Briefly, the respiratory flow rate was measured with a heated pneumotachograph (Fleisch n°2, Fleisch, Lausanne, Switzerland). Airway pressure was measured proximally to the endotracheal tube with a dedicated pressure transducer (MPX 2010 DP. Motorola, Solna, Sweden). Esophageal pressure was measured with a radio-opaque balloon (SmartCath Bicore, USA), positioned in the lower third of the esophagus, inflated with 1.0–1.5 mL of air and connected to a pressure transducer (MPX 2010 DP. Motorola, Solna, Sweden). All traces were sampled at 100 Hz and processed on a dedicated data acquisition system (Colligo and Computo). Data presented are the ones measured with the calibrated acquisition system and not the ones set on the ventilator (set and measured values could differ by up to 10%).

*CT scan variables*

After stabilization, patients were directly moved to the radiology department and a whole-lung CT scan was performed at an inspiratory-plateau pressure of 45 cm of water during an end-inspiratory pause (ranging from 15 to 25 seconds) and thereafter at PEEP values of 5 and 15 cm of water applied in a random order during an end-expiratory pause (ranging from 15 to 25 seconds). Immediately before each CT scan was obtained, a recruitment manoeuvre was performed. The ventilator settings were otherwise kept identical to those used during the PEEP trial. The cross-sectional lung images were processed and analysed by a custom-designed software package. Briefly, the outline of the lungs was manually drawn in each image, excluding the hilar vessels and the main bronchi, and gas and tissue volumes were determined voxel-by-voxel and each voxel was classified according to its gas/tissue content. Specific lung weight was assumed to be equal to 1, and the total lung weight was calculated from the physical density of the lung expressed in Hounsfield units. Similarly, the tissue weights of lung regions with different degrees of aeration were calculated. The regions were classified as non-aerated (density between +100 and –100 Hounsfield units), poorly aerated (density between –101 and –500 Hounsfield units), well-aerated (density between –501 and –900 Hounsfield units), and hyperinflated (density between –901 and –1000 Hounsfield units). The percentage of potentially recruitable lung was defined as the proportion of the total lung weight accounted for by nonaerated lung tissue in which aeration was restored (according to CT) by an airway pressure of 45 cmH_2_O, which we arbitrarily assumed to be the “full recruitment”, from an airway pressure of 5 cmH_2_O. The lung inhomogeneity was measured by comparing the inflation of neighbouring lung regions: if two neighbouring regions were perfectly “homogeneous” at a given pressure, their inflation should be similar and the inflation ratio of the two regions is defined as one. The lung inhomogeneity threshold was defined as the percentage of lung volume presenting an inflation ratio greater than 1.61 (95th percentile of a control population).

*Calculation of respiratory mechanics and physiological variables*

$$Alveolar Dead Space= \frac{arterial p{CO}_{2}-End-Tidal{CO}_{2}}{arterial p{CO}_{2}}$$

$$Physiological Dead Space= \frac{{arterial pCO}_{2}-p_{mixed-expiratory}{CO}_{2}}{p_{arterial}{CO}_{2}}$$

$$Anatomical Dead Space=Physiological Dead Space-Alveolar Dead Space$$

$$Driving Pressure=End-inspiratory Airway Pressure - End-expiratory Airway Pressure$$

$$Airway Resistance=\frac{Peak Airway Pressure-End-inspiratory Airway Pressure}{Inspiratory Flow}$$

$$Respiratory System Elastance=\frac{End-inspiratory Airway Pressure-End-expiratory Airway Pressure}{Tidal Volume}$$

$$Chest Wall Elastance=\frac{End-inspiratory Esophageal Pressure-End-expiratory Esophageal Pressure}{Tidal Volume}$$

$$Lung Elastance = Respiratory System Elastance-Chest Wall Elastance$$

$$Elastance derived Transpulmonary pressure=End-inspiratory Airway Pressure \times\frac{Lung Elastance}{Respiratory System Elastance}$$

$${Mechanical Power}_{Static Component}=0.098\times Respiratory Rate\times Tidal Volume\times PEEP$$

$${Mechanical Power}_{Dynamic Component}=0.098\times Respiratory Rate\times Tidal Volume\times\left( \frac{1}{2}\times Tidal Volume\times Respiratory System Elastance \right)$$

$${Mechanical Power}_{Resistive Component}=0.098\times Respiratory Rate\times Tidal Volume\times\left( Inspiratory Flow\times Airway Resistance \right)$$

$$Mechanical Power=0,098\times Respiratory Rate\times Tidal Volume\times\left( Peak Airway Pressure-\left( Driving Pressure\times\frac{1}{2} \right) \right)$$

$$Content {CO}_{2}={HCO}_{3}+\left( {pCO}_{2}\times0.03 \right)$$

$$Content O_{2}=\left( 0.003\times{pO}_{2} \right)+(1.34\times Saturation O_{2}\times Hb)$$

$$Oxygenation Index = \frac{FiO_{2}\times Mean Airway Pressure}{paO_{2}}$$

**e-Table 1. Variable Missingness**

| **Variable** | **Missing** | **Variable** | **Missing** | **Variable** | **Missing** | **Variable** | **Missing** |
| --- | --- | --- | --- | --- | --- | --- | --- |
| Mean-expired pCO_2 PEEP 15_ | 42.08% | Total lung gas _PEEP 45_ | 9.72% | Tidal Volume _PEEP 15_ | 3.24% | Arterial pCO_2_ _PEEP 15_ | 1.21% |
| Mean-expired pCO_2 PEEP 5_ | 40.83% | Not inflated lung tissue _PEEP 45_ | 9.72% | Peak Inspiratory Flow _PEEP 15_ | 3.24% | Arterial pO_2_ _PEEP 15_ | 1.21% |
| End-tidal pCO_2 PEEP 15_ | 43.72% | Poorly inflated lung tissue _PEEP 45_ | 9.72% | Mean Airway Pressure _PEEP 15_ | 3.24% | Arterial HCO_3_ _PEEP 15_ | 1.21% |
| End-tidal pCO_2_ _PEEP 5_ | 42.51% | Well inflated lung tissue _PEEP 45_ | 9.72% | Peak airway Pressure _PEEP 15_ | 3.24% | Arterial pCO_2_ _baseline_ | 0.81% |
| End-inspiratory esophageal pressure _PEEP 15_ | 31.58% | Over inflated lung tissue _PEEP 45_ | 9.72% | End-inspiratory airway Pressure _PEEP 15_ | 3.24% | Arterial pO_2_ _baseline_ | 0.81% |
| End-expiratory esophageal pressure _PEEP 15_ | 31.58% | Not inflated lung volume _PEEP 45_ | 9.72% | Central Venous Pressure _baseline_ | 2.83% | Respiratory Rate _PEEP 15_ | 0.81% |
| End-inspiratory esophageal pressure _PEEP 5_ | 31.17% | Poorly inflated lung volume _PEEP 45_ | 9.72% | Mean Airway Pressure _PEEP 5_ | 2.83% | Minute Ventilation _PEEP 15_ | 0.81% |
| End-expiratory esophageal pressure _PEEP 5_ | 31.17% | Well inflated lung volume _PEEP 45_ | 9.72% | Peak Pressure _PEEP 5_ | 2.83% | FiO_2_ _PEEP 15_ | 0.81% |
| Inhomogeneity | 31.17% | Over inflated lung volume _PEEP 45_ | 9.72% | End-inspiratory airway Pressure _PEEP 5_ | 2.83% | Heart Rate _PEEP 5_ | 0.81% |
| Arterial Hemoglobin _baseline_ | 29.96% | Arterial Base Excess _PEEP 15_ | 8.50% | Central Venous Pressure _PEEP 5_ | 2.43% | Tidal Volume _baseline_ | 0.40% |
| Arterial Base Excess _baseline_ | 29.55% | Arterial Base Excess _PEEP 5_ | 7.29% | Tidal Volume _PEEP 5_ | 2.43% | Respiratory Rate _baseline_ | 0.40% |
| Venous Hemoglobin _PEEP 15_ | 21.46% | Mean Airway Pressure _baseline_ | 6.88% | Inspiratory Flow _Peak – PEEP 5_ | 2.43% | FiO_2_ _baseline_ | 0.40% |
| Venous Hemoglobin _PEEP 5_ | 21.05% | Peak airway Pressure _baseline_ | 4.45% | Arterial pH _baseline_ | 2.02% | Respiratory Rate _PEEP 5_ | 0.40% |
| Venous HCO_3 PEEP 15_ | 19.84% | Central Venous Pressure _PEEP 15_ | 4.45% | O_2_ arterial saturation _baseline_ | 2.02% | Minute Ventilation _PEEP 5_ | 0.40% |
| Venous HCO_3 PEEP 5_ | 19.03% | Total lung volume _PEEP 5_ | 3.64% | Heart Rate _baseline_ | 2.02% | FiO_2_ _PEEP 5_ | 0.40% |
| Venous pCO_2_ _PEEP 15_ | 18.22% | Total lung tissue _PEEP 5_ | 3.64% | Systolic Arterial Pressure _PEEP 15_ | 2.02% | Arterial Hemoglobin _PEEP 5_ | 0.40% |
| Venous pO_2_ _PEEP 15_ | 18.22% | Total lung gas _PEEP 5_ | 3.64% | Diastolic Arterial Pressure _PEEP 15_ | 2.02% | Age | 0.00% |
| Venous O_2_ saturation _PEEP 15_ | 18.22% | Not inflated lung tissue _PEEP 5_ | 3.64% | Heart Rate _PEEP 15_ | 2.02% | Weight | 0.00% |
| Venous O_2_ saturation _PEEP 5_ | 17.41% | Poorly inflated lung tissue _PEEP 5_ | 3.64% | Systolic Arterial Pressure _baseline_ | 1.62% | Height | 0.00% |
| Venous pCO_2_ _PEEP 5_ | 17.00% | Well inflated lung tissue _PEEP 5_ | 3.64% | Diastolic Arterial Pressure _baseline_ | 1.62% | PEEP _baseline_ | 0.00% |
| Venous pO_2_ _PEEP 15_ | 17.00% | Over inflated lung tissue _PEEP 5_ | 3.64% | Arterial Hemoglobin _PEEP 15_ | 1.62% | Minute Ventilation _baseline_ | 0.00% |
| End-Tidal CO_2 PEEP 15_ | 12.96% | Not inflated lung volume _PEEP 5_ | 3.64% | Arterial O_2_ saturation _PEEP 15_ | 1.62% | Arterial pH _PEEP 5_ | 0.00% |
| End-Tidal CO_2 PEEP 5_ | 11.74% | Poorly inflated lung volume _PEEP 5_ | 3.64% | Arterial HCO_3_ _baseline_ | 1.21% | Arterial pCO_2_ _PEEP 5_ | 0.00% |
| End-inspiratory airway Pressure _baseline_ | 9.72% | Well inflated lung volume _PEEP 5_ | 3.64% | Systolic Arterial Pressure _PEEP 5_ | 1.21% | Arterial pO_2_ _PEEP 5_ | 0.00% |
| Total lung volume _PEEP 45_ | 9.72% | Over inflated lung volume _PEEP 5_ | 3.64% | Diastolic Arterial Pressure _PEEP 5_ | 1.21% | Arterial HCO_3_ _PEEP 5_ | 0.00% |
| Total lung tissue _PEEP 45_ | 9.72% | Respiratory Time _PEEP 15_ | 3.24% | Arterial pH _PEEP 15_ | 1.21% | Arterial O_2_ saturation _PEEP 5_ | 0.00% |

List of abbreviations: PEEP positive end-expiratory pressure; pCO_2_ partial pressure of carbon dioxide; pO_2_ partial pressure of oxygen; FiO_2_ inspiratory fraction of oxygen.


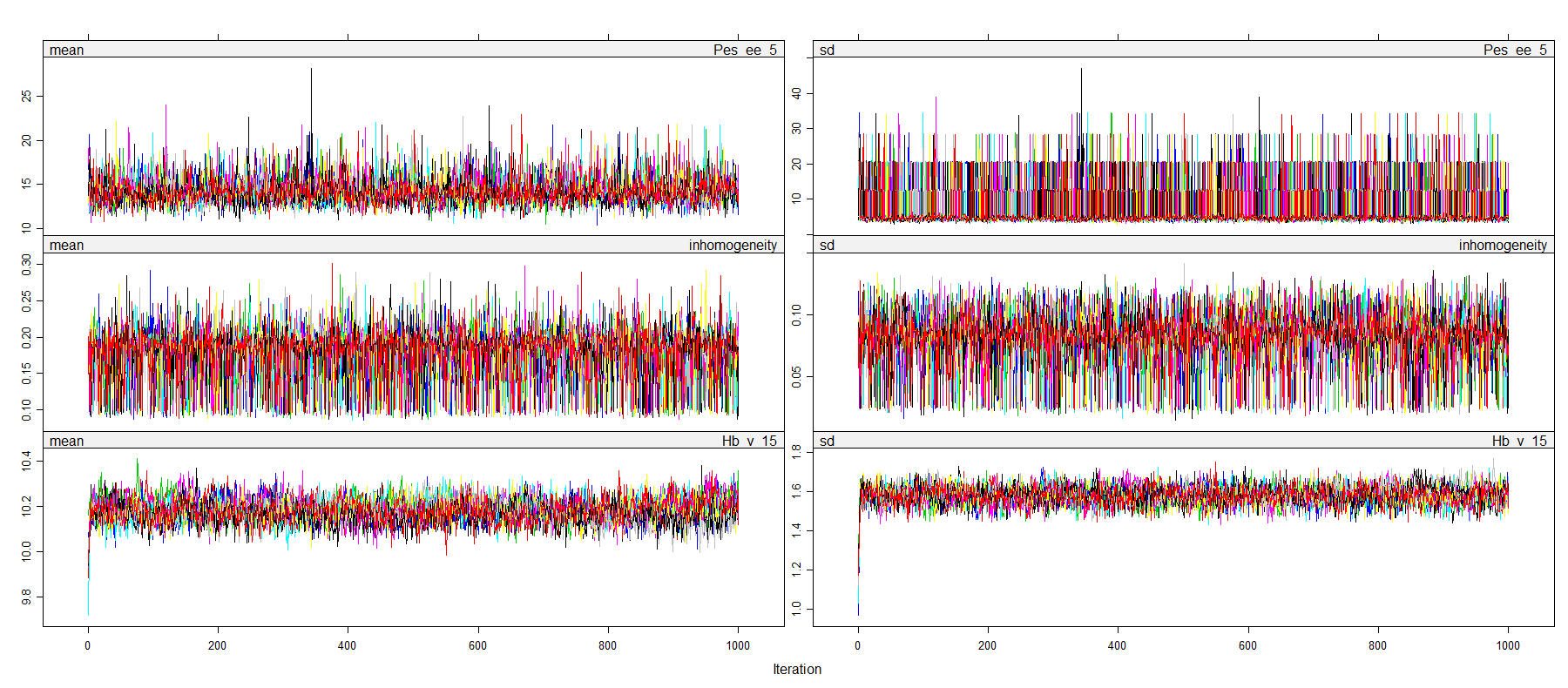
**
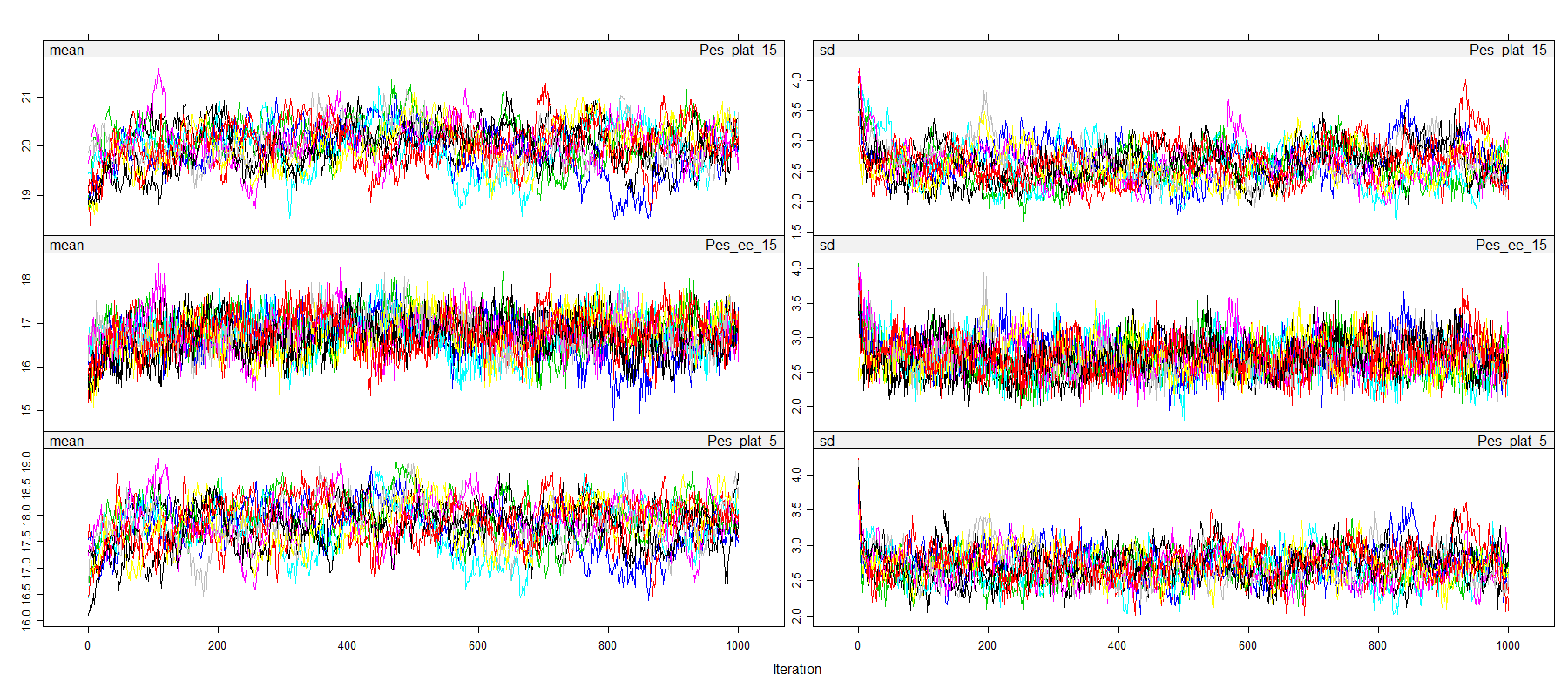
e-Figure 1. Multiple Imputation - Convergence plots**


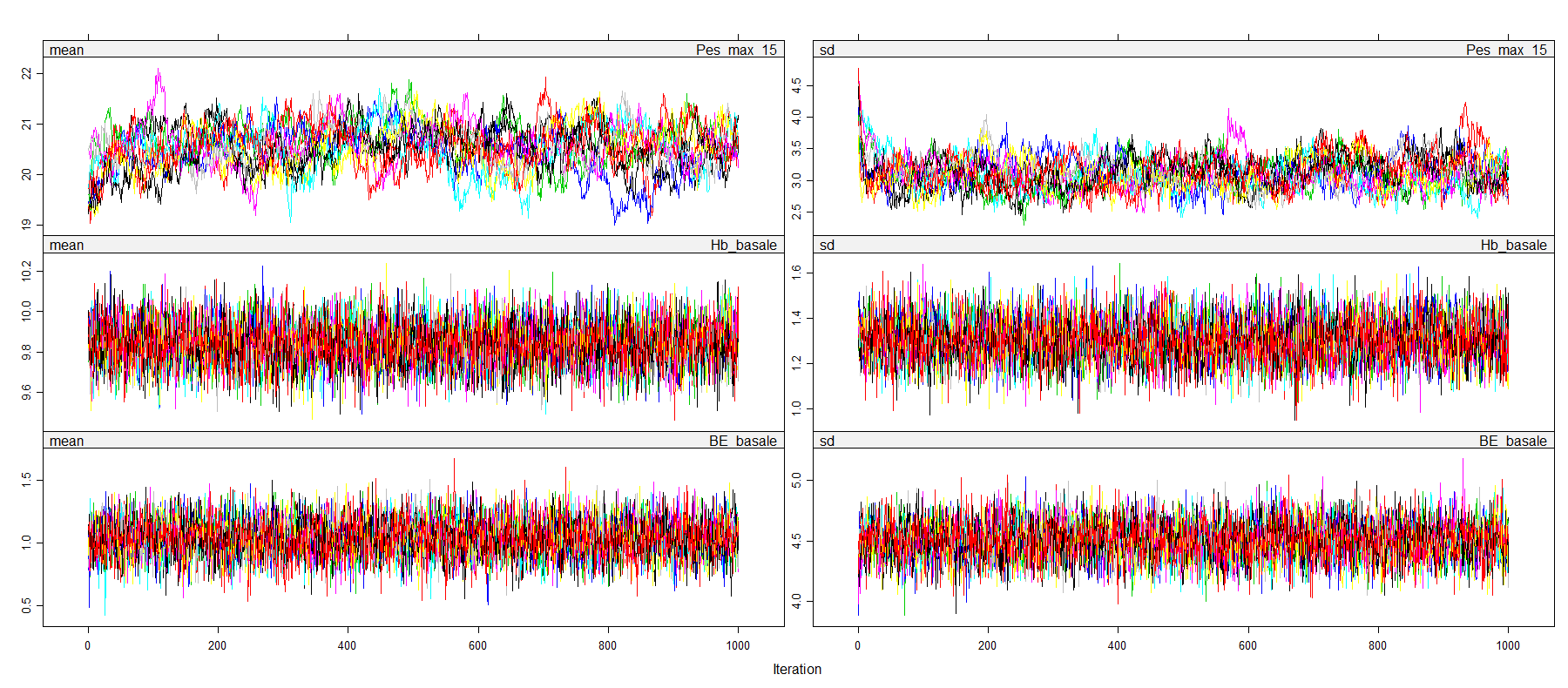

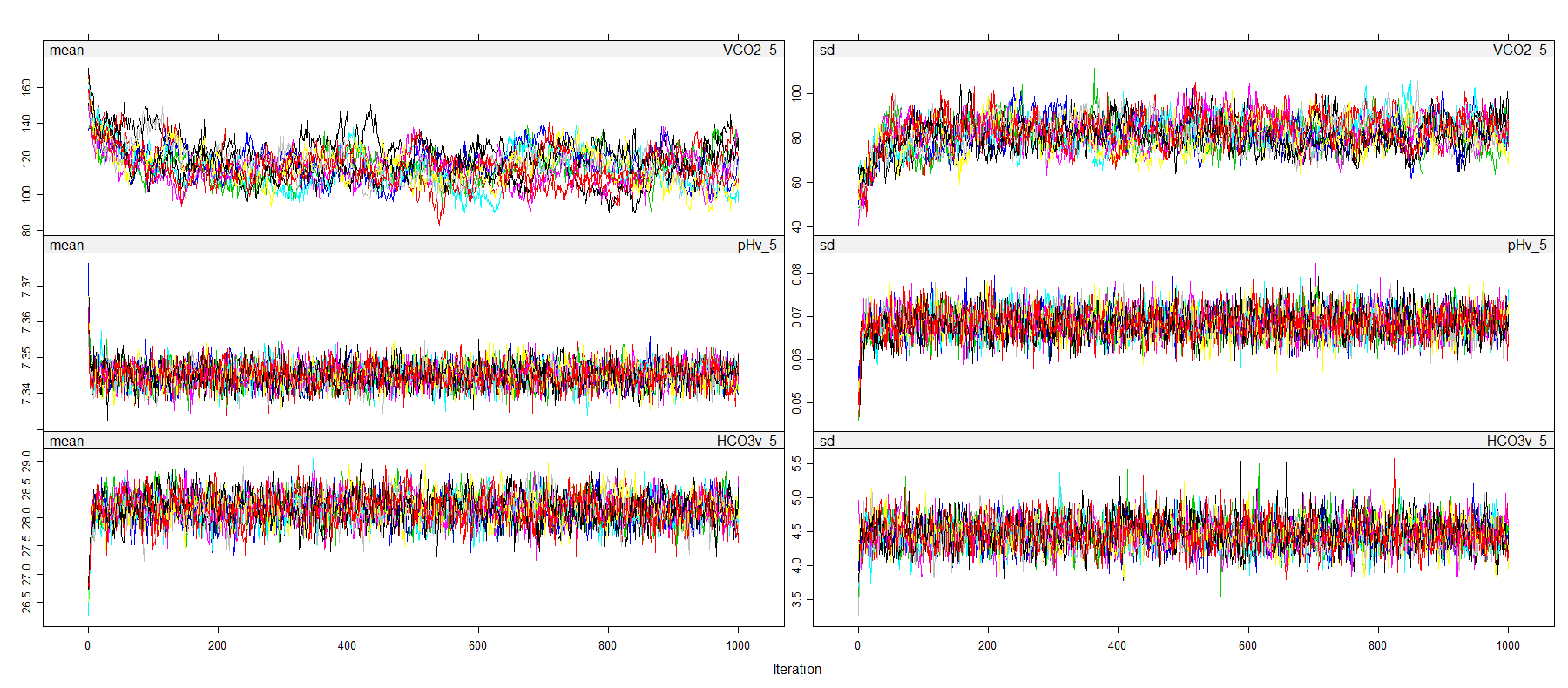

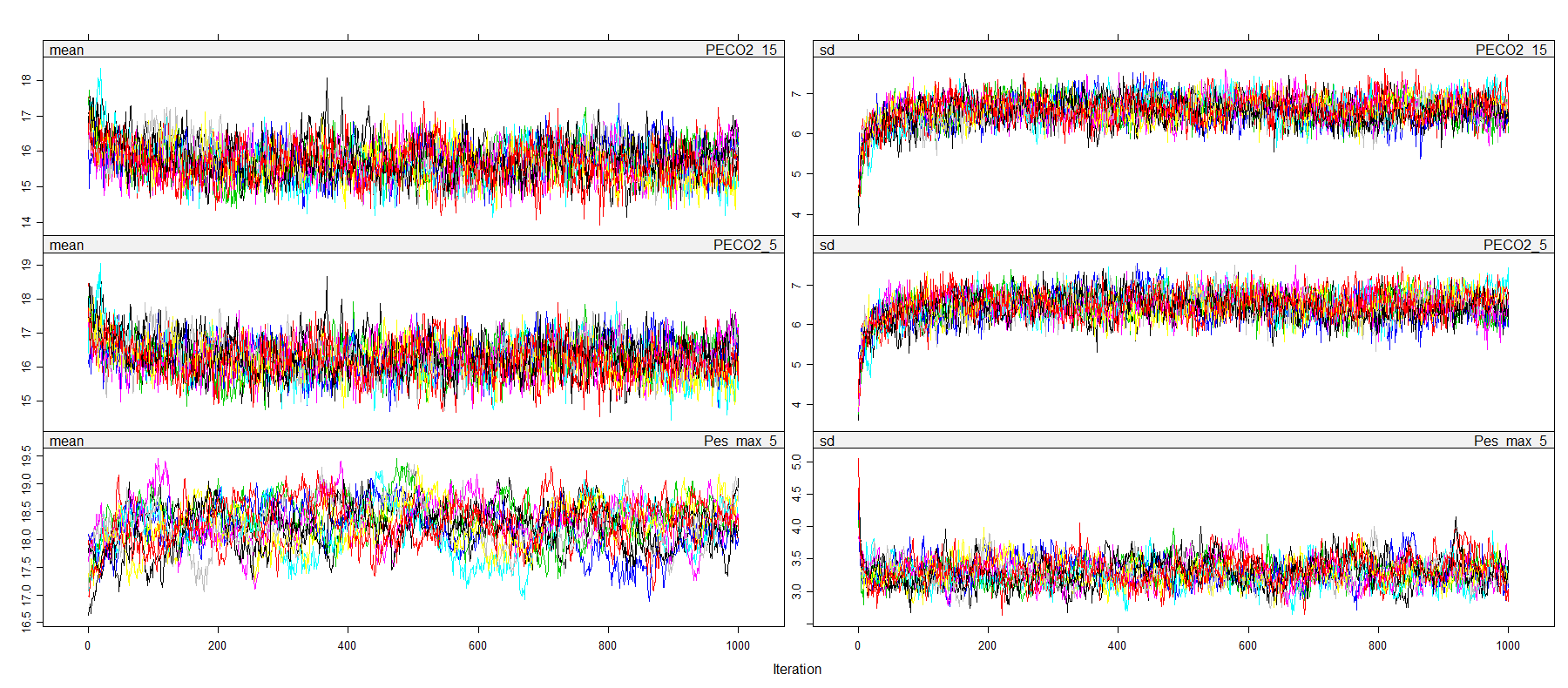

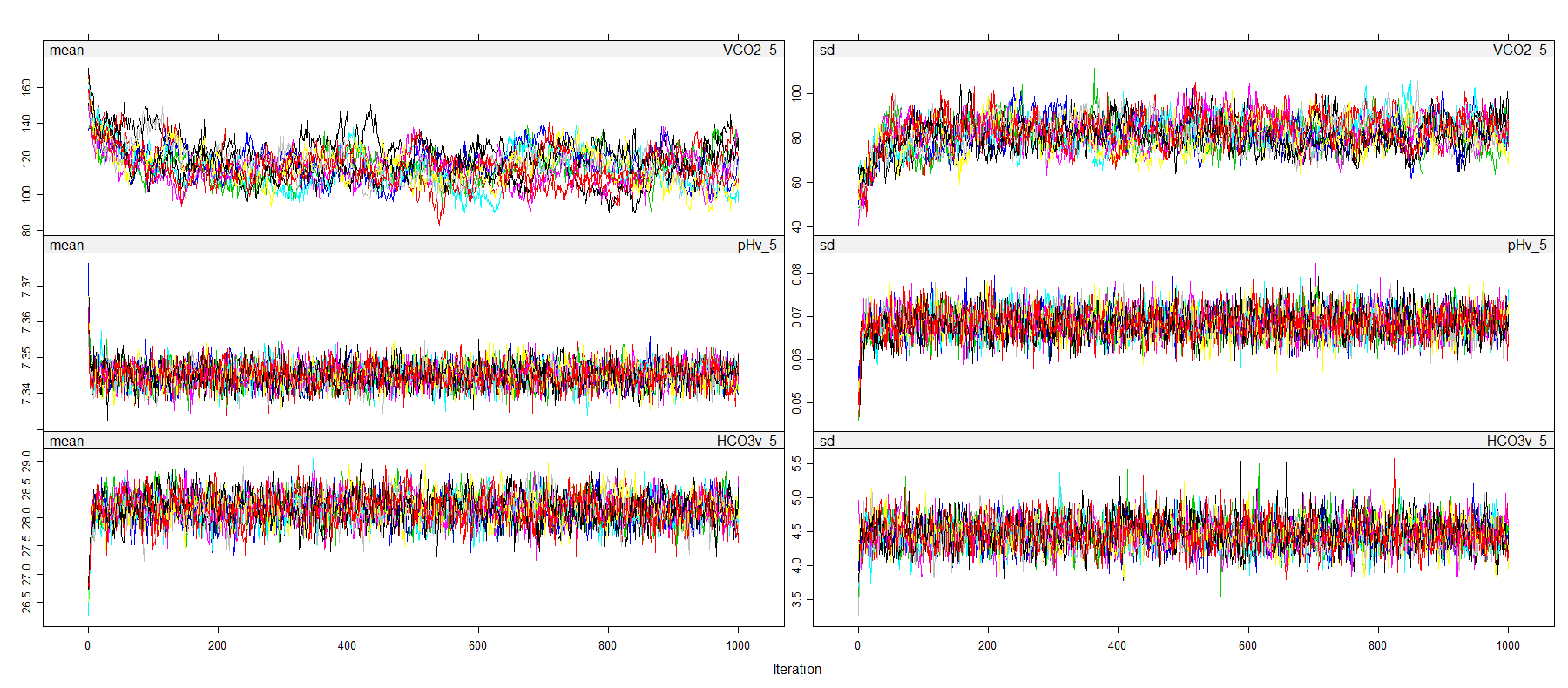


**
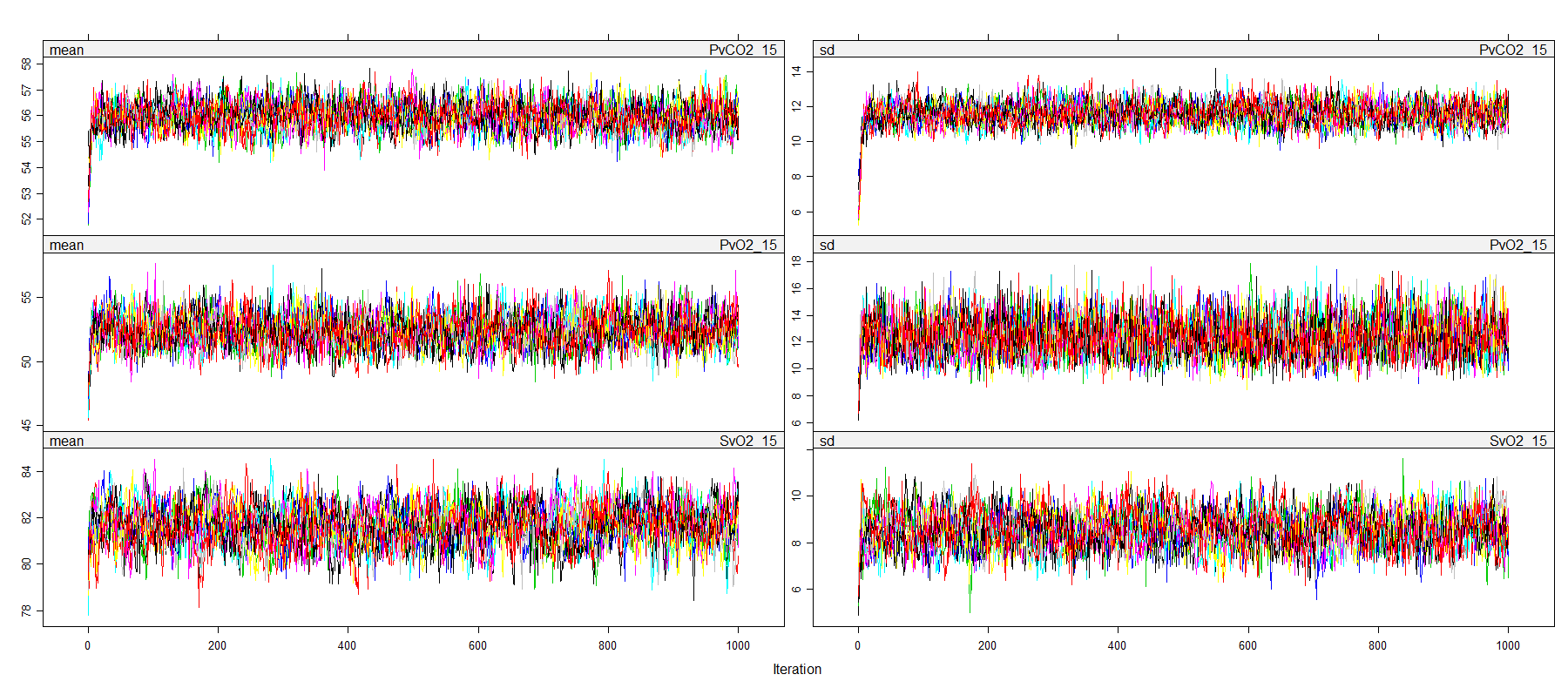

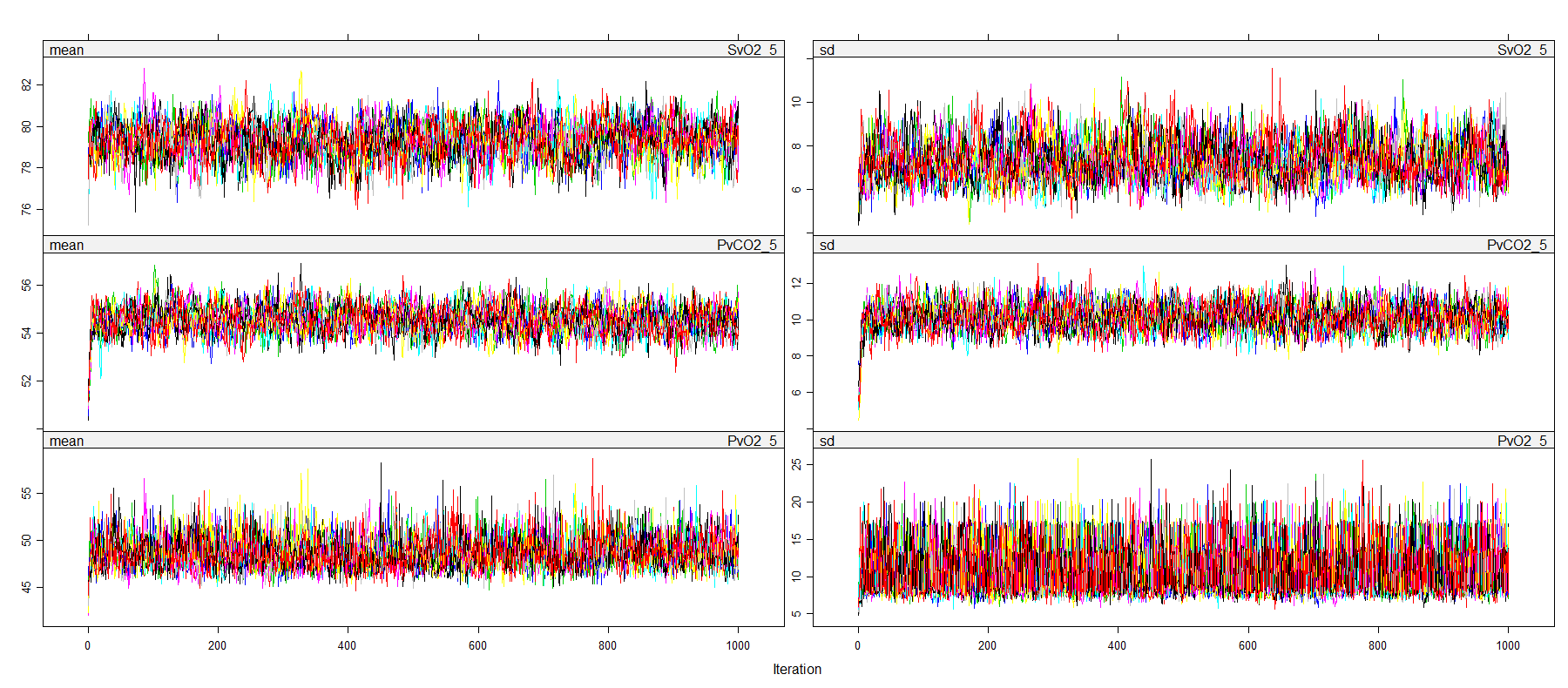

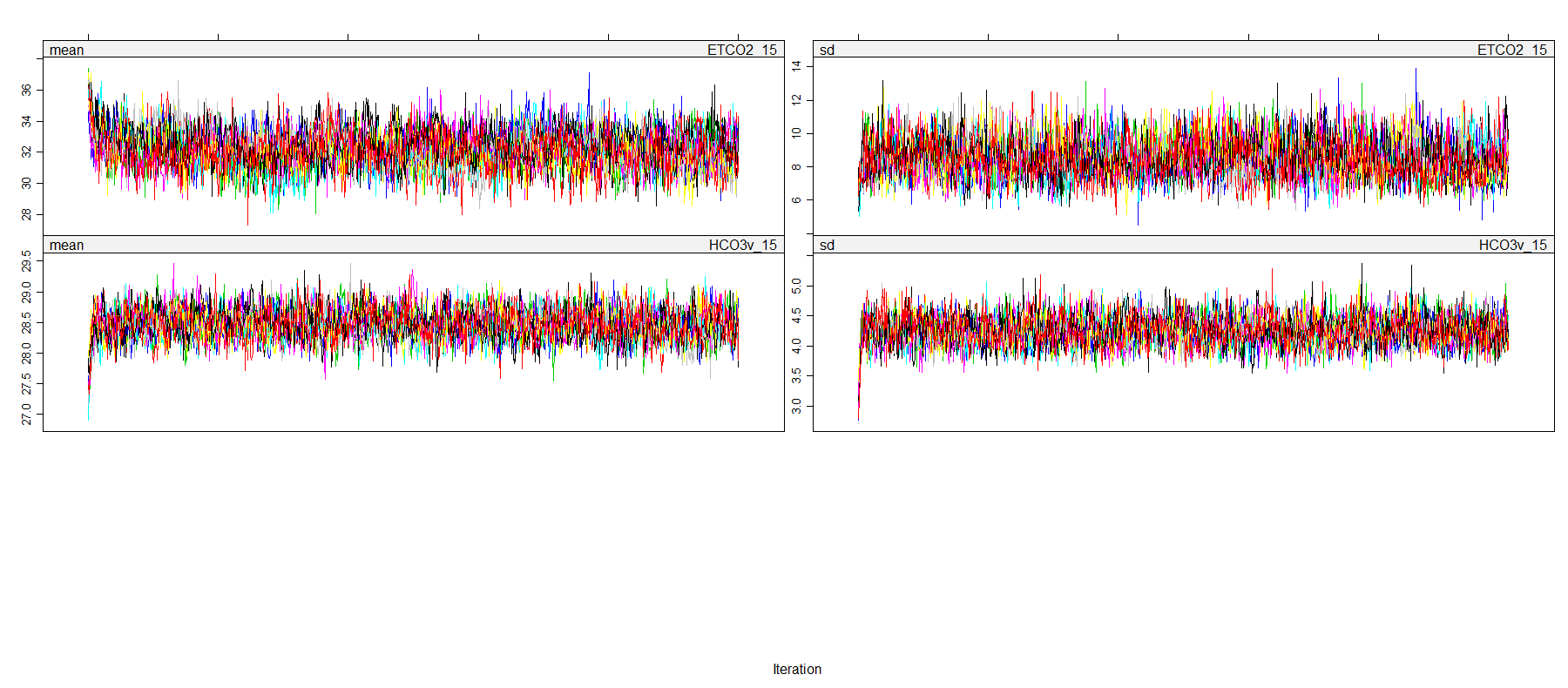
**

Change in mean and standard deviation per imputation iteration cycle for all imputed variables and imputation models (10 models). List of abbreviations: PECO_2_ end- expiratory partial pressure of carbon dioxide; Pes Plat end-inspiratory esophageal pressure; Pes_ee end-expiratory esophageal pressure; PvCO_2_ partial pressure of venous carbon dioxide; PvO_2_ partial pressure of venous oxygen; SvO_2_ venous oxygen saturation; ETCO_2_ end tidal carbon dioxide; HCO3v venous concentration of bicarbonate; sd standard deviation; 5 and 15 refers to the level of PEEP.

**e-Figure 2. Multiple Imputation – Distribution plots**

Distribution curves for imputed variables. Plotted in red are the distributions of the variables post imputation (10 models), as opposed to the original distribution which is plotted in blue. List of abbreviations: PECO_2_ end- expiratory partial pressure of carbon dioxide; Pes Plat end-inspiratory esophageal pressure; Pes_ee end-expiratory esophageal pressure; PvCO_2_ partial pressure of venous carbon dioxide; PvO_2_ partial pressure of venous oxygen; SvO_2_ venous oxygen saturation; ETCO_2_ end tidal carbon dioxide; HCO3v venous concentration of bicarbonate; BE basale base excess baseline; 5 and 15 refers to the level of PEEP.


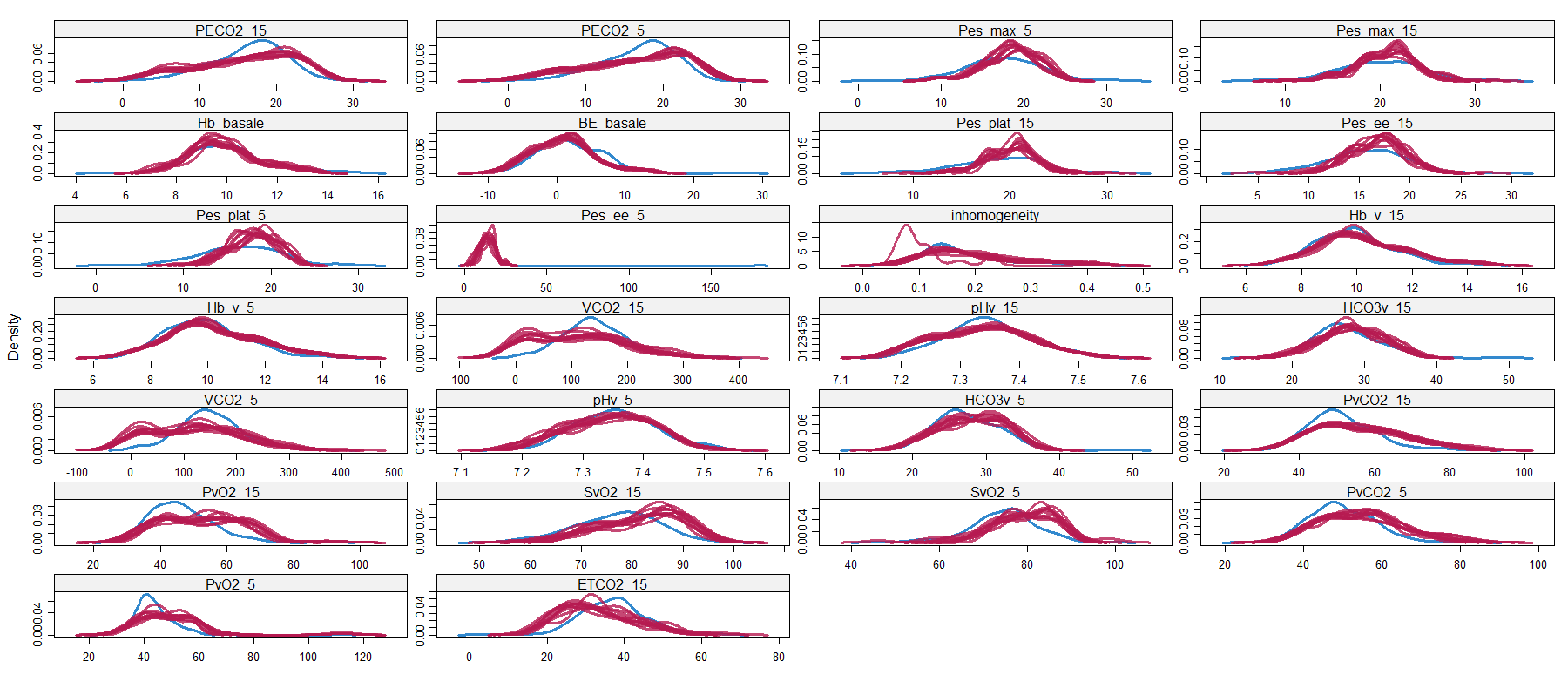

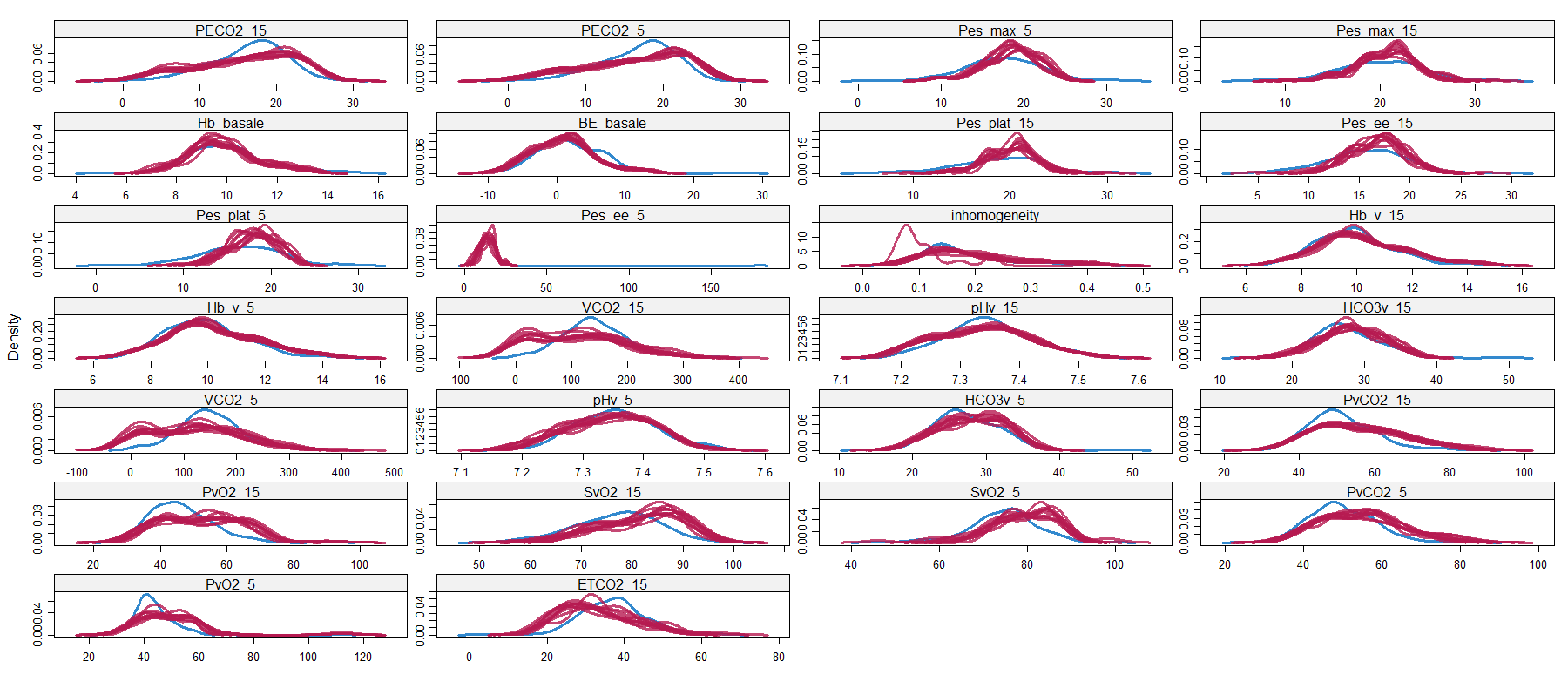

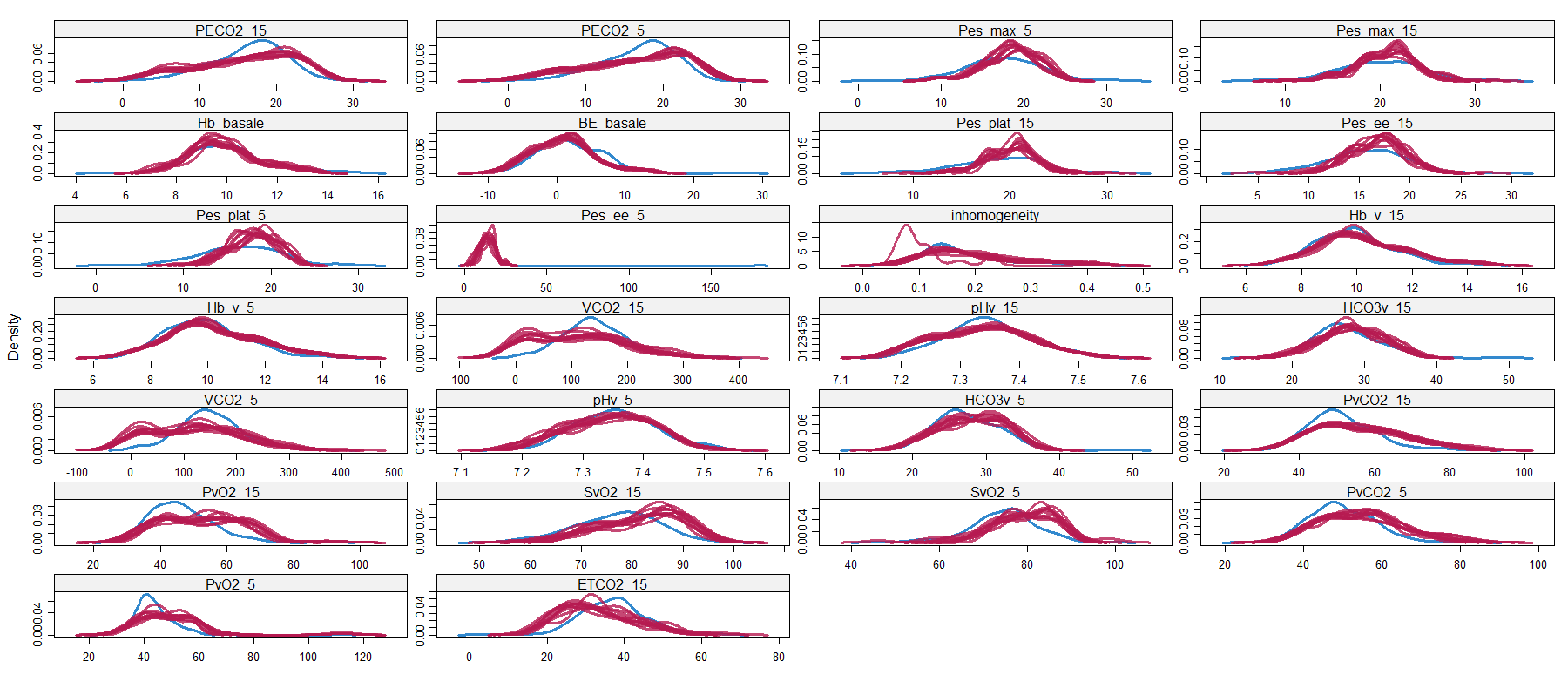

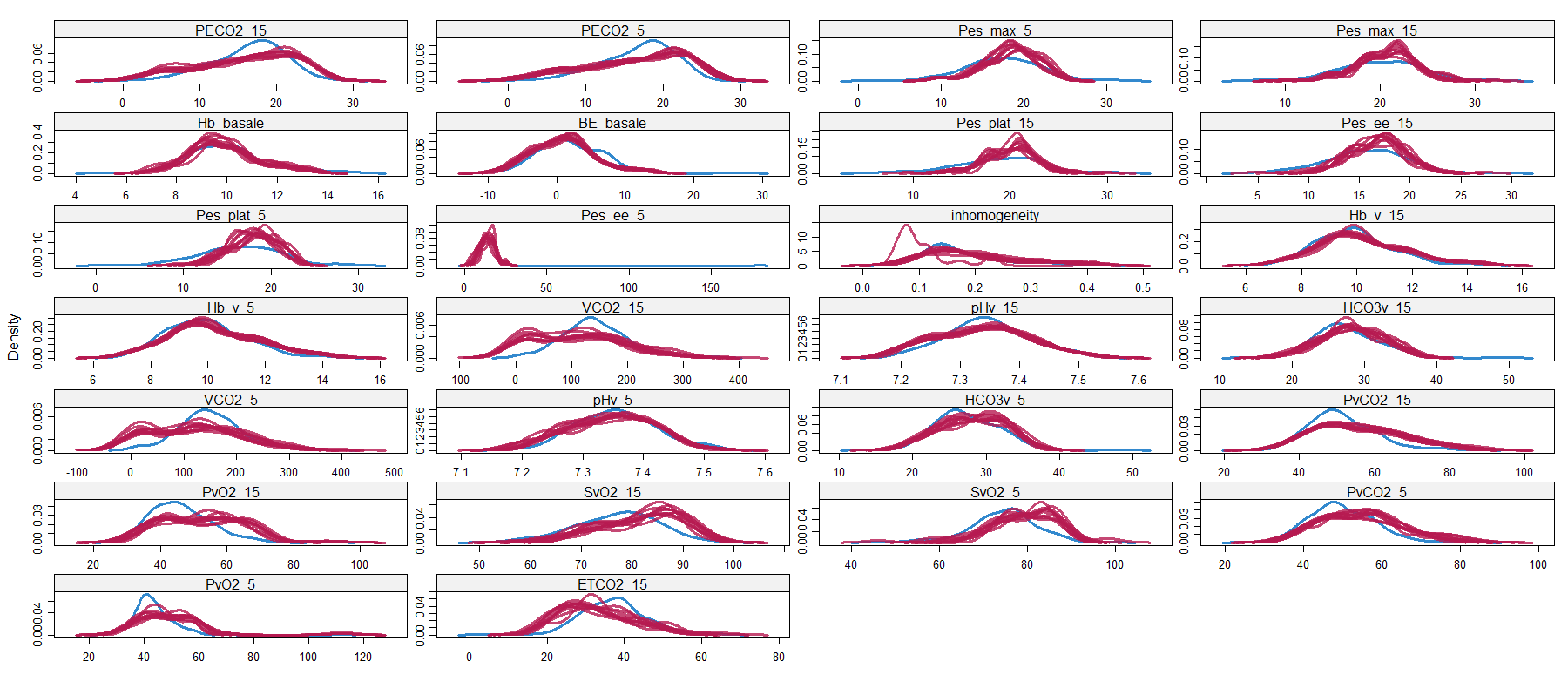


**e-Table 2. Internal validity analysis of LCA class assignment**

| Omitting years 2003 - 2006 | | | | | | | | |
| --- | --- | --- | --- | --- | --- | --- | --- | --- |
| *N of Classes* | Bayesian Information  Criteria° | Entropy* | Number of individual per Class | | | | | p-value^†^ |
|  |  |  | 1 | 2 | 3 | 4 | 5 |  |
| 1 | 7275 |  | 168 |  |  |  |  |  |
| 2 | 7352 | 0.83 | 78 | 90 |  |  |  | 0.001 |
| 3 | 7481 | 0.90 | 61 | 54 | 53 |  |  | 0.560 |
| 4 | 7628 | 0.93 | 46 | 55 | 25 | 42 |  | 0.514 |
| 5 | 7795 | 0.96 | 30 | 44 | 31 | 28 | 35 | 0.853 |

| Omitting years 2007 - 2010 | | | | | | | | |
| --- | --- | --- | --- | --- | --- | --- | --- | --- |
| *N of Classes* | Bayesian Information  Criteria° | Entropy* | Number of individual per Class | | | | | p-value^†^ |
|  |  |  | 1 | 2 | 3 | 4 | 5 |  |
| 1 | 8253 |  | 191 |  |  |  |  |  |
| 2 | 8248 | 0.89 | 65 | 126 |  |  |  | 0.001 |
| 3 | 8369 | 0.95 | 64 | 80 | 47 |  |  | 0.289 |
| 4 | 8504 | 0.95 | 38 | 45 | 44 | 64 |  | 0.535 |
| 5 | 8664 | 0.96 | 35 | 38 | 23 | 37 | 58 | 0.922 |

| Omitting years 2011 - 2014 | | | | | | | | |
| --- | --- | --- | --- | --- | --- | --- | --- | --- |
| *N of Classes* | Bayesian Information  Criteria° | Entropy* | Number of individual per Class | | | | | p-value^†^ |
|  |  |  | 1 | 2 | 3 | 4 | 5 |  |
| 1 | 7582 |  | 175 |  |  |  |  |  |
| 2 | 7566 | 0.87 | 85 | 90 |  |  |  | 0.001 |
| 3 | 7684 | 0.96 | 53 | 21 | 101 |  |  | 0.124 |
| 4 | 7820 | 0.95 | 55 | 50 | 45 | 25 |  | 0.280 |
| 5 | 7990 | 0.97 | 25 | 38 | 37 | 35 | 40 | 0.514 |

| Omitting years 2015 - 2019 | | | | | | | | |
| --- | --- | --- | --- | --- | --- | --- | --- | --- |
| *N of Classes* | Bayesian Information  Criteria° | Entropy* | Number of individual per Class | | | | | p-value^†^ |
|  |  |  | 1 | 2 | 3 | 4 | 5 |  |
| 1 | 7784 |  | 180 |  |  |  |  |  |
| 2 | 7780 | 0.92 | 67 | 113 |  |  |  | 0.001 |
| 3 | 7907 | 0.93 | 77 | 60 | 43 |  |  | 0.212 |
| 4 | 8053 | 0.96 | 22 | 70 | 45 | 43 |  | 0.537 |
| 5 | 8228 | 0.97 | 52 | 34 | 29 | 21 | 44 | 0.687 |

**°**Bayesian Information Criterion (BIC) is a likelihood function derived criterion for model selection among a set of models; lower BICs indicate better model fit. *****Entropy is a measure to assess the degree of association between an individual and a class based on the posterior class membership probabilities; values above 0.8 define good class distinction. **^†^**The p-value is calculated by means of the bootstrap likelihood ratio test, it addresses if a model with k classes provides increased fit compared to a model with k-1 classes.

**e-Figure 3. Internal validity analysis of LCA class assignment – Kaplan Meier Curves**

**
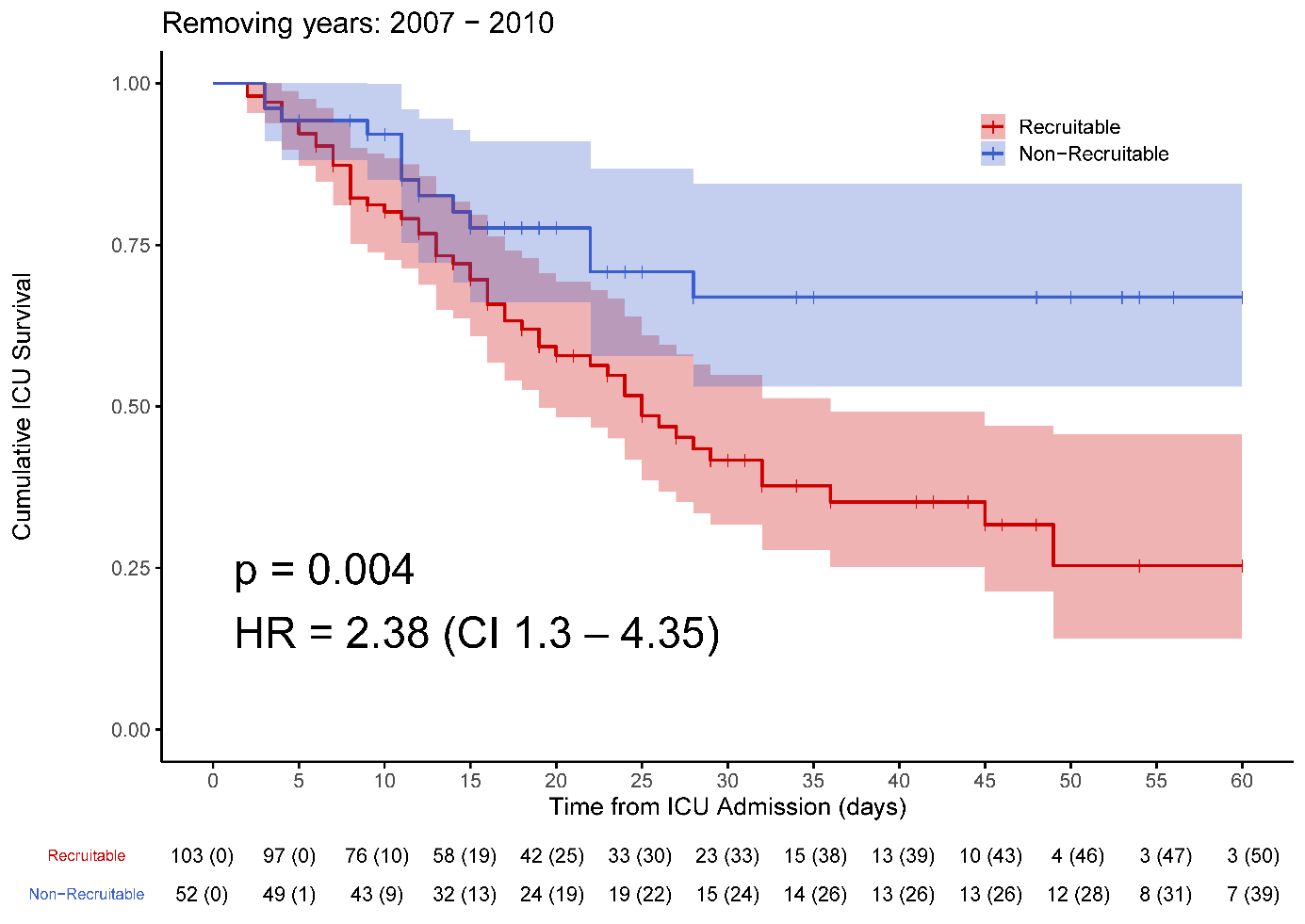

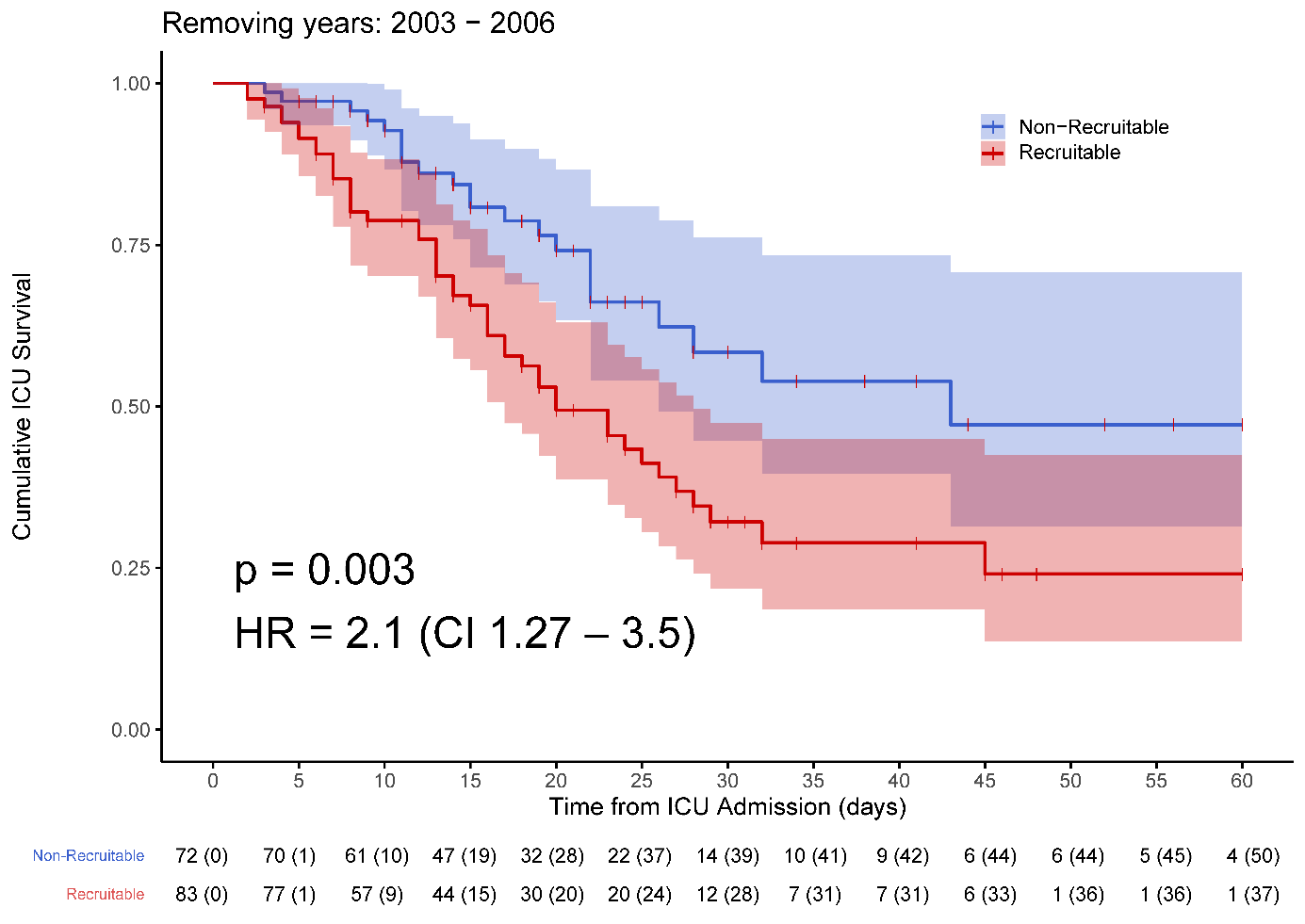
**

**
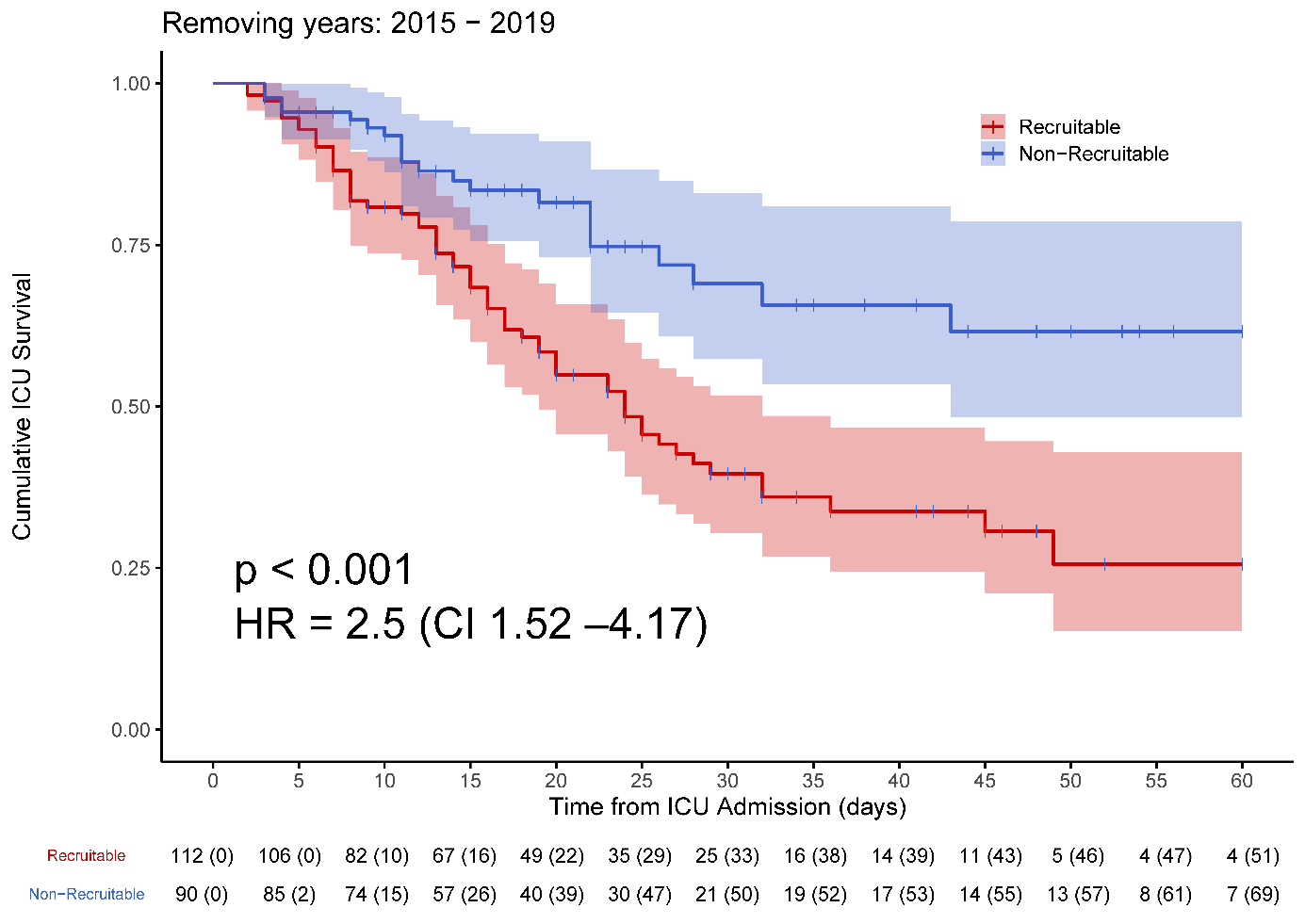

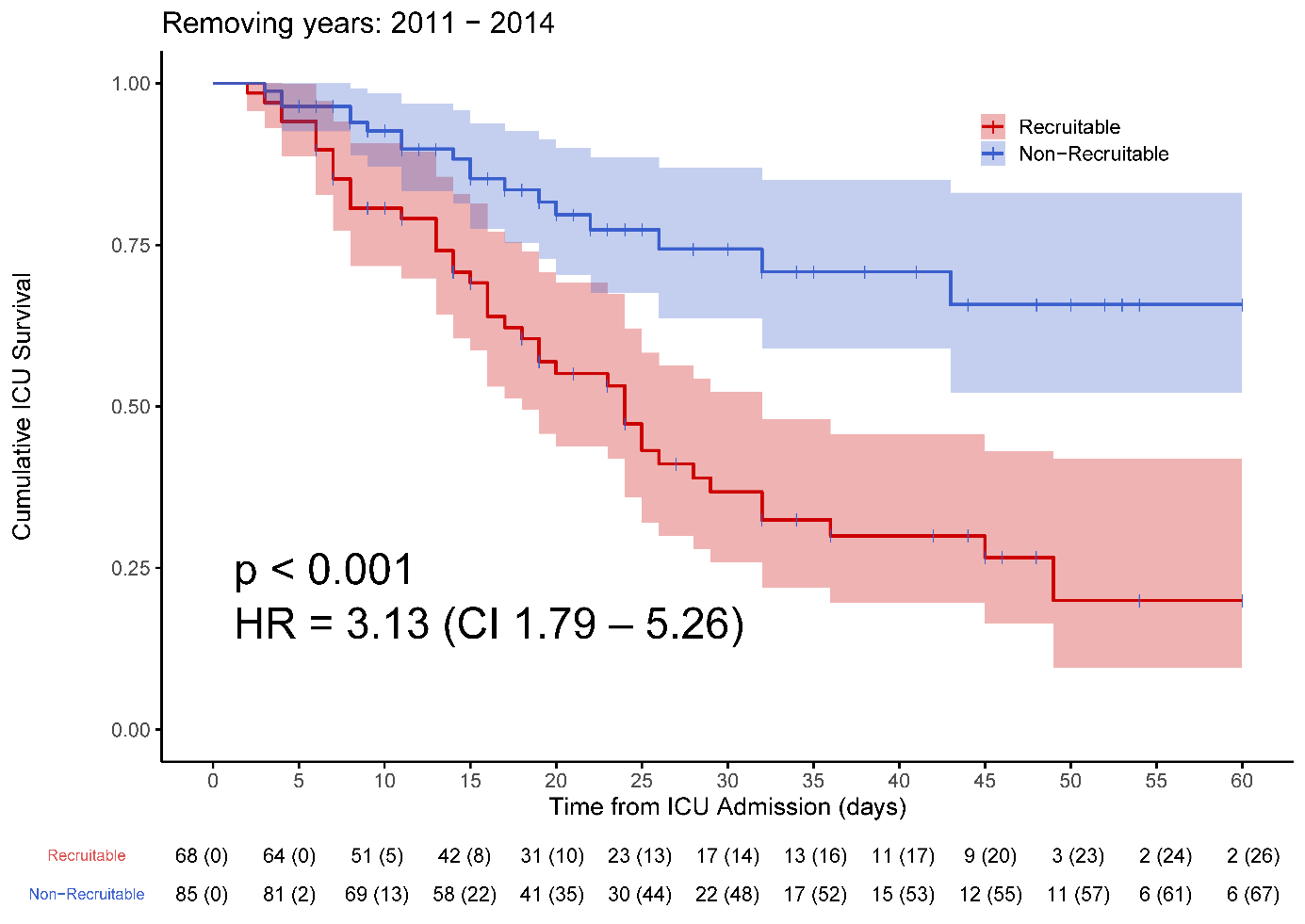
**

Kaplan-Meier curve for 60-day intensive care unit survival stratified by Latent Class Analysis (LCA) derived phenotype. Phenotypes non-recruitable and recruitable are plotted in blue and red colors respectively, shaded areas represent the 95% Confidence Interval (CI). The computed hazard ratio (HR) assesses the recruitable using the non-recruitable phenotype as reference, 95% CI is given in parentheses. HRs are modelled by means of a Fine and Gray competing risk analysis. Censoring reflects patients having left the ICU alive. The underlying table presents the patients at risk per time point with the number of censored patients given in parentheses.

**e-Table 3. Imputation model dependent latent class transitions and outcome data**

|  |  | **Model 1*** | | **Model 2** | | **Model 3** | | **Model 4** | | **Model 5** | | **Model 6** | | **Model 7** | | **Model 8** | | **Model 9** | | **Model 10** | |
| --- | --- | --- | --- | --- | --- | --- | --- | --- | --- | --- | --- | --- | --- | --- | --- | --- | --- | --- | --- | --- | --- |
|  |  | **Class 1** | **Class 2** | **Class 1** | **Class  2** | **Class 1** | **Class 2** | **Class 1** | **Class**  **2** | **Class 1** | **Class 2** | **Class 1** | **Class 2** | **Class 1** | **Class  2** | **Class 1** | **Class**  **2** | **Class 1** | **Class  2** | **Class**  **1** | **Class**  **2** |
| **n** |  | 106 | 132 | 124 | 114 | 122 | 116 | 111 | 127 | 117 | 121 | 119 | 119 | 107 | 131 | 123 | 115 | 111 | 127 | 109 | 129 |
| **SAPS II** |  | 42  ±  15 | 45  ±  15 | 44  ±  15 | 43  ± 15 | 43  ±  15 | 44  ±  15 | 43 ±  15 | 44 ±  15 | 44  ±  16 | 43  ±  15 | 43  ± 15 | 44  ±  16 | 43  ±  15 | 44  ±  15 | 43  ±  16 | 44  ±  15 | 43  ±  15 | 44 ± 15 | 43  ±  15 | 44  ±  15 |
|  |  |  |  |  |  |  |  |  |  |  |  |  |  |  |  |  |  |  |  |  |  |
| **ARDS Origin** | Extra Pulmonary | 55 (52%) | 41  (31%) | 60  (48%) | 36  (32%) | 61  (50%) | 35  (30%) | 58  (52%) | 38  (30%) | 60  (51%) | 36  (30%) | 61  (51%) | 35  (29%) | 55  (51%) | 41  (31%) | 62  (50%) | 34  (30%) | 55  (50%) | 41  (32%) | 57  (52%) | 39  (30%) |
|  | Pulmonary | 51  (48%) | 91  (69%) | 64  (52%) | 78  (68%) | 61  (50%) | 81  (70%) | 53  (48%) | 89  (70%) | 57  (49%) | 85  (70%) | 58  (49%) | 84  (71%) | 52  (49%) | 90  (69%) | 61  (50%) | 81  (70%) | 56  (50%) | 86  (68%) | 52  (48%) | 90  (70%) |
| **paO_2_/FiO_2_ Ratio** |  | 199  ±  62 | 154  ±  60 | 197  ±  63 | 148  ±  56 | 197  ±  64 | 147  ±  55 | 195  ± 63 | 154  ±  60 | 196  ±  65 | 151  ±  57 | 195  ± 63 | 151  ±  59 | 193  ± 62 | 157  ±  63 | 197  ±  65 | 148  ± 54 | 195  ±  63 | 154  ±  60 | 198  ±  63 | 153  ±  59 |
| **ARDS Severity** | Mild | 49 (46%) | 25  (19%) | 57  (46%) | 17  (15%) | 56  (46%) | 17  (15%) | 48  (43%) | 25  (20%) | 52  (44%) | 21  (17%) | 54  (45%) | 20  (17%) | 45  (42%) | 28  (21%) | 56  (46%) | 17  (15%) | 48  (43%) | 25  (20%) | 49  (45%) | 25  (19%) |
|  | Moderate | 54  (51%) | 81  (61%) | 61  (49%) | 74  (65%) | 61  (50%) | 74  (64%) | 59  (53%) | 77  (61%) | 60  (51%) | 76  (63%) | 60  (50%) | 74  (62%) | 58  (54%) | 78  (60%) | 62  (50%) | 74  (64%) | 60  (54%) | 76  (60%) | 56  (51%) | 76  (61%) |
|  | Severe | 3  (3%) | 26  (20%) | 6  (5%) | 23  (20%) | 5  (4%) | 25  (22%) | 4  (4%) | 25  (20%) | 5  (4%) | 24  (20%) | 5  (4%) | 25  (21%) | 4  (4%) | 25  (19%) | 5  (4%) | 24  (21%) | 3  (3%) | 26  (20%) | 4  (4%) | 25  (19%) |
|  |  |  |  |  |  |  |  |  |  |  |  |  |  |  |  |  |  |  |  |  |  |
| **Recruitability** |  | 9  ±  13 | 21  ±  14 | 7  ±  17 | 20  ±  19 | 9  ±  15 | 22  ±  14 | 8  ±  13 | 18 ±  23 | 7  ±  17 | 20  ±  21 | 8  ±  14 | 23  ±  14 | 8  ±  14 | 20  ±  18 | 9  ±  16 | 21 ± 17 | 8  ±  16 | 20 ± 21 | 7  ±  16 | 20  ± 19 |
|  |  |  |  |  |  |  |  |  |  |  |  |  |  |  |  |  |  |  |  |  |  |
| **ICU Mortality** |  | 27 (23%) | 69  (52%) | 34 (26%) | 62  (54%) | 33 (26%) | 63 (54%) | 29 (25%) | 67 (52%) | 35  (27%) | 63 (52%) | 34  (28%) | 62 (51%) | 27  (25%) | 69 (48%) | 35  (27%) | 61 (53%) | 28  (24%) | 68 (53%) | 29 (25%) | 67 (52%) |
| **LOS ICU** |  | 26  ±  25 | 23  ±  23 | 26  ±  28 | 22 ±  17 | 24  ±  23 | 24  ±  24 | 26  ±  24 | 22  ±  23 | 27  ±  29 | 21  ±  17 | 25  ±  24 | 23  ±  24 | 26  ±  24 | 22  ±  23 | 26  ±  28 | 22  ± 17 | 25  ±  24 | 23  ±  23 | 25  ±  24 | 23  ±  23 |
|  |  |  |  |  |  |  |  |  |  |  |  |  |  |  |  |  |  |  |  |  |  |
| **Class Transitions compared to Model 1** | | 0  (0%) | 0  (0%) | 25  (20%) | 7  (6%) | 24  (20%) | 8  (7%) | 13  (12%) | 8  (6%) | 20  (17%) | 9  (7%) | 24  (20%) | 11 (9%) | 13  (12%) | 12 (9%) | 24  (19%) | 7  (6%) | 12  (11%) | 7  (6%) | 11 (10%) | 8  (6%) |

SAPS II: simplified acute physiologic score II; ARDS: Acute Respitratory Distress Syndrome; ICU: Intensive Care Unit; LOS: Length of Stay. Quantitative data are expressed as mean (± Standard Deviation). Categorical data are presented as N (number of subjects) and percentages (%).

**e-Figure 4. Imputation model dependent Kaplan Meier Curves**

**
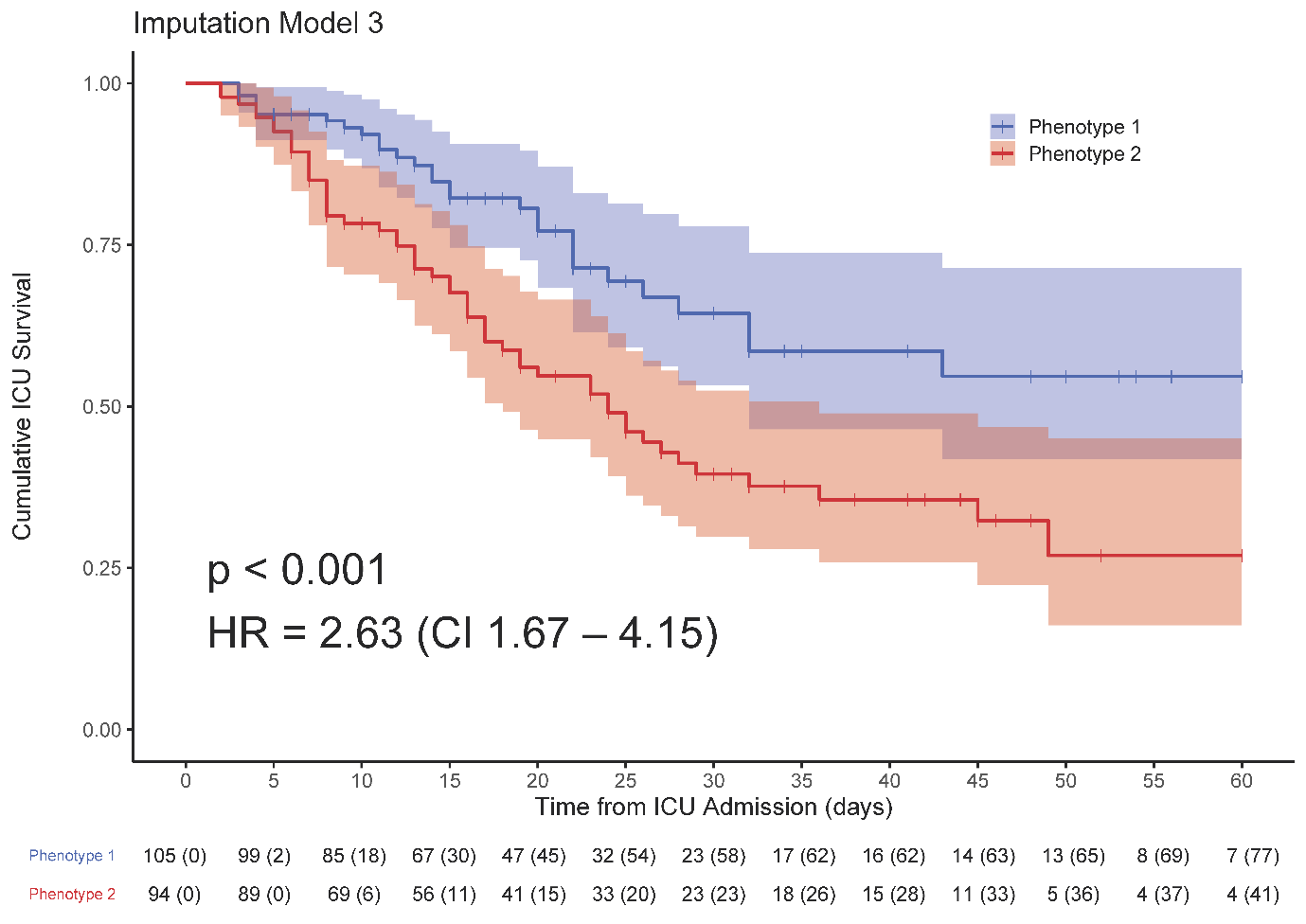

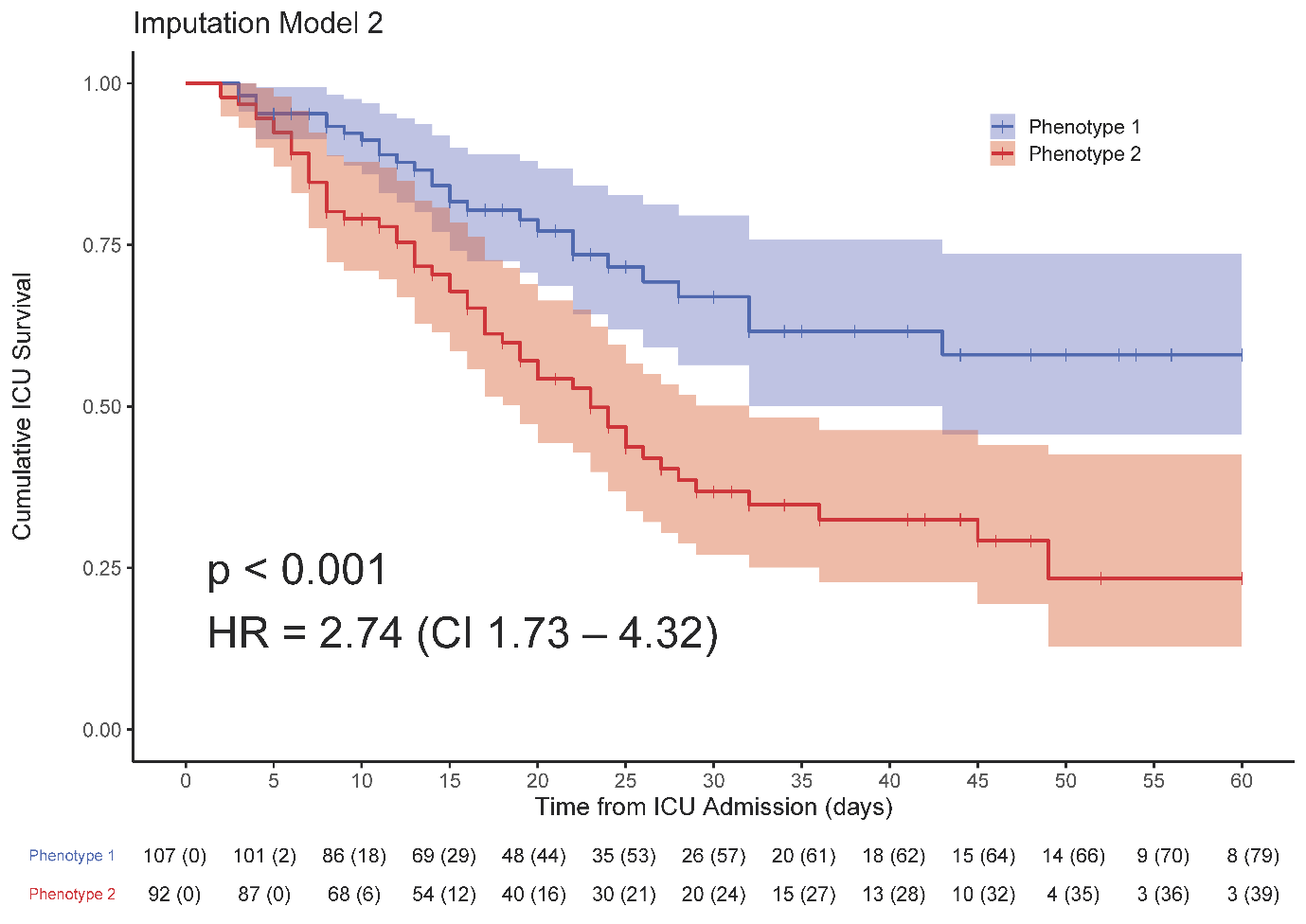
**

**
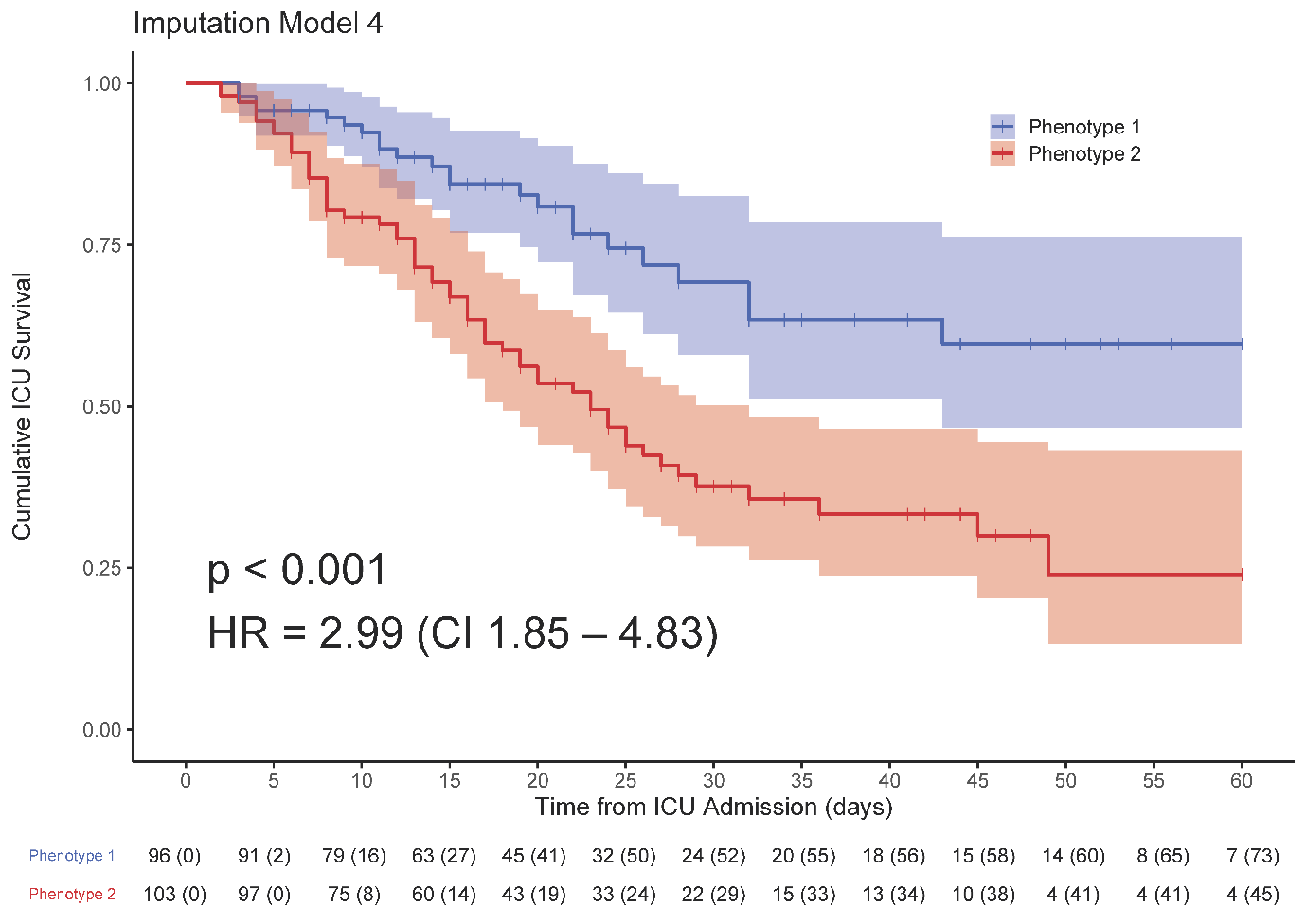

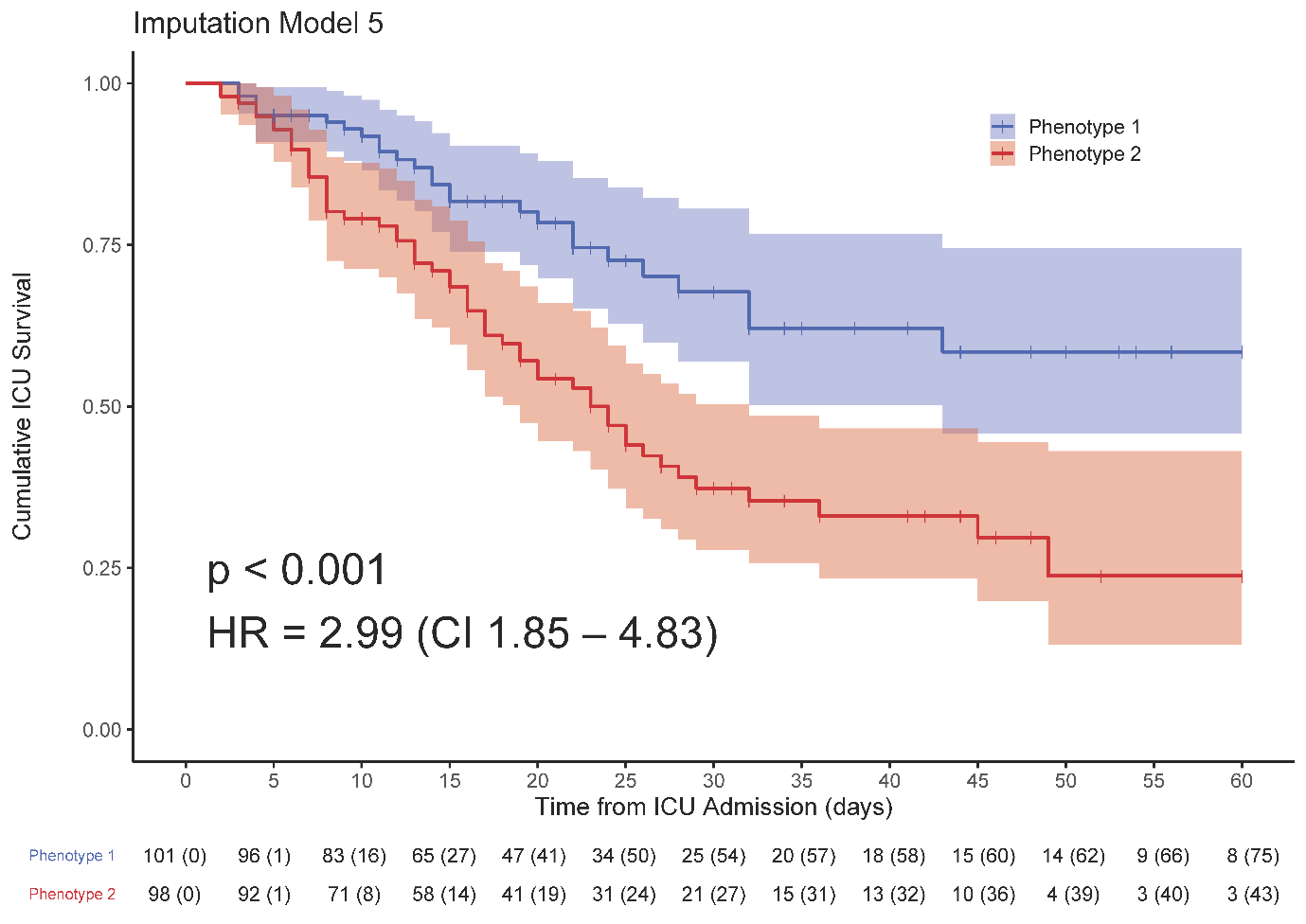
**

**
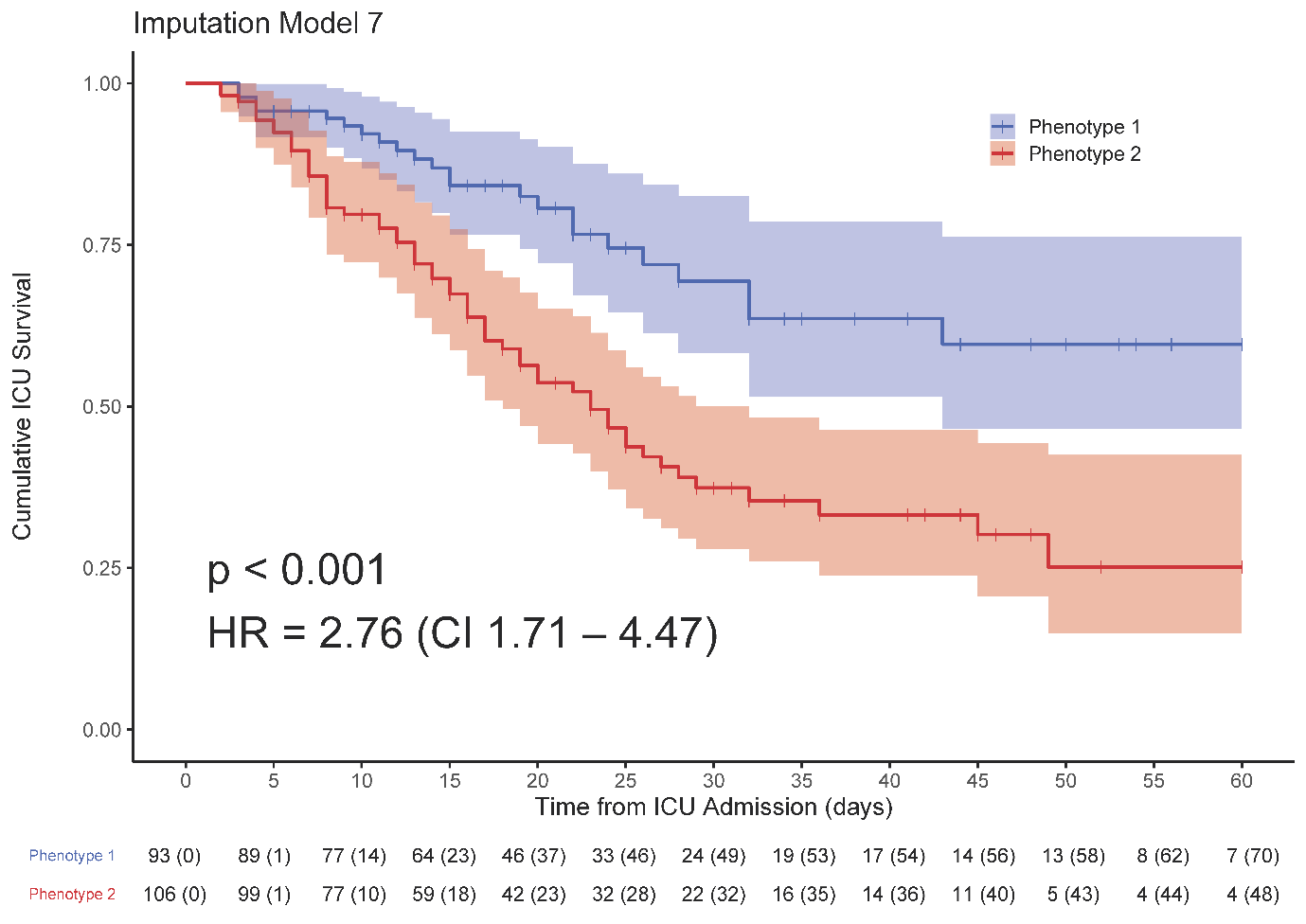

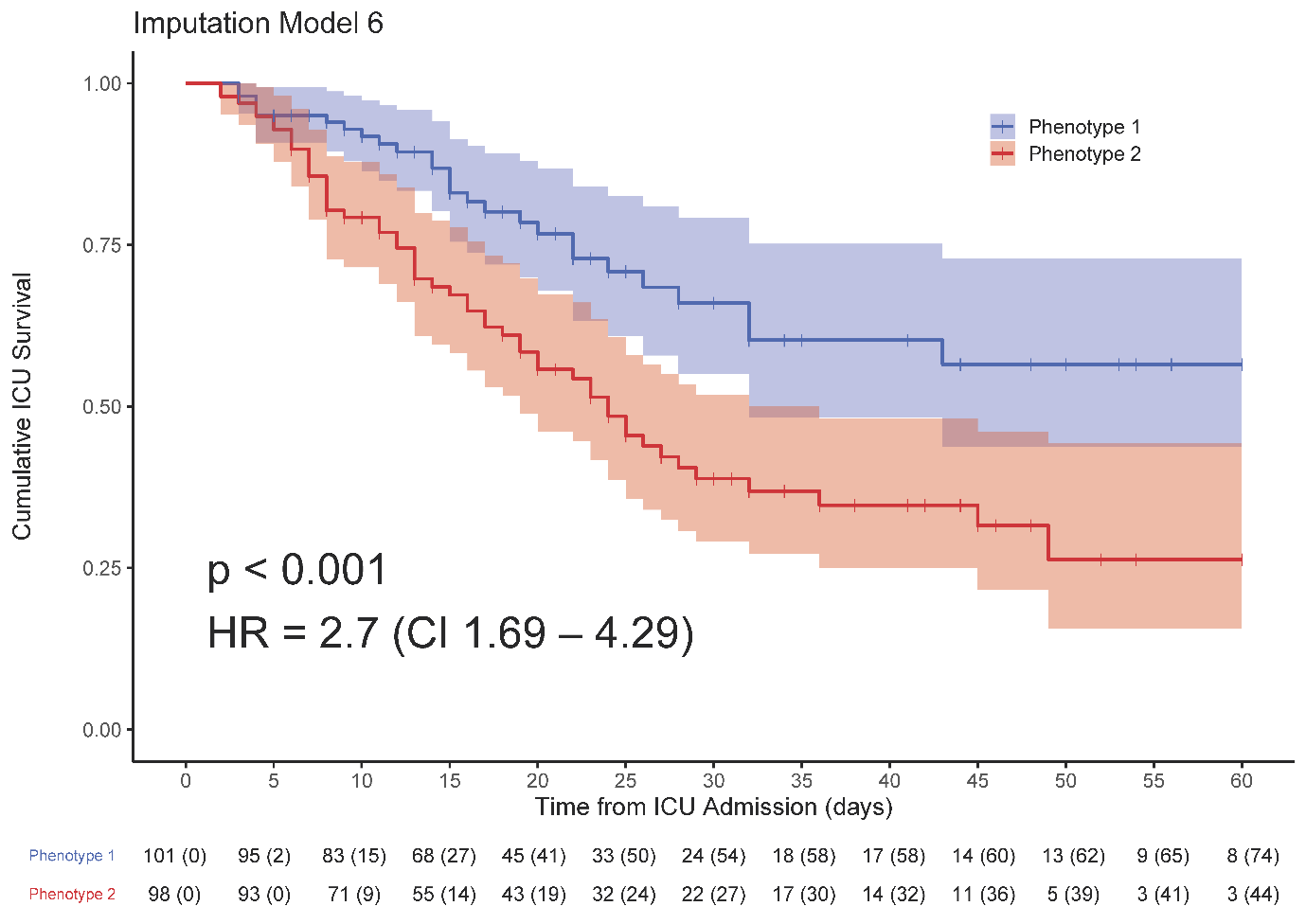
**

**
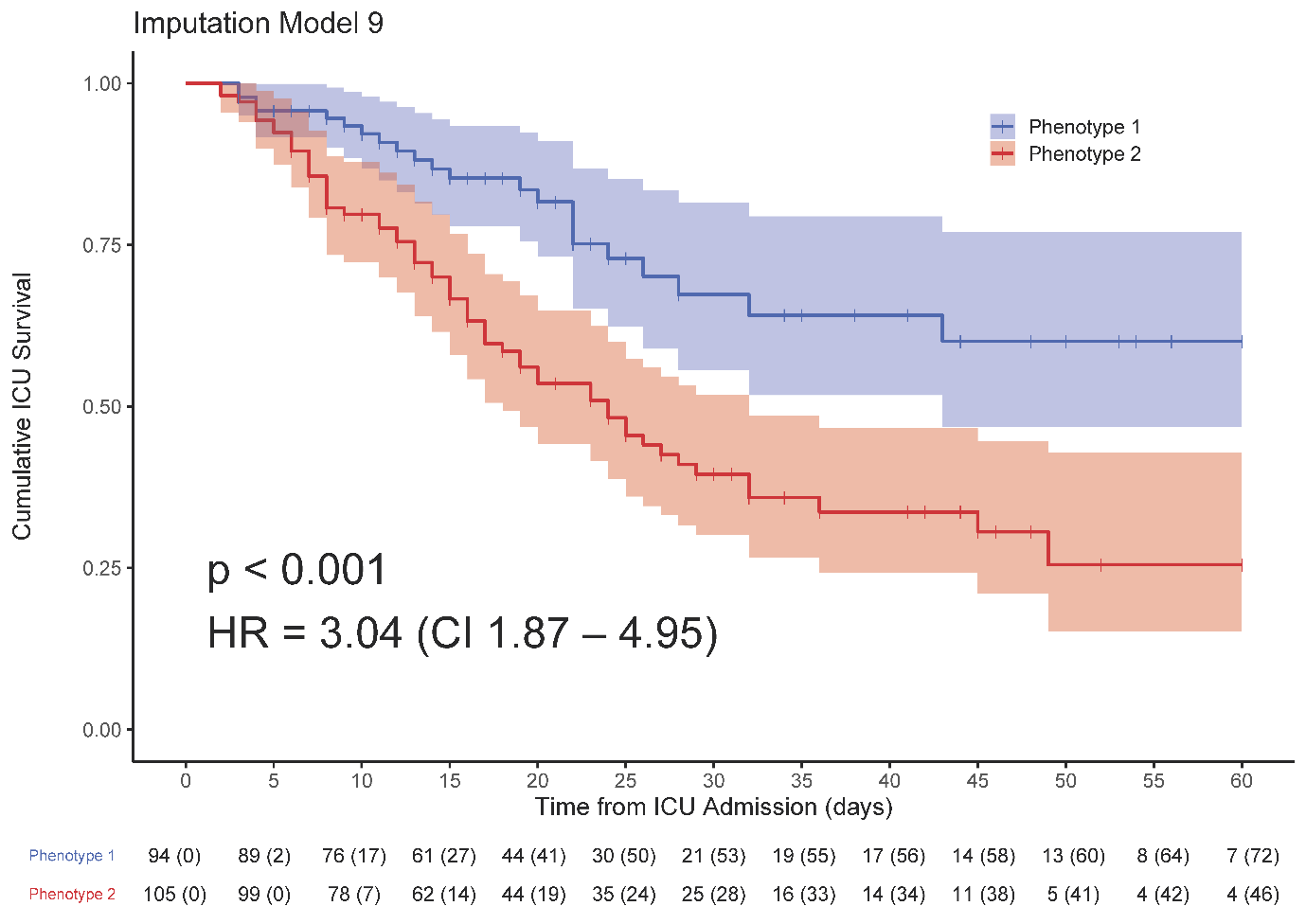

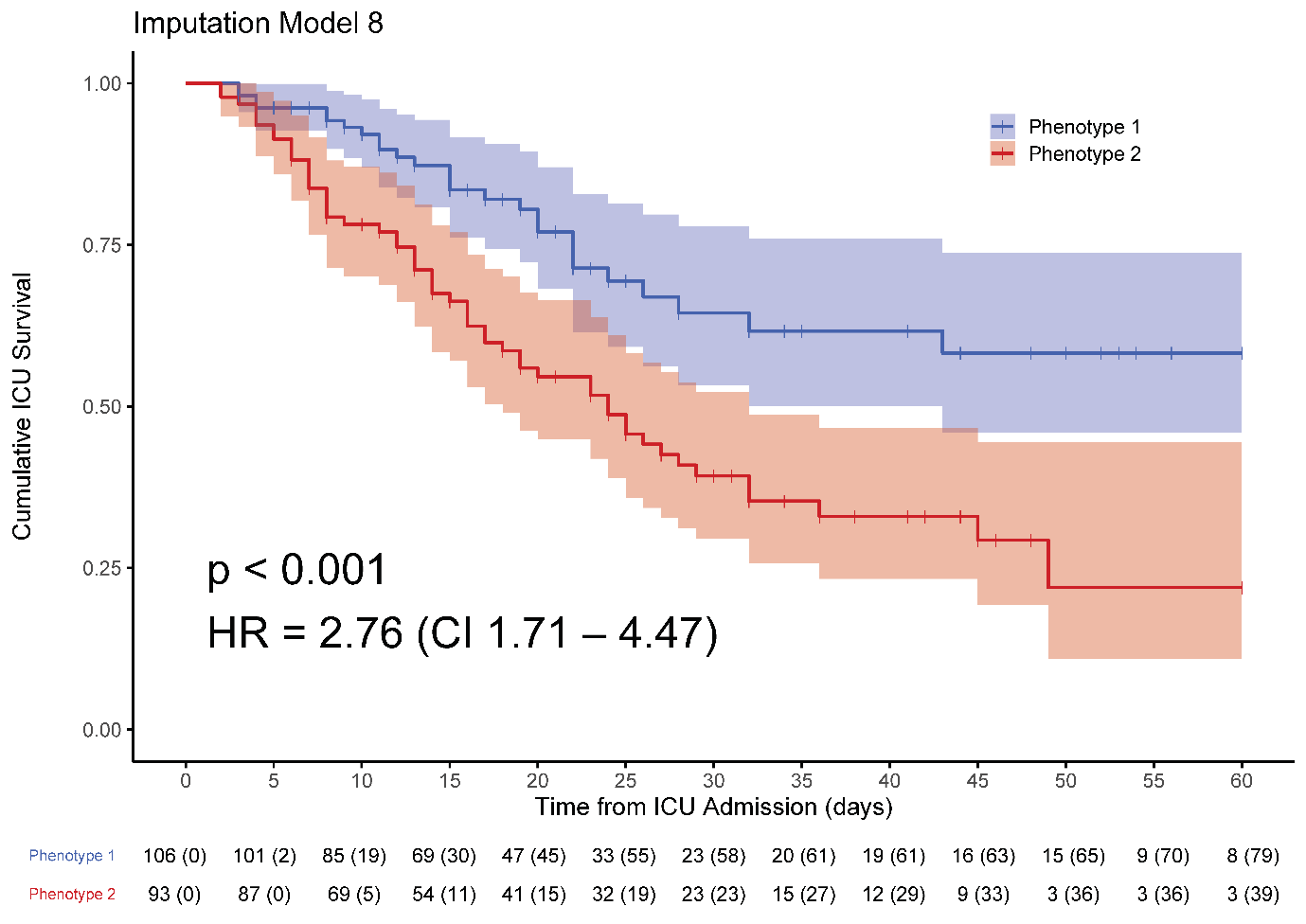
**

**
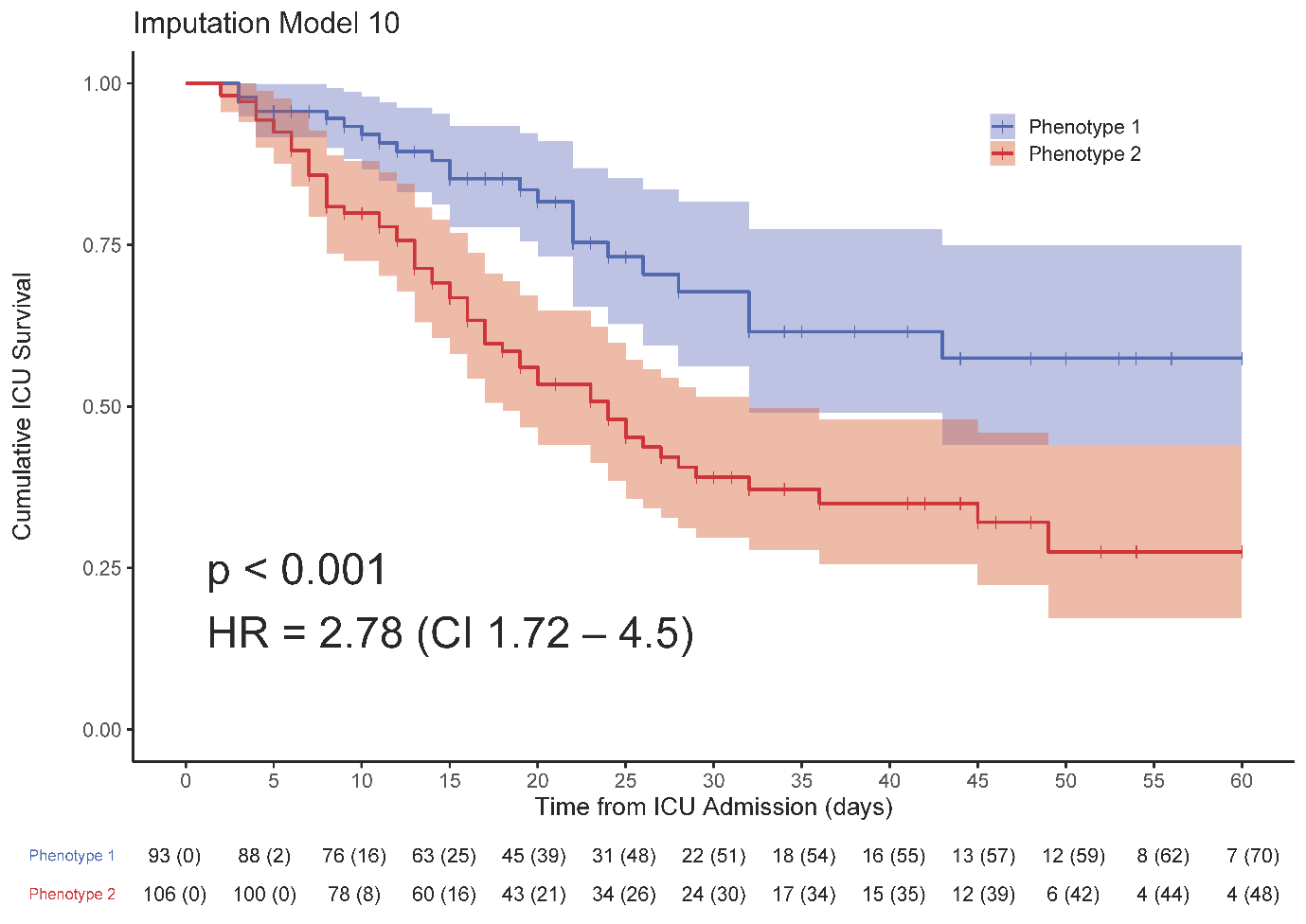
**

Kaplan-Meier curve for 60-day intensive care unit survival stratified by Latent Class Analysis (LCA) derived phenotype. Phenotypes 1 and 2 are plotted in blue and red colors respectively, shaded areas represent the 95% Confidence Interval (CI). The computed hazard ratio (HR) assesses phenotype 2 using phenotype 1 as reference, 95% CI is given in parentheses. HRs are modelled by means of a Fine and Gray competing risk analysis. Censoring reflects patients having left the ICU alive. The underlying table presents the patients at risk per time point with the number of censored patients given in parentheses.

**e-Appendix 2. Complete case sensitivity analysis**

| *N of Classes* | Bayesian Information  Criteria° | Entropy* | Number of individual per Class | | | | | p-value^†^ |
| --- | --- | --- | --- | --- | --- | --- | --- | --- |
|  |  |  | 1 | 2 | 3 | 4 | 5 |  |
| 1 | 7327 |  | 169 |  |  |  |  |  |
| 2 | 7353 | 0.91 | 104 | 65 |  |  |  | 0.009 |
| 3 | 7486 | 0.92 | 57 | 46 | 66 |  |  | 0.297 |
| 4 | 7632 | 0.95 | 57 | 42 | 46 | 24 |  | 0.810 |
| 5 | 7789 | 0.97 | 40 | 17 | 46 | 48 | 18 | 0.211 |

°Bayesian Information Criterion (BIC) is a likelihood function derived criterion for model selection among a set of models; lower BICs indicate better model fit. *Entropy is a measure to assess the degree of association between an individual and a class based on the posterior class membership probabilities; values above 0.8 define good class distinction. †The p-value is calculated by means of the bootstrap likelihood ratio test, it addresses if a model with k classes provides increased fit compared to a model with k-1 classes.

|  |  | **Phenotype 1** | **Phenotype 2** |
| --- | --- | --- | --- |
|  |  | **Non-Recruitable** | **Recruitable** |
| **n** |  | 104 | 65 |
| **SAPS II** |  | 43  ±  16 | 45  ±  15 |
|  |  |  |  |
| **ARDS Origin** | Extra Pulmonary | 58  (56%) | 20  (31%) |
|  | Pulmonary | 46  (54%) | 45  (69%) |
| **paO_2_/FiO_2_ Ratio** |  | 204  ±  65 | 151  ±  56 |
| **ARDS Severity** | Mild | 54 (52%) | 11  (17%) |
|  | Moderate | 45  (43%) | 44  (68%) |
|  | Severe | 5  (5%) | 10  (15%) |
|  |  |  |  |
| **Recruitability** |  | 11  ±  9 | 21  ±  13 |
|  |  |  |  |
| **ICU Mortality** |  | 26 (25%) | 30  (46%) |
| **LOS ICU** |  | 28  ±  29 | 21  ±  19 |
|  |  |  |  |
| **Class Transitions compared to Model 1** |  | 12  (12%) | 0  (0%) |

SAPS II: simplified acute physiologic score II; ARDS: Acute Respitratory Distress Syndrome; ICU: Intensive Care Unit; LOS: Length of Stay. Quantitative data are expressed as mean (± Standard Deviation). Categorical data are presented as N (number of subjects) and percentages (%).

**
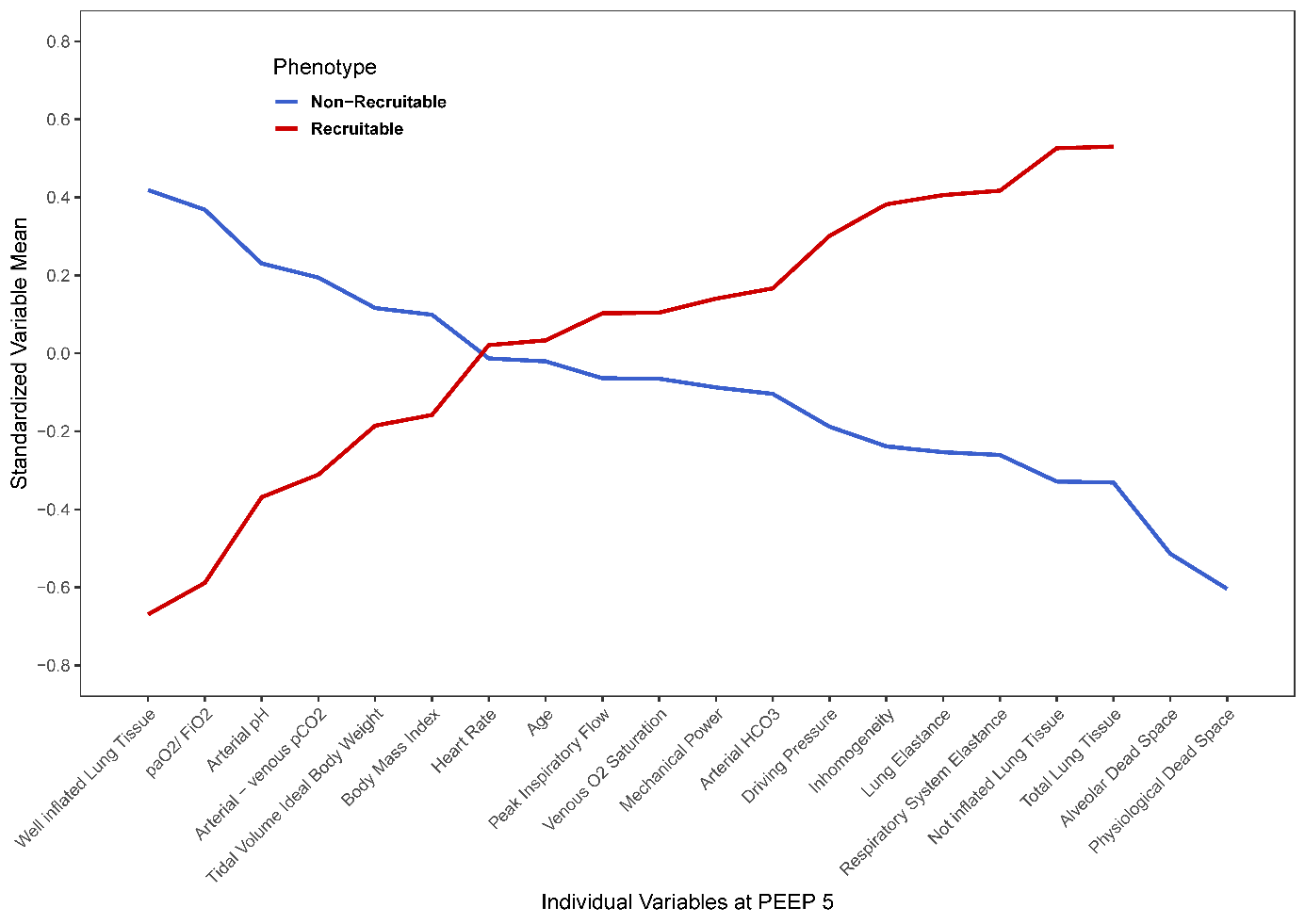
**

Continuous variables are standardized to a mean of 0 and a standard deviation of 1. The order of variables is defined such that the standardized variable mean, plotted on the y-axis, is highest on the right side of the plot for phenotype 1. PEEP: positive end-expiratory pressure; paO_2_: partial pressure of arterial oxygen; FiO_2_: fraction of inspired oxygen; pCO_2_: partial pressure of carbon dioxide.

**
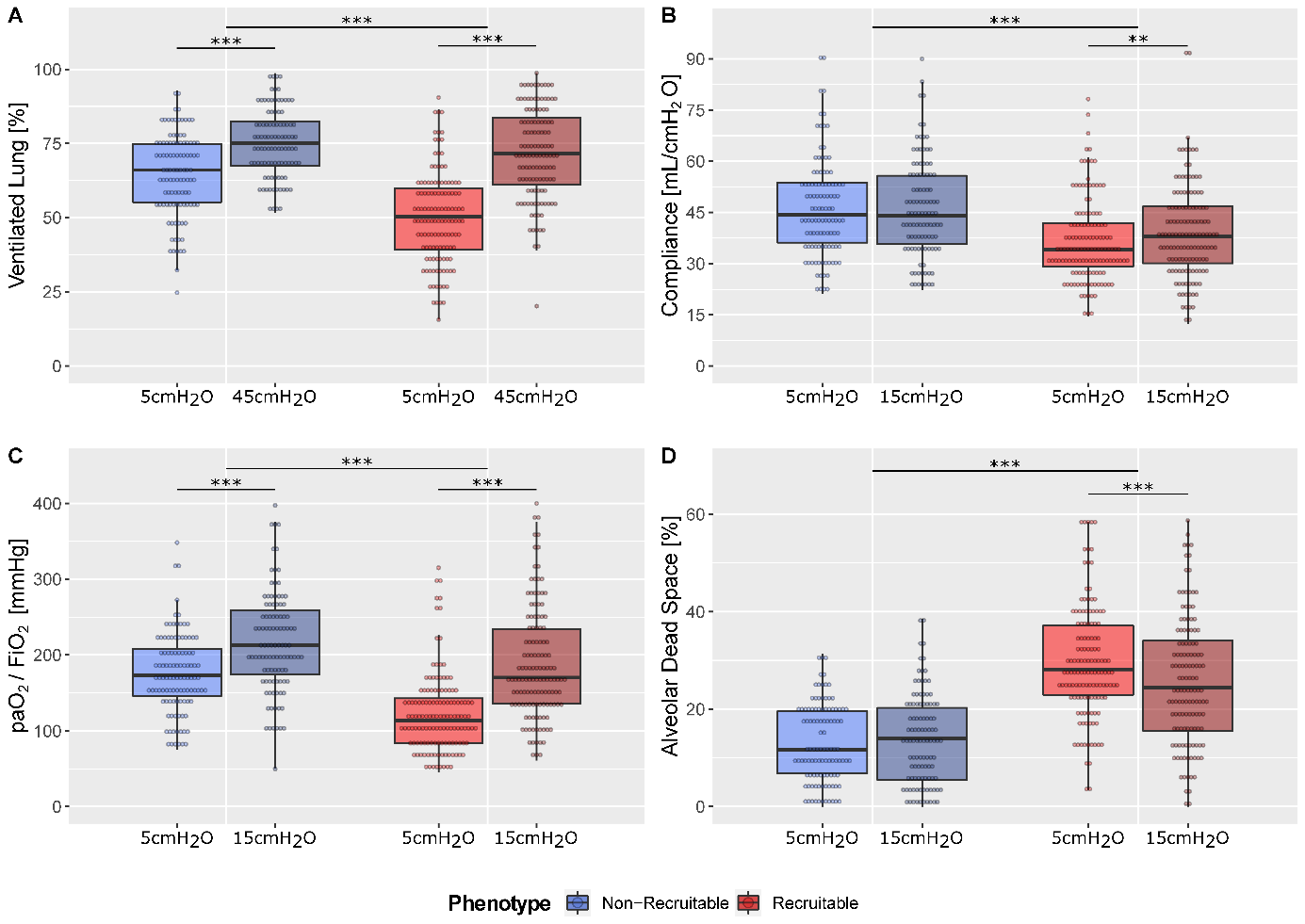
**

Box-plots present (A) the amount of ventilated lung, defined as the cumulative CT-graphicly inferred poorly, well and over-inflated lung, (B) the respiratory system compliance, (C) the paO_2_/ FiO_2_ ratio and (D) the alveolar dead space at two stages of a recruitment maneuver for the *non-recruitable* and *recruitable* LCA phenotypes. (A) was measured during a inspiratory hold maneuver at an end-inspiratory airway pressures of 5 and 45 cmH_2_O; (B), (C) and (D) were measured under positive end-expiratory pressures of 5 and 15 cmH_2_O. p-values *<0.05, **<0.01, ***<0.001 for differences in recruitment maneuver between phenotypes and pressures.

**
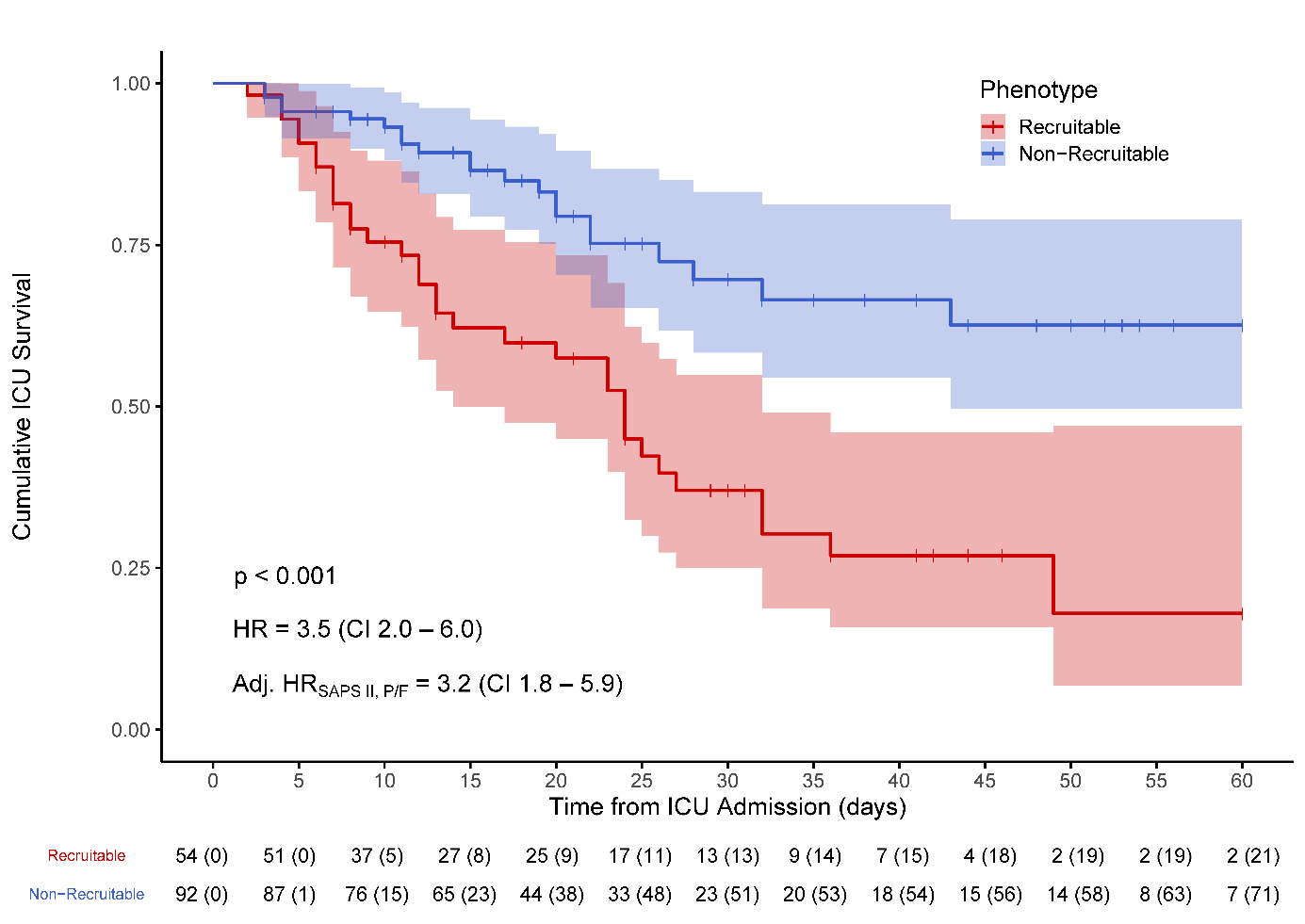
**

Kaplan-Meier curve for 60-day intensive care unit survival stratified by Latent Class Analysis (LCA) derived phenotype. *Non-recruitable* and *recruitable* phenotypes are plotted in blue and red colors respectively, shaded areas represent the 95% Confidence Interval (CI). The computed hazard ratio (HR) assesses the *recruitable* using the non-*recruitable* phenotype as reference, 95% CI is given in parentheses. HRs are modelled by means of a Fine and Gray competing risk analysis. Crude and adjusted HR for SAPS II and the paO_2_/ FiO_2_ ratio at a PEEP level of 5 cmH_2_O are presented. Censoring reflects patients having left the ICU alive. The underlying table presents the patients at risk per time point with the number of censored patients given in parentheses.

**e-Table 4. Respiratory mechanics - gas exchange and computed tomography data at 5 cmH_2_O of PEEP**

|  | Total Population  N = 238 | Phenotype 1 | Phenotype 2 |
| --- | --- | --- | --- |
|  |  | **“Non-Recruitable”**  N = 106 | **“Recruitable”**  N = 132 |
| Tidal Volume (mL) | 500 [422, 560] | 520 [454, 597] | 478 [418, 535] |
| Respiratory Rate (min^-1^) | 16 [14, 20] | 15 [14, 18] | 18 [15, 20] |
| Minute ventilation (L min^-1^) | 8.4 [7.2, 9.9] | 8.4 [7.5, 9.1] | 8.4 [7.1, 10.2] |
| End-inspiratory airway pressure (cmH_2_O) | 18 [16, 21] | 17 [15, 20] | 19 [16, 22] |
| Driving Pressure (cmH_2_O) | 13 [10, 16] | 12 [9, 14] | 14 [11, 16] |
| Respiratory system elastance (cmH_2_O mL^-1^) | 26 [20, 32] | 23 [19, 28] | 29 [24, 34] |
| Lung elastance (cmH_2_O mL^-1^) | 19 [13, 25] | 14 [10, 21] | 22 [16, 28] |
| Chest wall elastance (cmH_2_O mL^-1^) | 6 [4, 9] | 6 [4, 10] | 6 [4, 9] |
| Elastance derived Transpulmonary pressure (cmH_2_O) | 13 [10, 16] | 11 [8, 15] | 14 [12, 18] |
| Arterial pO_2_ (mmHg) | 71 [62, 82] | 75 [65, 88] | 68 [60, 80] |
| PaO_2_/FiO_2_  (mmHg) | 142 [104, 179] | 173 [146, 208] | 114 [84, 143] |
| Arterial pCO_2_ (mmHg) | 44 [39, 52] | 41 [37, 45] | 49 [42, 55] |
| Physiological dead space (%) | 62 [54, 72] | 52 [46, 58] | 70 [64, 80] |
| Total lung tissue (g) | 1429 [1186, 1721] | 1302 [1102, 1491] | 1567 [1311, 1910] |
| Total gas volume (mL) | 947 [651, 1465] | 1201 [904, 1762] | 734 [495, 1157] |
| Not inflated lung tissue (%) | 43 [31, 55] | 34 [25, 45] | 50 [40, 61] |
| Poorly inflated lung tissue (%) | 30 [22, 39] | 27 [22, 37] | 31 [22, 40] |
| Well inflated lung tissue (%) | 25 [13, 35] | 34 [26, 42] | 15 [10, 27] |
| Over inflated lung tissue (%) | 0.01 [0.00, 0.13] | 0.02 [0.00, 0.19] | 0.01 [0.00, 0.09] |

Quantitative data are expressed as median [interquartile range] as appropriate. Categorical data are presented as N (number of subjects) and percentages (%).

**e-Table 5. Respiratory mechanics and gas exchange at 15 cmH_2_O of PEEP**

|  | Total Population  N = 238 | Phenotype 1 | Phenotype 2 |
| --- | --- | --- | --- |
|  |  | **“Non-Recruitable”**  N = 106 | **“Recruitable”**  N = 132 |
| Tidal Volume (mL) | 500 [420, 560] | 514 [441, 580] | 476 [414, 530] |
| Respiratory Rate (min^-1^) | 16 [14, 20] | 15 [14, 18] | 18 [15, 20] |
| Minute ventilation (L min^-1^) | 8.4 [7.2, 9.9] | 8.4 [7.2, 9.3] | 8.4 [7.2, 10.2] |
| End-inspiratory airway pressure (cmH_2_O) | 28 [25, 30] | 27 [24, 29] | 28 [25, 31] |
| Driving Pressure (cmH_2_O) | 13 [10, 15] | 12 [10, 14] | 14 [11, 17] |
| Respiratory system elastance (cmH_2_O mL-^1^) | 25 [20, 31] | 22 [17, 28] | 26 [21, 33] |
| Lung elastance (cmH_2_O mL^-1^) | 19 [15, 24] | 18 [14, 22] | 21 [16, 27] |
| Chest wall elastance (cmH_2_O mL^-1^) | 6 [4, 9] | 6 [4, 8] | 7 [4, 10] |
| Elastance derived Transpulmonary pressure (cmH_2_O) | 21 [18, 24] | 21 [17, 24] | 21 [18, 25] |
| Arterial pO_2_ (mmHg) | 97 [80, 123] | 92 [77, 110] | 102 [81, 134] |
| PaO_2_/FiO_2_ (mmHg) | 194 [152, 249] | 213 [174, 259] | 170 [136, 234] |
| Arterial pCO_2_ (mmHg) | 45 [40, 52] | 42 [37, 47] | 49 [42, 55] |
| Physiological dead space (%) | 62 [54, 73] | 54 [47, 60] | 72 [63, 79] |

Quantitative data are expressed as median [interquartile range] as appropriate. Categorical data are presented as N (number of subjects) and percentages (%).

**e-Table 6. Computed tomography data at 45 cmH_2_O of PEEP**

|  | Total Population  N = 238 | Phenotype 1 | Phenotype 2 |
| --- | --- | --- | --- |
|  |  | **“Non-Recruitable”**  N = 106 | **“Recruitable”**  N = 132 |
| Total lung tissue (g) | 1473 [1214, 1914] | 1364 [1148, 1634] | 1601 [1389, 2024] |
| Total lung gas volume (mL) | 2496 [1874, 3372] | 2904 [2366, 3948] | 2094 [1550, 2847] |
| Not inflated lung tissue (%) | 27 [16, 37] | 25 [17, 33] | 28 [16, 39] |
| Poorly inflated lung tissue (%) | 25 [19, 36] | 21 [17, 27] | 32 [22, 44] |
| Well inflated lung tissue (%) | 42 [32, 54] | 48 [40, 57] | 36 [28, 46] |
| Over inflated lung tissue (%) | 0.79 [0.13, 4.13] | 2.08 [0.53, 6.01] | 0.35 [0.05, 2.22] |
| Potentially Recruitable Lung (%) | 14.7 [7.5, 25.1] | 9.8 [5.0, 15.6] | 20.0 [11.0, 30.3] |

Quantitative data are expressed as median [interquartile range] as appropriate. Categorical data are presented as N (number of subjects) and percentages (%).

**e-Table 7. Latent Class Analysis identified, phenotype defining variables at PEEP 5.**

|  | Total Population  N = 238 | Phenotype 1 | Phenotype 2 | | *p* | *SMD* |
| --- | --- | --- | --- | --- | --- | --- |
|  |  | **“Non-Recruitable”**  N = 106 | | **“Recruitable”**  N = 132 |  |  |
| Physiological Dead Space (%) | 62 [54, 72] | 52 [46, 58] | | 70 [64, 80] | <0.001 | 2.117 |
| Alveolar Dead Space (%) | 22 [12, 29] | 11 [5, 19] | | 28 [23, 37] | <0.001 | 1.574 |
| Not inflated tissue (%) | 43 [31, 55] | 34 [25, 45] | | 50 [40, 61] | <0.001 | 0.959 |
| Respiratory System Elastance (cmH_2_O mL^-1^) | 26 [20, 32] | 23 [19, 28] | | 29 [24, 34] | <0.001 | 0.737 |
| Inhomogeneity (%) | 16 [13, 22] | 14 [12, 18] | | 18 [14, 26] | <0.001 | 0.720 |
| Total lung Tissue (g) | 1429 [1186, 1721] | 1302 [1102, 1491] | | 1567 [1311, 1910] | <0.001 | 0.655 |
| Driving Pressure (cmH_2_O) | 13 [10, 16] | 12 [9, 14] | | 14 [11, 16] | <0.001 | 0.607 |
| Lung elastance (cmH_2_O mL^-1^) | 19 [13, 25] | 14 [10, 21] | | 22 [16, 28] | <0.001 | 0.379 |
| Mechanical Power (J min^-1^) | 15 [11, 20] | 15 [11, 17] | | 16 [12, 22] | 0.065 | 0.304 |
| Peak Inspiratory Flow (L min^-1^) | 0.45 [0.38, 0.56] | 0.43 [0.38, 0.53] | | 0.46 [0.38, 0.62] | 0.152 | 0.179 |
| Arterial HCO_3_ (mEq L^-1^) | 26 [23, 30] | 25 [24, 29] | | 26 [23, 30] | 0.386 | 0.105 |
| Age (years) | 62 [48, 73] | 61 [47, 72] | | 62 [49, 73] | 0.558 | 0.088 |
| Heart Rate (min^-1^) | 90 [75, 106] | 90 [76, 101] | | 91 [75, 108] | 0.602 | 0.072 |
| Venous O_2_ Saturation (%) | 76 [71, 80] | 77 [71, 80] | | 76 [71, 80] | 0.689 | 0.004 |
| Body mass index (kg m^-2^) | 25 [22, 29] | 25 [23, 29] | | 25 [22, 29] | 0.205 | 0.207 |
| Tidal Volume/ Ideal Body Weight _PEEP 5_ (ml kg^-1^) | 7.7 [6.8, 8.8] | 7.8 [6.9, 8.8] | | 7.6 [6.7, 8.7] | 0.174 | 0.246 |
| Arterial – venous pCO_2_ (mmHg) | 5 [3, 6] | 5 [4, 7] | | 4 [2, 6] | <0.001 | 0.581 |
| paO_2_/FiO_2_ (mmHg) | 142 [104, 179] | 173 [146, 208] | | 114 [84, 143] | <0.001 | 0.981 |
| Well inflated tissue (%) | 25 [13, 35] | 34 [26, 42] | | 15 [10, 27] | <0.001 | 1.323 |

Quantitative data are expressed as median [interquartile range]. p values are given for the difference “Non-Recruitable” and “Recruitable” phenotype. Standardized Mean Differences (SMD) are given for the “Non-Recruitable” and “Recruitable” phenotype. paO2 - partial pressure of arterial oxygen; FiO2 - fraction of inspired oxygen; pCO2 - partial pressure of carbon dioxide.

**e-Figure 5. Profile Plot All Variables**

**
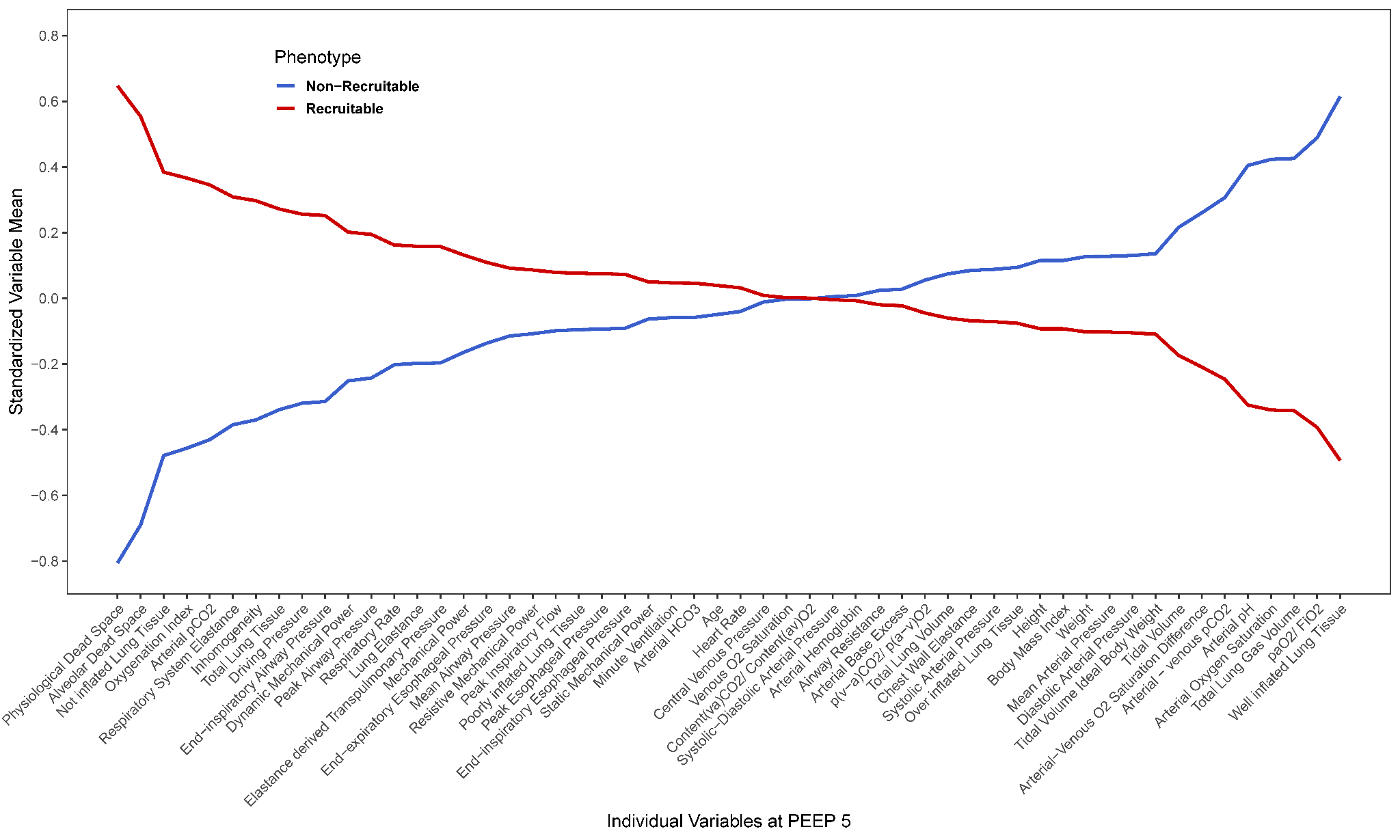
**

**e-Table 8. All Variables at PEEP 5 cmH_2_O employed for the LCA**

|  | Total Population  N = 238 | Phenotype 1 | Phenotype 2 | *p* | *SMD* |
| --- | --- | --- | --- | --- | --- |
|  |  | **“Non-Recruitable”**  N = 106 | **“Recruitable”**  N = 132 |  |  |
| Age (years) | 62 [48, 73] | 61 [47, 72] | 62 [49, 73] | 0.558 | 0.088 |
| Height (meters) | 2 [2, 2] | 2 [2, 2] | 2 [2, 2] | 0.146 | 0.208 |
| Weight (kg) | 75 [61, 85] | 75 [65, 90] | 72 [60, 83] | 0.060 | 0.231 |
| Body mass index (kg m^-2^) | 25 [22, 29] | 25 [23, 29] | 25 [22, 29] | 0.205 | 0.207 |
| Arterial Hemoglobin (g dL^-1^) | 9.80 [9.00, 10.60] | 9.90 [9.00, 10.88] | 9.75 [9.00, 10.40] | 0.809 | 0.015 |
| Heart Rate (min^-1^) | 90 [75, 106] | 90 [76, 101] | 91 [75, 108] | 0.602 | 0.072 |
| Central Venous Pressure (mmHg) | 11 [9, 14] | 11 [10, 13] | 11 [9, 14] | 0.924 | 0.020 |
| Systolic Arterial Pressure (mmHg) | 121 [112, 135] | 125 [114, 136] | 120 [111, 135] | 0.288 | 0.159 |
| Diastolic Arterial Pressure (mmHg) | 60 [52, 68] | 60 [54, 68] | 59 [50, 65] | 0.108 | 0.235 |
| Mean Arterial Pressure (mmHg) | 80 [73, 88] | 81 [75, 88] | 79 [70, 88] | 0.173 | 0.231 |
| Systolic – Diastolic Arterial Pressure (mmHg) | 63 [53, 75] | 64 [55, 74] | 62 [52, 76] | 0.827 | 0.009 |
| Venous O_2_ saturation (%) | 76 [71, 80] | 77 [71, 80] | 76 [71, 80] | 0.689 | 0.004 |
| Total lung volume (mL) | 2529 [2066, 3117] | 2572 [2073, 3212] | 2516 [2075, 3014] | 0.244 | 0.135 |
| Total lung tissue (g) | 1429 [1186, 1721] | 1302 [1102, 1491] | 1567 [1311, 1910] | <0.001 | 0.655 |
| Total lung gas volume (mL) | 947 [651, 1465] | 1201 [904, 1762] | 734 [495, 1157] | <0.001 | 0.816 |
| Not inflated lung tissue (%) | 43 [31, 55] | 34 [25, 45] | 50 [40, 61] | <0.001 | 0.959 |
| Poorly inflated lung tissue (%) | 30 [22, 39] | 27 [22, 37] | 31 [22, 40] | 0.253 | 0.173 |
| Well inflated lung tissue (%) | 25 [13, 35] | 34 [26, 42] | 15 [10, 27] | <0.001 | 1.323 |
| Over inflated lung tissue (%) | 0.01 [0.00, 0.13] | 0.02 [0.00, 0.19] | 0.01 [0.00, 0.09] | 0.004 | 0.165 |
| Inhomogeneity (%) | 16 [13, 22] | 14 [12, 18] | 18 [14, 26] | <0.001 | 0.720 |
| Tidal Volume (mL) | 500 [422, 560] | 520 [454, 597] | 478 [418, 535] | 0.002 | 0.397 |
| Tidal Volume/ Ideal Body Weight (mL kg^-1^) | 7.69 [6.77, 8.78] | 7.82 [6.92, 8.83] | 7.60 [6.69, 8.68] | 0.174 | 0.246 |
| Respiratory Rate (min^-1^) | 16 [14, 20] | 15 [14, 18] | 18 [15, 20] | 0.003 | 0.374 |
| Minute Ventilation (L min^-1^) | 8.40 [7.21, 9.90] | 8.40 [7.48, 9.12] | 8.40 [7.05, 10.21] | 0.480 | 0.107 |
| Mean airway pressure (cmH_2_O) | 10 [9, 12] | 10 [9, 11] | 10 [9, 12] | 0.060 | 0.207 |
| Peak airway pressure (cmH_2_O) | 25 [22, 29] | 24 [20, 26] | 26 [23, 30] | 0.002 | 0.452 |
| End-inspiratory airway pressure (cmH_2_O) | 18 [16, 21] | 17 [15, 20] | 19 [16, 22] | <0.001 | 0.596 |
| Peak Inspiratory Flow (L s^-1^) | 0.45 [0.38, 0.56] | 0.43 [0.38, 0.53] | 0.46 [0.38, 0.62] | 0.152 | 0.179 |
| Airway Resistance (cmH_2_O L^-1^ s^-1^) | 14 [11, 18] | 14 [11, 19] | 14 [11, 18] | 0.717 | 0.043 |
| Driving Pressure (cmH_2_O) | 13 [10, 16] | 12 [9, 14] | 14 [11, 16] | <0.001 | 0.607 |
| Peak esophageal pressure (cmH_2_O) | 18 [15, 21] | 18 [15, 20] | 18 [16, 21] | 0.425 | 0.168 |
| End-inspiratory esophageal pressure (cmH_2_O) | 17 [14, 20] | 17 [14, 19] | 17 [14, 20] | 0.341 | 0.164 |
| End-expiratory esophageal pressure (cmH_2_O) | 14 [10, 17] | 12 [10, 16] | 14 [11, 17] | 0.009 | 0.258 |
| Lung Elastance (cmH_2_O mL^-1^) | 19 [13, 25] | 14 [10, 21] | 22 [16, 28] | <0.001 | 0.379 |
| Respiratory System Elastance (cmH_2_O mL^-1^) | 26 [20, 32] | 23 [19, 28] | 29 [24, 34] | <0.001 | 0.737 |
| Chest Wall Elastance (cmH_2_O mL^-1^) | 6 [4, 9] | 6 [4, 10] | 6 [4, 9] | 0.180 | 0.162 |
| Elastance derived Transpulmonary pressure (cmH_2_O) | 13 [10, 16] | 11 [8, 15] | 14 [12, 18] | <0.001 | 0.376 |
| Mechanical Power (J min^-1^) | 15 [11, 20] | 15 [11, 17] | 16 [12, 22] | 0.065 | 0.304 |
| Resistive Mechanical Power (J min^-1^) | 5 [3, 8] | 5 [3, 7] | 6 [4, 8] | 0.283 | 0.197 |
| Dynamic Mechanical Power (J min^-1^) | 5 [4, 7] | 5 [3, 6] | 5 [4, 8] | 0.002 | 0.472 |
| Static Mechanical Power (J min^-1^) | 4 [3, 5] | 4 [4, 5] | 4 [3, 5] | 0.531 | 0.116 |
| Arterial pH | 7.38 [7.33, 7.42] | 7.39 [7.36, 7.45] | 7.35 [7.31, 7.40] | <0.001 | 0.782 |
| Arterial pCO_2_ (mmHg) | 44 [39, 52] | 41 [37, 45] | 49 [42, 55] | <0.001 | 0.854 |
| PaO_2_/FiO_2_ (mmHg) | 142 [104, 179] | 173 [146, 208] | 114 [84, 143] | <0.001 | 0.981 |
| Arterial O_2_ saturation (%) | 94 [90, 96] | 95 [93, 96] | 91 [89, 94] | <0.001 | 0.844 |
| Arterial-venous O_2_ saturation difference (%) | 17 [13, 21] | 18 [14, 23] | 16 [11, 19] | 0.001 | 0.481 |
| Alveolar Dead Space (%) | 22 [12, 29] | 11 [5, 19] | 28 [23, 37] | <0.001 | 1.574 |
| Physiological Dead Space (%) | 62 [54, 72] | 52 [46, 58] | 70 [64, 80] | <0.001 | 2.117 |
| Content_v-a_CO_2_/ Content_a-v_O_2_ | 0.61 [0.23, 0.98] | 0.64 [0.42, 0.94] | 0.57 [0.08, 1.01] | 0.133 | 0.003 |
| Arterial-venous pCO_2_ difference (mmHg) | 5 [3, 6] | 5 [4, 7] | 4 [2, 6] | <0.001 | 0.581 |
| (ΔpCO_2_)_v-a_/(ΔpO_2_)_a-v_ | 0.17 [0.10, 0.25] | 0.16 [0.11, 0.24] | 0.18 [0.07, 0.25] | 0.778 | 0.104 |
| Arterial Base Excess (mEq L^-1^) | 1.55 [-1.40, 5.00] | 1.55 [-1.10, 4.95] | 1.55 [-2.00, 5.00] | 0.768 | 0.050 |
| Arterial HCO_3_ (mEq L^-1^) | 26 [23, 30] | 25 [24, 29] | 26 [23, 30] | 0.386 | 0.105 |
| Oxygenation Index (cmH_2_O mmHg^-1^) | 6.96 [5.49, 10.83] | 5.69 [4.87, 6.86] | 9.22 [6.69, 12.56] | <0.001 | 0.923 |

Quantitative data are expressed as median [interquartile range] as appropriate. Categorical data are presented as N (number of subjects) and percentages (%). p values are given for the difference between “Non-Recruitable” and “Recruitable” phenotype. Standardized Mean Differences (SMD) are given for the difference between “Non-Recruitable” and “Recruitable” phenotype.

Abbreviations: Content _v-a_ CO_2_/ Content _a-v_O_2_: venous-to-arterial carbon dioxide content to arterial-to-venous oxygen content ratio; (ΔpCO_2_)_v-a_ /(ΔpO_2_)_a-v_ : venous-to-arterial carbon dioxide tension difference to arterial-to-venous oxygen tension difference ratio; Δ: delta, difference.

**
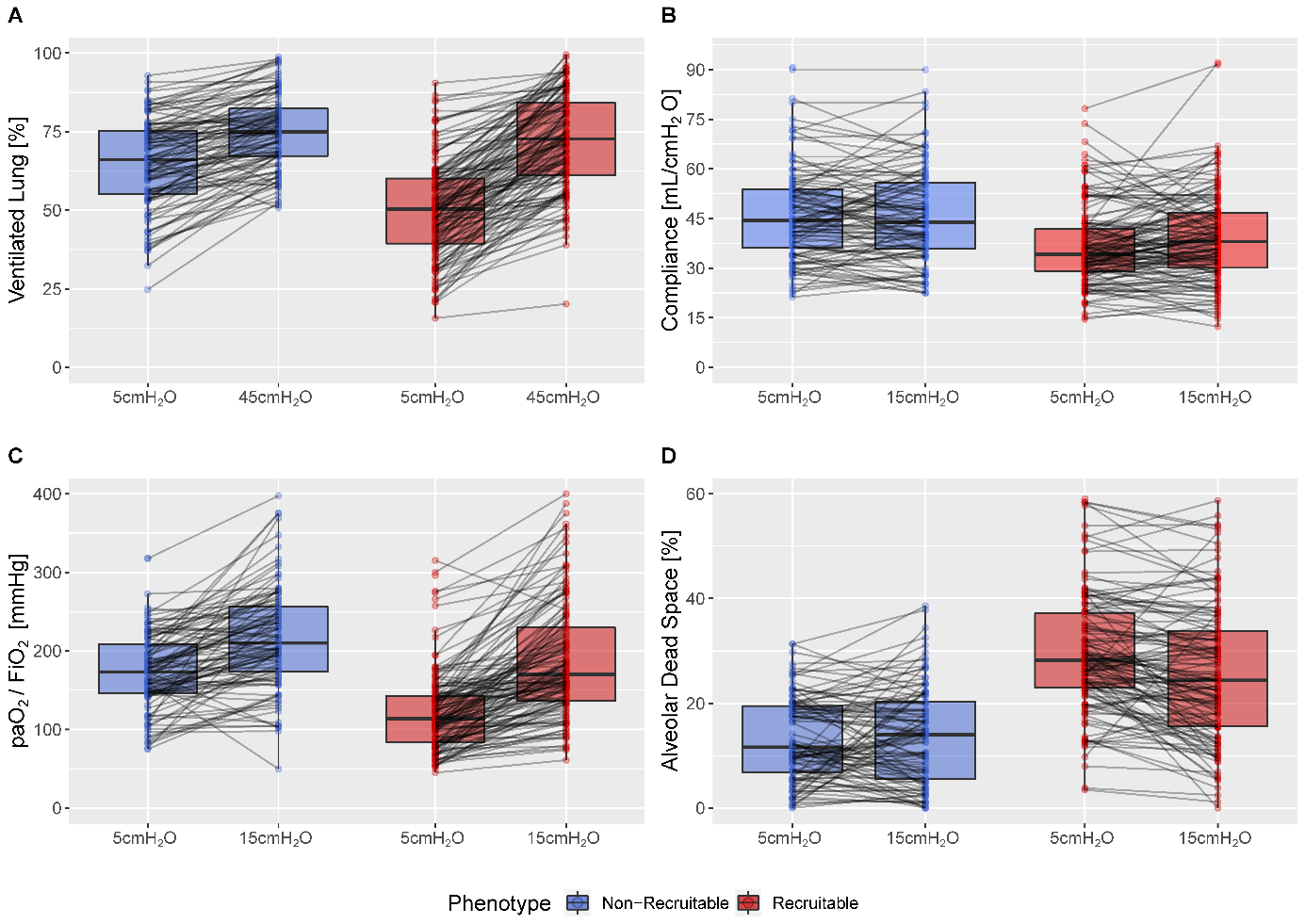
e-Figure 6. Response to recruitment maneuver for LCA derived phenotypes (with trend lines)**

Box-plots present (A) the amount of ventilated lung, defined as the cumulative CT-graphicly inferred poorly, well and over-inflated lung, (B) the respiratory system compliance, (C) the paO_2_/ FiO_2_ ratio and (D) the alveolar dead space at two stages of a recruitment maneuver for the *non-recruitable* and *recruitable* LCA phenotypes. (A) was measured during a inspiratory hold maneuver at an end-inspiratory airway pressures of 5 and 45 cmH_2_O; (B), (C) and (D) were measured under positive end-expiratory pressures of 5 and 15 cmH_2_O. Black trendlines connect values pre and post recruitment maneuver.

**e-Table 9. Full specification of mixed-effect models**

**A. Ventilated Lung**

|  | **Variance** | **Standard Deviation** | |
| --- | --- | --- | --- |
| **Random Effect** | | | |
| ***Intercept*** | 143.87 | 11.99 | |
| ***Residual*** | 69.37 | 8.33 | |
|  | **Estimate** | **95% Confidence Interval** | **p** |
| **Fixed Effects** | | | |
| ***Intercept*** | 64.65 | 61.87, 67.42 | <2 e^-16^ |
| **PEEP 15** | 10.31 | 8.06, 12.55 | <2 e^-16^ |
| **Recruitable Phenotype** | -14.56 | -18.29, -10.83 | 2.4 e^-13^ |
| **Interaction: PEEP 15 – Recruitable Phenotype** | 11.78 | 8.77, 14.79 | 4.6 e^-13^ |

**B. Compliance**

|  | **Variance** | **Standard Deviation** | |
| --- | --- | --- | --- |
| **Random Effect** | | | |
| ***Intercept*** | 146.58 | 12.11 | |
| ***Residual*** | 63.36 | 7.96 | |
|  | **Estimate** | **95% Confidence Interval** | **p** |
| **Fixed Effects** | | | |
| ***Intercept*** | 49.99 | 43.35, 48.63 | <2 e^-16^ |
| **PEEP 15** | 2.08 | 0.64, 3.51 | 0.00493 |
| **Recruitable Phenotype** | -9.14 | -12.56, -5.73 | 3.41 e^-07^ |

**C. paO_2_/ FiO_2_ Ratio**

|  | **Variance** | **Standard Deviation** | |
| --- | --- | --- | --- |
| **Random Effect** | | | |
| ***Intercept*** | 2581 | 50.81 | |
| ***Residual*** | 1757 | 41.92 | |
|  | **Estimate** | **95% Confidence Interval** | **p** |
| **Fixed Effects** | | | |
| ***Intercept*** | 175.03 | 162.51, 187.55 | < 2e^-16^ |
| **PEEP 15** | 45.56 | 34.24, 56.89 | 1.18 e^-13^ |
| **Recruitable Phenotype** | -51.81 | -68.62, -35.00 | 4.16 e^-09^ |
| **Interaction: PEEP 15 – Recruitable Phenotype** | 22.77 | 7.59, 37.95 | 0.00363 |

**D. Alveolar Dead Space**

|  | **Variance** | **Standard Deviation** | |
| --- | --- | --- | --- |
| **Random Effect** | | | |
| ***Intercept*** | 82.97 | 9.11 | |
| ***Residual*** | 51.60 | 7.18 | |
|  | **Estimate** | **95% Confidence Interval** | **p** |
| **Fixed Effects** | | | |
| ***Intercept*** | 13.55 | 11.34, 15.75 | < 2e^-16^ |
| **PEEP 15** | 0.35 | -1.60, 2.29 | 0.72712 |
| **Recruitable Phenotype** | 16.03 | 13.07, 18.99 | < 2e^-16^ |
| **Interaction: PEEP 15 – Recruitable Phenotype** | -3.49 | -6.10, -0.89 | 0.00917 |

**e-Figure 7. Dependency of the amount of potentially recruitable lung on the PaO_2_/ FiO_2_ ratio depending on the pulmonary phenotype**


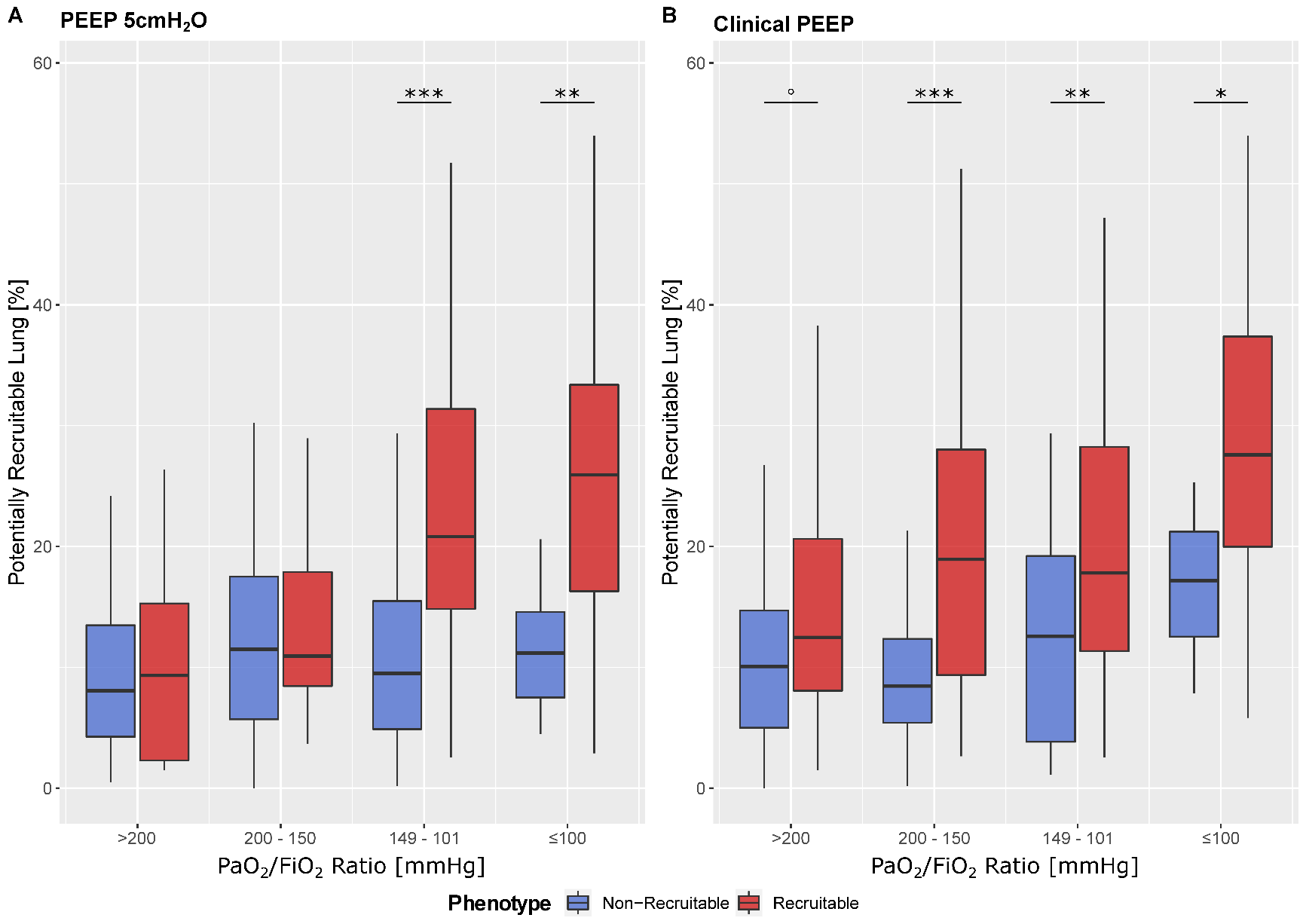


Stratification of box-plots by PEEP. p-values °<0.1, *<0.05, **<0.01, ***<0.001, for differences between pulmonary phenotypes at same PaO_2_/ FiO_2_ interval.

**e-Figure 8. Distribution of PaO_2_/ FiO_2_ Ratios in pulmonary phenotypes stratified by underlying PEEP**


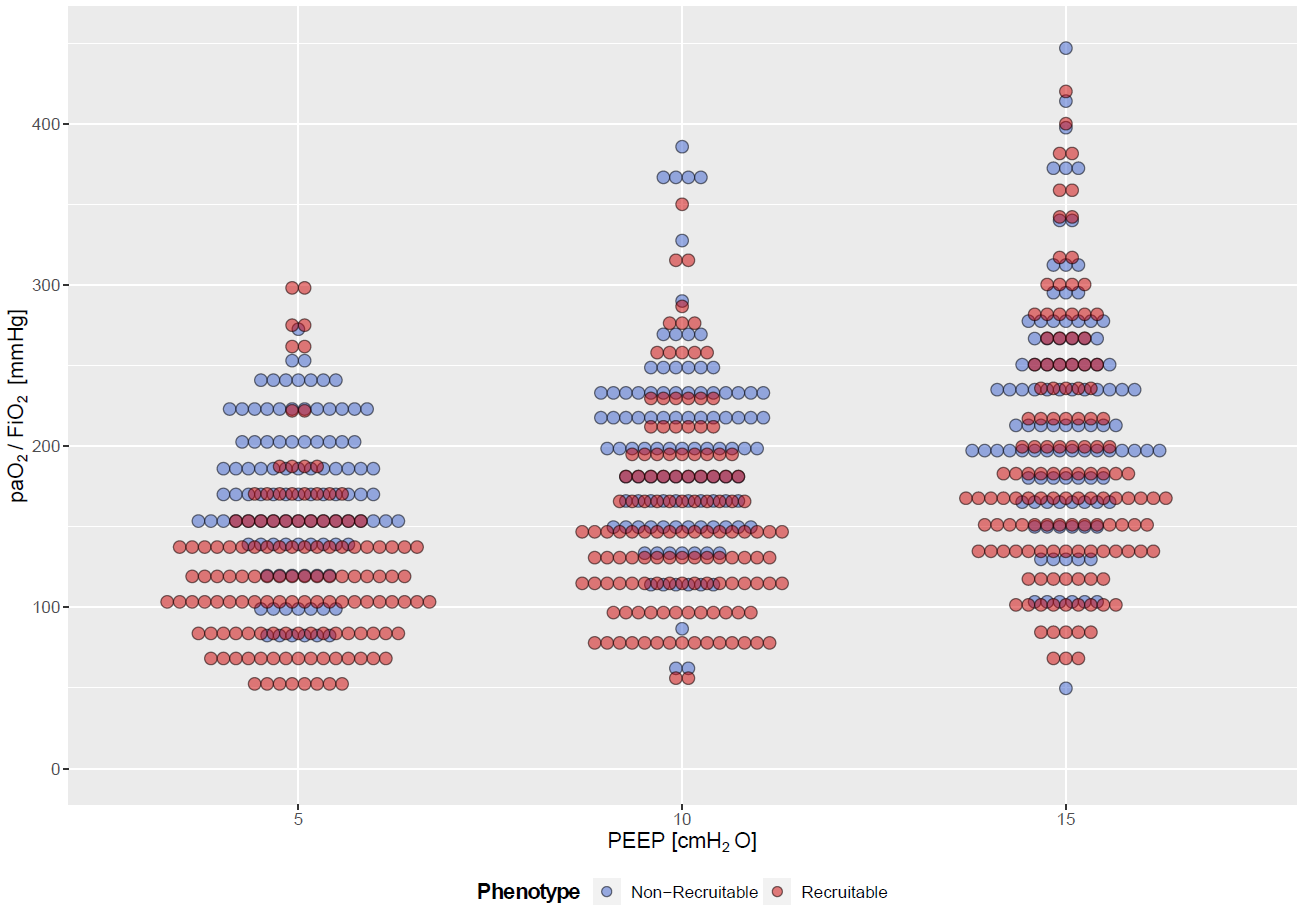


**e-Figure 9. Survival Analysis for Patients with Moderate ARDS**

**
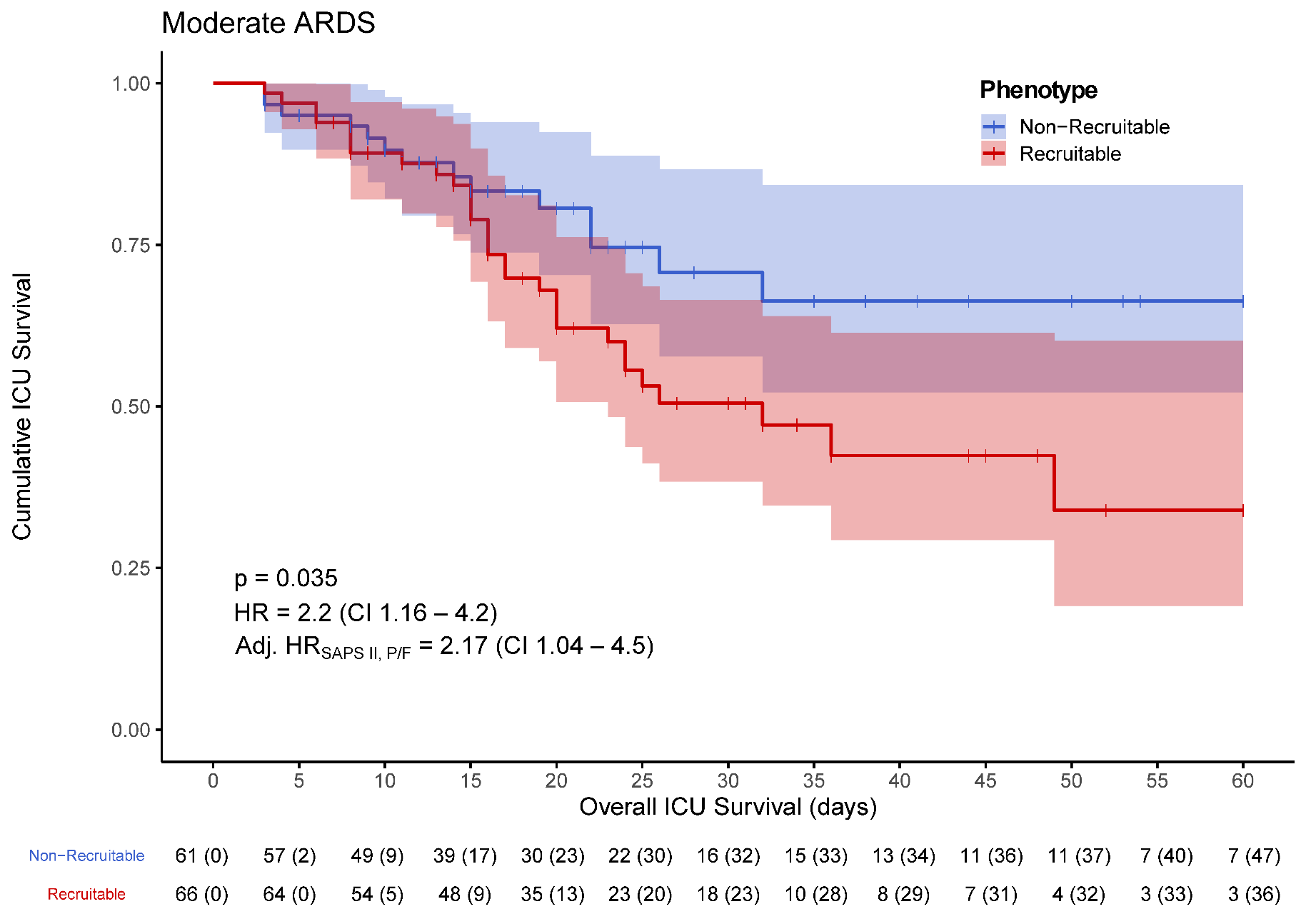
**

Kaplan-Meier curve for 60-day intensive care unit survival stratified by Latent Class Analysis (LCA) derived phenotype. Non-recruitable and recruitable phenotypes are plotted in blue and red colors respectively, shaded areas represent the 95% Confidence Interval (CI). The computed hazard ratio (HR) assesses the recruitable using the non-recruitable phenotype as, 95% CI is given in parentheses. HRs are modelled by means of a Fine and Gray competing risk analysis. Censoring reflects patients having left the ICU alive. The underlying table presents the patients at risk per time point with the number of censored patients given in parentheses.

**e-Figure 10. Receiver Operating Characteristics Curves of Phenotype Prediction Models**


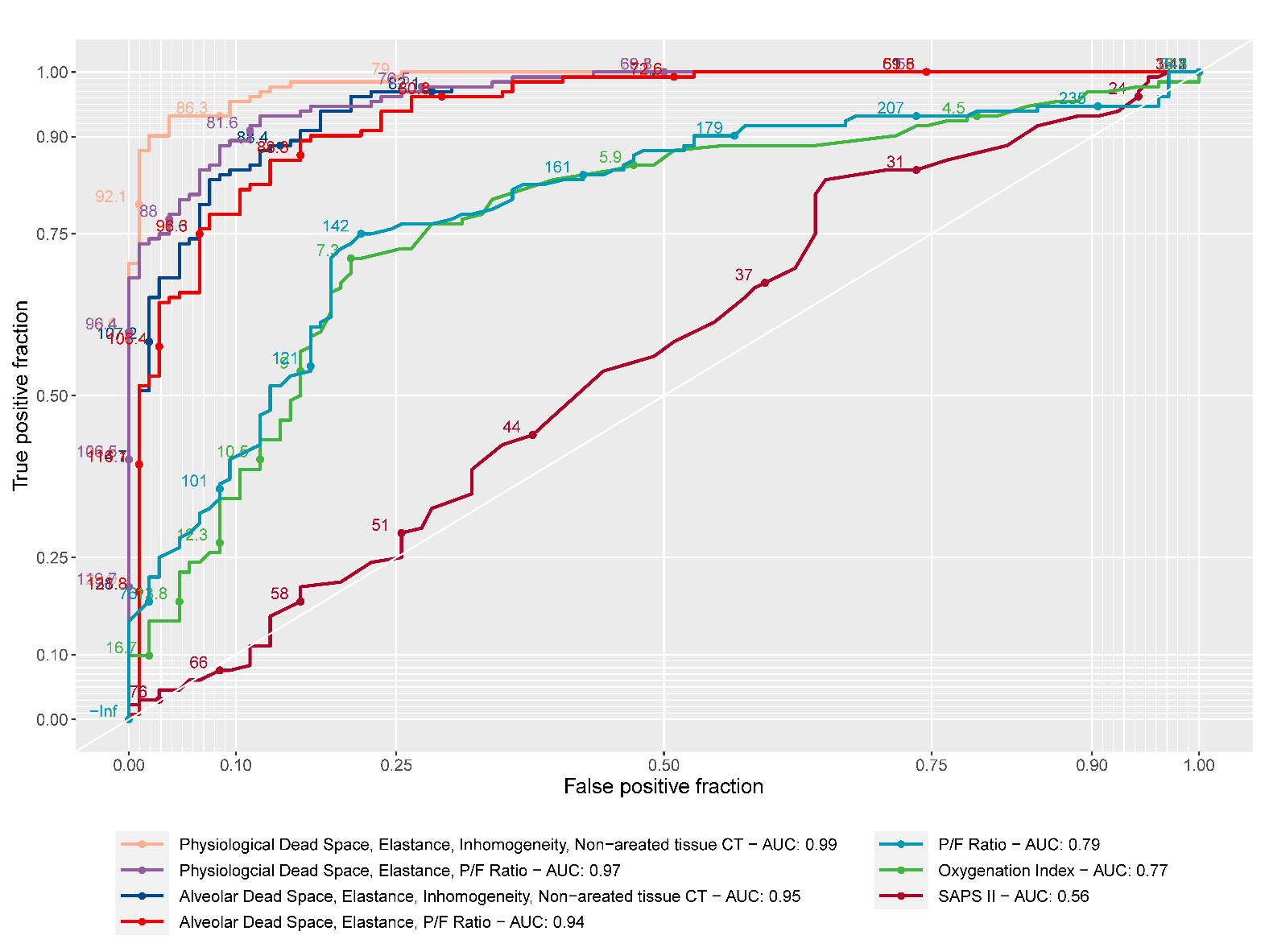
**e-Table 10. Area Under the Receiver Operating Curves (AUROCs) for the LASSO and nested GLM inferred models and classic severity scores.**

|  | **AUROC** | **95% CI** | **p vs. Model 1** | **p vs. Model 4** |
| --- | --- | --- | --- | --- |
| **Model 1:** Elastance_Respiratory System,_ Dead Space_Physiological_, Inhomogeneity, Non-aerated tissue | 0.99 | 0.975 – 0.995 |  | 0.001 |
| **Model 2:** Elastance_Respiratory System,_ Dead Space_Alveolar_, Inhomogeneity, Non-aerated tissue | 0.97 | 0.949 – 0.983 | 0.006 | 0.017 |
| **Model 3:** Elastance_Respiratory System,_ Dead Space_Physiological_, P/F Ratio | 0.95 | 0.919 – 0.974 | 0.007 | 0.046 |
| **Model 4:** Elastance_Respiratory System,_ Dead Space_Alveolar_, P/F Ratio | 0.94 | 0.905 – 0.964 | 0.001 |  |
| **P/F Ratio** | 0.79 | 0.727 – 0.843 | <0.0001 | <0.0001 |
| **Oxygenation Index** | 0.77 | 0.707 – 0.828 | <0.0001 | <0.0001 |
| **SAPS II** | 0.56 | 0.481 – 0.633 | <0.0001 | <0.0001 |

**e-Table 11. LASSO + Nested Generalized Logistic Regression for “Elastance_Respiratory System,_ Dead Space_Physiological_ and P/F ratio” at PEEP 5**

|  | Estimate | Std. Error | Z value | p |
| --- | --- | --- | --- | --- |
| Intercept | -21.72 | 3.78 | -5.747 | <0.001 |
| Dead Space _Physiological_ | 0.322 | 0.053 | 6.124 | <0.001 |
| Elastance _Respiratory System_ | 0.195 | 0.044 | 4.402 | <0.001 |
| P/F Ratio | -0.016 | 0.005 | -3.450 | <0.001 |

**e-Table 12. LASSO + Nested Generalized Logistic Regression for “Elastance_Respiratory System,_ Dead Space_Alveolar_ and P/F ratio” at PEEP 5**

|  | Estimate | Std. Error | Z value | p |
| --- | --- | --- | --- | --- |
| Intercept | -6.973 | 1.421 | -4.905 | <0.0001 |
| Dead Space _Alveolar_ | 0.241 | 0.036 | 6.631 | <0.0001 |
| Elastance _Respiratory System_ | 0.198 | 0.038 | 5.217 | <0.0001 |
| P/F Ratio | -0.018 | 0.004 | -3.991 | <0.001 |

**e-Table 13. LASSO + Nested Generalized Logistic Regression for “Elastance_Respiratory System,_ Dead Space_Physiological_, Inhomogeneity, Non-aerated tissue” at PEEP 5**

|  | Estimate | Std. Error | Z value | p |
| --- | --- | --- | --- | --- |
| Intercept | -43.495 | 7.686 | -5.659 | <0.001 |
| Dead Space _Physiological_ | 0.494 | 0.0887 | 5.571 | <0.001 |
| Elastance _Respiratory System_ | 0.204 | 0.062 | 3.295 | <0.001 |
| Inhomogeneity | 0.129 | 0.0513 | 2.520 | 0.012 |
| Non-aerated tissue | 0.150 | 0.032 | 4.648 | <0.001 |

**e-Table 14. LASSO + Nested Generalized Logistic Regression for “Elastance_Respiratory System,_ Dead Space_Alveolar_, Inhomogeneity, Non-aerated tissue” at PEEP 5**

|  | Estimate | Std. Error | Z value | p |
| --- | --- | --- | --- | --- |
| Intercept | -14.946 | 2.217 | -6.741 | <0.001 |
| Dead Space _Alveolar_ | 0.267 | 0.0423 | 6.311 | <0.001 |
| Elastance _Respiratory System_ | 0.172 | 0.043 | 4.013 | <0.001 |
| Inhomogeneity | 0.08 | 0.039 | 2.074 | 0.038 |
| Non-aerated tissue | 0.094 | 0.019 | 4.943 | <0.001 |
